# Implementation of MF block in CNN for advanced REB fault diagnosis

**DOI:** 10.1038/s41598-025-01780-y

**Published:** 2025-05-25

**Authors:** M. Pandiyan, Narendiranath Babu T.

**Affiliations:** School of Mechanical Engineering, Vellore Institute of Technology, Vellore, Tamil Nadu 631 014 India

**Keywords:** Rolling element bearing, Fault diagnosis, C-CNN, Multi-feature block, BCE loss function, Mechanical engineering, Aerospace engineering

## Abstract

Rolling element bearings (REBs) are crucial components in various industrial applications. The bearing faults occur due to prolonged operation, overloading, high speed, and inadequate lubrication. A bearing failure can lead to significant downtime and huge maintenance costs for the machines. Hence, industries require condition monitoring to reduce costs. This study presents an automated detection approach for diagnosing faults in REBs using a Customized Convolutional Neural Network (C-CNN). This work focuses on vibration signals sampled at 12,800 Hz and 5120 Hz as input data to perform the fault diagnosis of bearings. Further, the Multi Feature (MF) block has been used in the architecture of the C-CNN model for better accuracy. By incorporating techniques such as batch normalization and dropout, the model has improved stability and prevented overfitting. Using the Balance Cross-Entropy (BCE) loss function for training has helped the model optimize prediction accuracy by minimizing the difference between the actual class and the predicted class probabilities. A comparison with models such as MSCNN, CNN, RF, DBN, KNN, ANN, SVM, LSTM, ResNet and SqueezeNet was carried out. The proposed C-CNN model has been found to well perform other classifiers in accurately recognizing bearing faults, achieving an excellent accuracy of 95% and 93.5% on 12,800 and 5120 Hz datasets, respectively. Statistical significance tests and error bars validate the robustness of the model’s performance. Extensive experiments were conducted to evaluate the impact of sampling frequency on diagnostic accuracy, hyperparameter tuning strategies, and model robustness under different noise levels and operating conditions. Furthermore, computational complexity analysis, including FLOPs estimation, was performed to assess real-time applicability. The findings indicate that the C-CNN approach is a reliable and efficient solution for bearing fault classification, offering significant practical implications for industrial condition monitoring systems and helping to prevent plant shutdown.

## Introduction

Bearings are essential components found in various rotating machinery like motors, turbines, pumps, and gearboxes, playing a vital role in ensuring the smooth operation as well as reliability of industrial systems^1,2^. However, due to the severe conditions they operate under, bearings are susceptible to various types of issues such as surface wear, pitting, cracking, and misalignment. In case the issues are not identified, they can lead to costly machinery downtime, production losses, and safety hazards. Hence, timely and precise detection of bearing problems is crucial for proactive maintenance and optimal performance of industrial equipment^3^. Effective fault diagnosis is essential for reducing downtime, extending machinery’s lifespan, and maximizing operational efficiency. Unnoticed faults can escalate into catastrophic failures, leading to significant financial losses and safety risks. Therefore, proactive maintenance strategies enabled by accurate fault diagnosis are critical for ensuring continuous operations and reducing maintenance expenses^4^.

Despite the acknowledged importance of diagnosing bearing faults, several challenges persist in developing effective diagnostic solutions. One major problem is the complexity of fault signatures, which can vary significantly depending on factors such as load conditions, operating speeds, and environmental factors^5^. Moreover, background noise often combines with fault signals, making it challenging to distinguish genuine fault indicators from irrelevant sensor data fluctuations^6^. Additionally, traditional diagnostic methods like vibration analysis and acoustic monitoring may have limitations in accurately identifying early-stage faults or faults occurring in specific bearing components. Consequently, there is a rising need for advanced diagnostic techniques that are capable of reliably detecting and classifying various bearing faults in diverse operational environments^7^.

The author^8^ has developed a bearing fault diagnosis method using the Efficient Convolutional Module (ECM), incorporating channel attention and MSCNN for fault prediction. This method shows promise for accurate and versatile fault diagnosis in rolling bearings, offering practical insights for real-world applications. But the intricacy of the model’s architecture may lead to higher computational demands in real-time applications. Meanwhile, the research introduced Diffusion Transformation Model (DTM) Bearing, a novel approach for detecting bearing faults using DTM^9^. This innovative approach combines diffusion model concepts with transformation techniques to potentially advance the effectiveness of bearing fault diagnostics in real-world applications. Furthermore, a fine-grained, multi-scale Kolmogorov entropy and whale-optimized multi-class support vector machine (FGMKE-WOA-MSVM) has been introduced^10^. Calculated Kolmogorov entropy at multiple scales helps generate a multi-dimensional feature vector. Addressing challenges in parameter selection for the MSVM model, the Whale Optimization Algorithm (WOA) optimizes kernel function parameters and penalty factors, leading to an optimal WOA-MSVM strategy. Moreover, multi-sensor data fusion has well performed single-sensor methods, significantly enhancing fault prediction across different models^11^. Explainable Artificial Intelligence (XAI) methods like Local Interpretable Model Agnostic Explanation (LIME) and RF have made fault diagnoses easier to understand. Employing explainable artificial intelligence methods could increase the complexity to implementation.

In recent years, convolutional neural networks (CNN) have become a major focus in defect identification. Plenty of investigators have correlated the failure signals of bearings with convolutional networks for diagnostic purposes. A new fault diagnosis model called Deep Convolutional Generative Adversarial Network (DLGAN) has been created by researchers for sliding bearing-rotor systems in nuclear power plants^12^. Researchers have designed the model to enhance the diagnosis of bearing faults by utilizing Deep Convolutional Generative Adversarial Network (DL) techniques. To address the lack of a universal model, the researchers have set up experiments to collect diverse fault data under different operating conditions and fault types. Moreover, the researchers have employed the CBAR model, which integrates the Convolutional Block Attention Module (CBAM) and Residual Network (ResNet), to diagnose bearing faults^13^. Experimental results have shown a remarkable 100% accuracy rate in diagnosing single-point and mixed faults using an open-source dataset. This method, which employs wavelet denoising and algebraic techniques for effective signal preprocessing, offers benefits such as faster data processing, a smaller model structure, and high diagnostic accuracy.

The experts used Deep Independent Component Analysis (ICA) combined with Variational Modal Decomposition (VMD) enhances signal decomposition and noise reduction. The VMD-ICA approach has demonstrated high accuracy in experimental evaluations, making it a reliable tool for industrial fault diagnosis^14^. The integration of 1D-CNN with deep VMD-ICA improves defect feature extraction, achieving high diagnostic accuracy. Fast Fourier techniques further refine vibration analysis, making this approach highly reliable for industrial condition monitoring^15^. Further the authors highlight HHT as a powerful tool for diagnosing nonlinear and non-stationary bearing faults, well performing traditional methods. Wavelet thresholding enhances noise reduction, while EEMD improves IMF extraction. FFT aids frequency-domain analysis, and Mahalanobis distance with cosine similarity enhances fault classification. Combining these techniques ensures accurate and reliable fault diagnosis in rotating machinery^16^. The dimension theory technique, combined with the matrix method (DTMM), provides insights into how local defects impact bearing dynamics. Analyzing internal radial clearance helps in understanding defect behavior under different conditions. Noise reduction using modulation signal bi-spectrum (MSB) enhances signal clarity for fault detection^17^. Experimental investigations highlight that linear tip relief tooth profile modification significantly affects vibration behavior. Taguchi optimization techniques help identify an optimal combination of addendum, backlash, and tooth profile modification to minimize vibrations. The findings emphasize the importance of precise geometric parameter selection in achieving quieter and more efficient gear systems^18,19^.

Furthermore, researchers also used the Gramian Angular Field (GAF) image coding, CNN, and ELM (Extreme Learning Machine) methods^20^. The method involves converting vibration signals into 2-D GAF images, using an enhanced CNN for efficient identification of fault features, and ELM for accurate classification. Comparative analysis has highlighted significant improvements over traditional methods, demonstrating that the performance of the suggested approach is accurately predicting various types of faults. Next, experts have introduced a 1D-Vision Transformer (1D-ViT) encoder framework^21^, which applies the 1D-ViT model directly to one dimensional data without the need for time–frequency conversion. By optimizing the method through encoder ablation experiments, a better average accuracy with low computational demands and parameter counts has been achieved, showcasing superior noise resistance. Compared with the traditional models, the 1D-ViT model has demonstrated significant advantages in space utilization, accuracy, computational efficiency, and noise handling, emphasizing its effectiveness for fault diagnosis in industrial applications. The proposed fault diagnosis method’s scalability in large-scale industrial applications remains unexplored due to potential hardware or software limitations.

This study proposed an intelligent fault identification method for taper_roller bearing defects using an optimized Extreme Learning Machine (ELM). Swarm Decomposition (SWD) removes noise, and Permutation Entropy (PE) selects the most relevant mode. A relief algorithm extracts and ranks features, while an opposition-based slime mould algorithm optimizes ELM parameters^22^. Next the authors developed a deep learning-based defect identification method for the Pelton wheel using an optimized Time-Varying Filter Empirical Mode Decomposition (TVF-EMD) and a Convolutional Neural Network (CNN). The Amended Grey Wolf Optimization (AGWO) algorithm optimizes TVF-EMD parameters, enhancing signal decomposition. A scalogram-based dataset is used to train the CNN model, achieving 100% accuracy. The AGWO algorithm’s efficiency is validated against benchmark functions and the Wilcoxon test, proving its superiority over other optimization methods^23^. Moreover, the experts introduced an advanced fault diagnosis method for worm gearboxes using an adaptive Convolutional Neural Network (CNN) optimized with the Amended Gorilla Troop Optimization (AGTO) algorithm. The vibration and acoustic signals are transformed into 2D time–frequency images using the Morlet wavelet function, which are then analyzed by AGTO-CNN. The AGTO algorithm is validated against benchmark functions and the Wilcoxon test, demonstrating superior performance. This approach can be developed to diagnose other mechanical components^24^. A study proposed a fault identification method for direct-shift gearboxes using Variational Mode Decomposition (VMD) and Convolutional Neural Networks (CNN). VMD decomposes vibration signals into intrinsic modes. The selected mode is converted into a scalogram, providing a time–frequency representation of the signal. These scalograms are then formed into image matrices and CNN classifies faults like healthy, pitting, and chipping. The method achieved 100% accuracy, well performing other neural networks^25^.

The innovative multi-feature block design of LMFRNet, a lightweight CNN model, has significantly reduced the computational demands and complexity of the model^26^. It has achieved exceptional accuracy and demonstrated both high performance and efficient resource utilization. Extensive experimentation also has provided valuable insights into optimizing critical hyper parameters for effective model training. The authors have expanded the application of the adaptable classification model to various environments with constrained resources, including embedded systems and Internet of Things (IoT) applications. Experts have employed various approaches for diagnosing bearing faults, ranging from existing signal processing methods to advanced ML algorithms^27,28^. Condition monitoring systems widely use vibration analysis, a prominent technique that detects bearing faults by analyzing the amplitude modulation and frequency spectra characteristics of vibration signals^29^. Methods like acoustic emission analysis, thermography, and oil analysis, in addition to vibration analysis, offer further insights into bearing condition^6,30^. However, these approaches may have limitations in detecting early-stage faults or faults occurring in bearings that are inaccessible under heavy loads.

### Problem statement

The literature review underscores the difficulties in defect identification within industrial settings, including significant data unpredictability, elevated noise levels, and substantial processing requirements. Furthermore, hybrid approaches are intricate to execute, and early-stage problems in bearings are challenging to identify. Moreover, inadequate examination of hardware or software may impact scalability, resulting in heightened maintenance expenses and lower accuracy rates. Several researchers have employed traditional CNN architectures, which typically use normal ReLU activation functions in the preliminary phases to mitigate financial losses in industries and enhance the accuracy of bearing defect predictions. Additionally, there is an inadequate application of regularization techniques to mitigate overfitting, among other issues. Also, the output layers of regular CNNs might not always give a clear probabilistic interpretation of predictions, which is important for understanding how reliable and accurate the diagnostic results are. These restrictions highlight the necessity for a customized CNN architecture, particularly one designed for effective and precise fault detection in REBs , as well as the creation of resilient diagnostic models that can generalize well across diverse datasets and equipment set-ups.

### Objective

The major contribution of this work is as follows:

- To use CNN in the field of automated bearing fault diagnosis.
- To propose the C-CNN model’s architecture consisting of six stages with MF blocks.
- To effect modifications to the loss function and certain stages so that improved diagnostic accuracy and robustness is achieved.
- To frame the DL (Deep Learning) methodologies to accomplish efficient and precise fault detection in bearings.
- To x

Hence, this work aims to enhance condition monitoring of the bearing fault diagnosis using the C-CNN techniques.

The sub-sequent sections of the paper are formulated as follows: Section "Experimental set-up and procedure" focuses on the experimental work and includes a detailed description of the dataset. Section "Methodology" discusses the methodologies employed in this study. Section "Results and discussion" represents the results and discussion, analyzing the performance of the proposed method and its implications. Finally, Section "Long Short-Term Memory (LSTM)" provides the conclusion that summarizes the findings.

## Experimental set-up and procedure

**Fig. 1:**
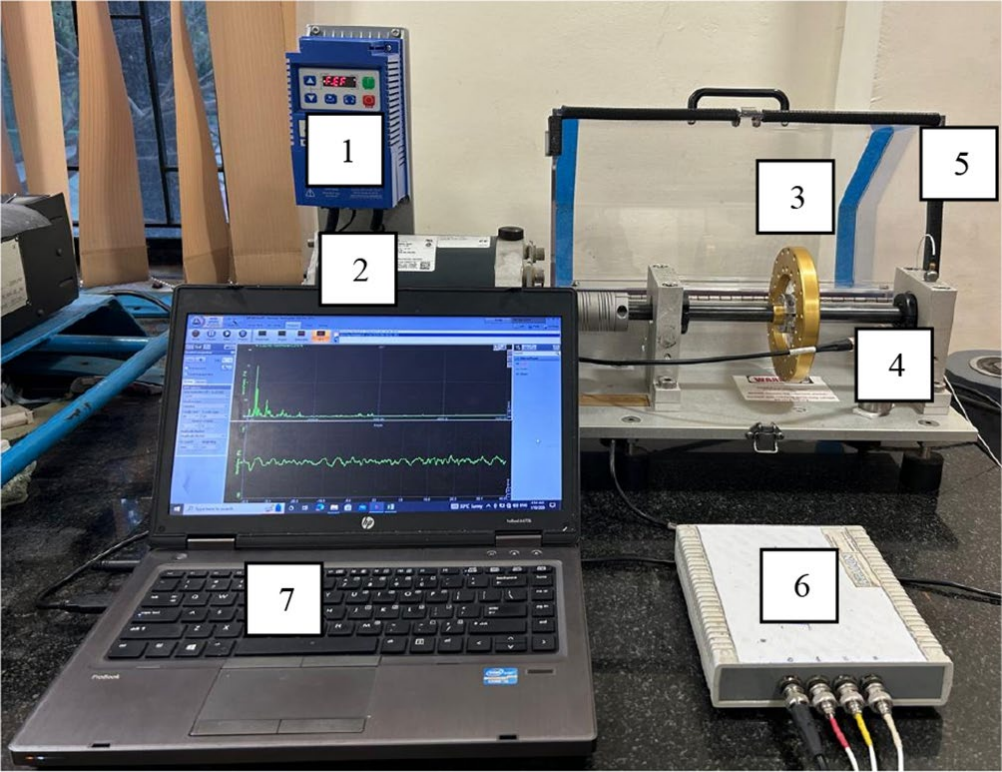
Experimental set-up: 1. Frequency variable unit, 2. Electric motor with Tachometer, 3. Balance rotor, 4. Test bearing and housing, 5. Accelerometer, 6. DAQ System, and 7. Display and Storage device.

In this experiment, the various speed conditions have been simulated using a bearing fault simulator test rig. Then the vibrational signals have been recorded using an IEPE accelerometer and a data acquisition system. The data acquisition system has been integrated with DEWEsoft to collect vibration data at different sampling rates. Figure 1 illustrates the experimental set-up to diagnose both individual and multiple bearing faults.

**Fig. 2:**
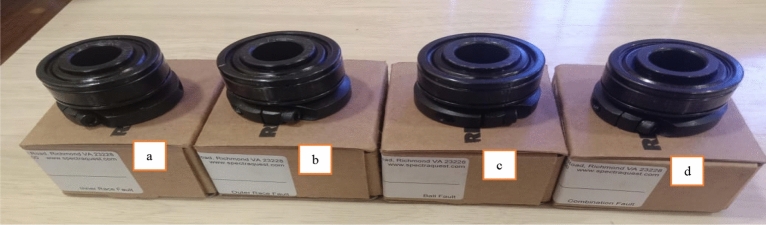
Test bearing with (**a**) Inner Race Fault, (**b**) Outer Race Fault, (**c**) Ball fault, and (**d**) combined faults bearing.

The test rig consisted of dual bearing housing designed to provide support for the drive shaft. The drive shaft was connected to the 220 V, 1/3–1/4 hp DC motor. A collection of deep-groove ball bearings (MB ER-10 K) with a 0.98-inch bore diameter, a 2.05-inch outer diameter, and a 1.49-inch pitch diameter was employed for the purpose of vibration analysis and monitoring. The bearings exhibited a configuration consisting of eight spherical balls, each ball measuring 0.34 inch in diameter. Table 1 presents a comprehensive list of various bearing defects considered in this experimentation. The specific test bearings utilized in the study are visually depicted in Fig. 2. The bearings underwent the creation of individual faults, including ball defects, outer race defects, inner race defects, and combined defects. Fault size is created by grinding with a diamond tip cutter such as Dremel 7134 and the fault size verified using the microscopic measurement.

**Table 1:** Details of bearing fault size.

Type of bearing	Deep groove ball bearing
Condition of the Bearing	Fault size
Healthy bearing (no fault bearing)	–
Inner race fault bearing	1.2 mm wide, 0.5 mm deep, 1.5 mm span
Outer race fault bearing	1.2 mm wide, 0.5 mm deep, 1.5 mm span
Ball fault bearing	Two 1.2 mm dia. dimples at 90 degrees on one ball
Combined fault bearing	Above faults are induced

**Fig. 3:**
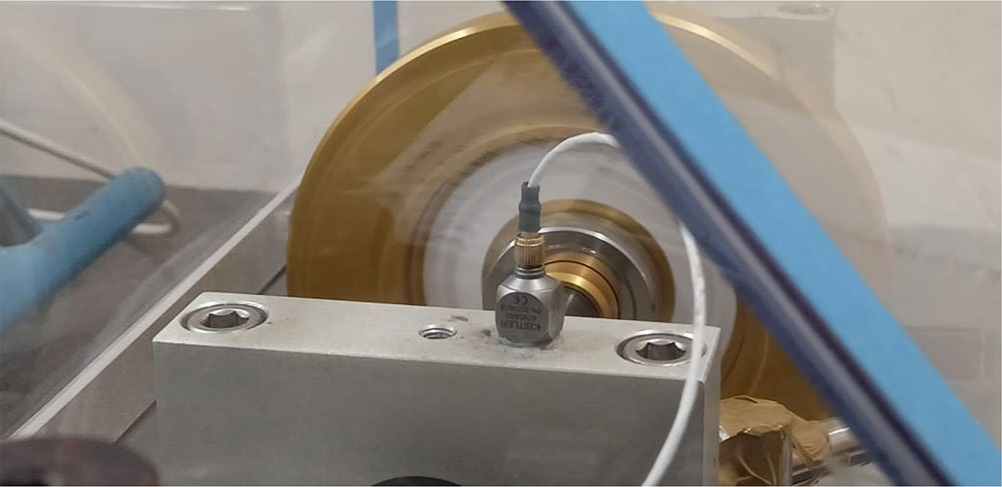
Accelerometer.

The raw vibration signals were obtained by utilizing a Triaxial piezoelectric accelerometer as shown in Fig. 3, specifically, the Kistler 8763A50 model. Accelerometer was securely placed vertically on the bearing housing using wax. Next the various fault conditions were examined to gather the raw vibration data. Atlan DAQ was used to enhance the vibration signals of a various bearing fault condition.

### Dataset description

The sampling frequency of vibration signals plays a critical role in fault diagnosis, as it determines the resolution and the highest detectable frequency components. A higher sampling rate, such as 12,800 Hz, captures high-frequency fault signatures essential for early fault detection but increases data volume and computational costs. In contrast, a lower sampling rate, such as 5120 Hz, still provides sufficient fault-related information but may miss subtle high-frequency components. Proposed study found that the model achieved 95% accuracy at 12,800 Hz and 93.5% at 5120 Hz, indicating a slight performance improvement with a higher sampling rate. According to industry standards like ISO 13,373–2, the sampling rate should be at least 3–5 times the maximum fault frequency to ensure accurate detection. For general rotating machinery, a sampling rate of 10–20 kHz is often recommended to capture critical fault harmonics, while lower rates (2–5 kHz) may suffice for low-speed applications if fault frequencies remain within range. The optimal selection depends on machine speed, fault severity, and data acquisition system constraints.

Dataset1 contains 2,232 samples per fault type (11,160 samples in total), and Dataset2 contains 33,972 samples per fault type (169,860 samples in total). In addition, the model’s performance was evaluated using a new dataset, consisting of 2,934 samples per fault type (14,670 samples in total), with each sample containing 500 data points. The evaluation was conducted under varying speeds and load conditions, with additive noise introduced to the raw signals to assess robustness.

Window splitting data augmentation used to the original vibration signal is divided into fixed-size segments of 500 samples. The holdout method used to split the dataset into training and testing in machine learning (ML) and deep learning (DL). In this study, the dataset is divided into 70/30 ratio for training and testing to evaluate the model’s performance.

**Fig. 4:**
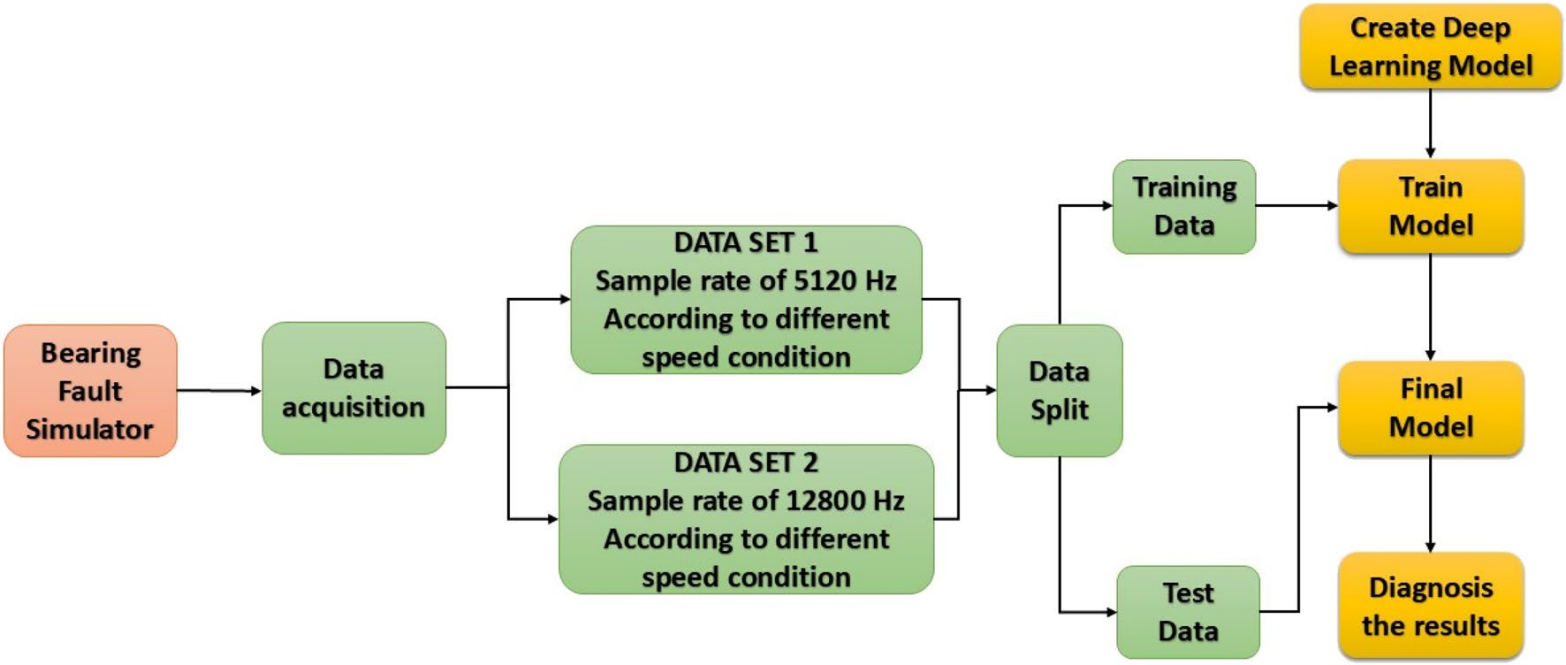
Flow diagram for bearing fault diagnosis.

The study examined the behavior of the bearing at various rotational speeds, including 17.0 Hz, 25.4 Hz, 33.7 Hz, and 42.0 Hz, with sampling rates set at 12,800 Hz for Dataset 1 and 5,120 Hz for Dataset 2. Details of experimental set-up are illustrated in Table 2. MATLAB was utilized to implement the proposed technique for the rolling-element bearing fault diagnostic. The bearing fault diagnosis flow diagram is illustrated in Fig. 4.

**Table 2:** Technical specification for experimental set-up.

Parameter	Details	Parameter	Details
Experimental model	Spectra Quest MFS-PKG5	Motor model	Marathon
Bearing type	MB ER-10 K	H. P	1/3 hp
	Deep groove ball bearing	Max. Speed	4000 rpm
Number of rolling elements	8	Shaft diameter	5/8’ turned, ground, Polished (TGP) Steel
Rolling element diameter	7.939 mm	Accelerometer	Kistler 8763A50—Tri Axial IEPE (Integrated Electronics Piezo-Electric)
Pitch diameter	33.503 mm	Sensitivity, Range	100 mV/g, ± 50 g
Contact angle	0 degree	DAQ system	ATALON: ATA-DAQ 042,451
Inner diameter	19.05 mm	No. of channels, Voltage	4, (24-bit resolution, ± 10 V range)
Outer diameter	47.96 mm	Software used	Dewesoft 7.1

Utilized datasets with sampling rates of 12,800 Hz and 5,120 Hz to evaluate the impact of resolution on fault diagnosis and assess the robustness of proposed C-CNN model. Testing across different resolutions enhances model flexibility and generalization, ensuring adaptability to varying data acquisition settings in real-world applications. While higher sampling rates provide richer fault details, they also increase storage and computational demands, making it essential to assess model performance at lower resolutions for resource-limited environments. Proposed results indicate that the higher sampling rate (12,800 Hz) captured more detailed fault signatures, leading to improved accuracy, whereas the lower sampling rate (5,120 Hz) still provided sufficient information for accurate fault classification, demonstrating the C-CNN’s adaptability. These findings suggest that although higher sampling rates enhance accuracy, the model remains effective at lower resolutions, allowing for flexible deployment based on system constraints and application needs.

## Methodology

A notable approach in DL-based diagnosis involves Customized Convolutional Neural Networks (C-CNNs), tailored specifically for analyzing vibration signals from machinery. C-CNNs are proficient at learning hierarchical representations of data, making them suitable for extracting intricate features that characterize different kinds of bearing faults, such as inner race faults, roller defects, no fault (healthy), outer race damage, and combined faults.

**Fig. 5:**
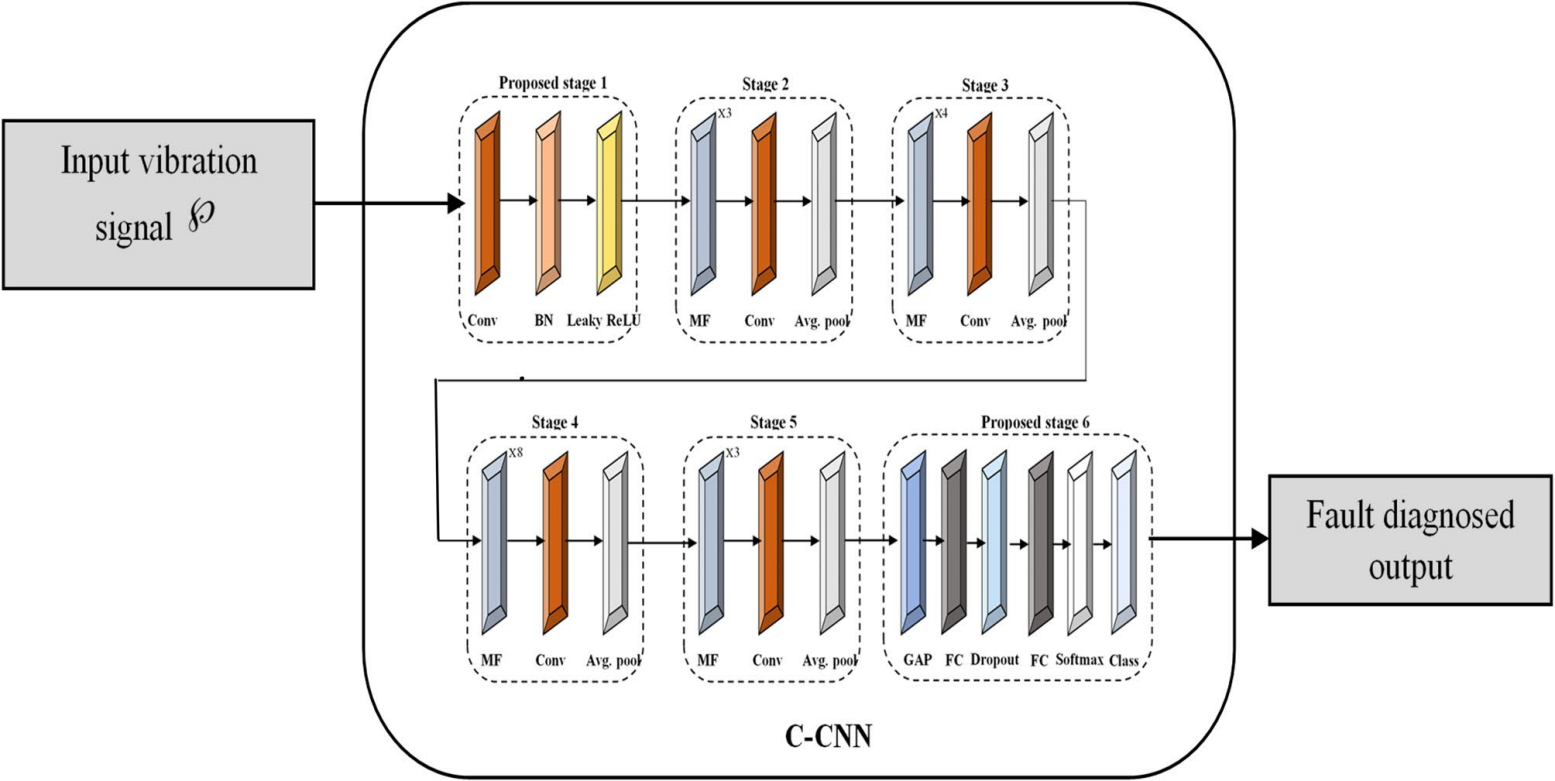
Architecture of proposed work.

In this work, two vibration signals were sampled at 12,800 Hz and 5120 Hz as inputs to C-CNN designed for diagnosing faults in rolling element bearings, as shown in Fig. 5. The C-CNN architecture processed these high-frequency vibration signals through convolutional layers that extracted specific features related to fault types, followed by pooling and dense layers for better identification of characteristic frequencies associated with bearing defects (such as healthy bearing, inner race, outer race, rolling element fault and combined fault). Thus, the proposed approach minimized diagnostic errors and achieved more accurate fault diagnosis, thereby enhancing the reliability as well as the effectiveness of bearing fault detection in industrial settings.

### C-CNN model

**Fig. 6:**
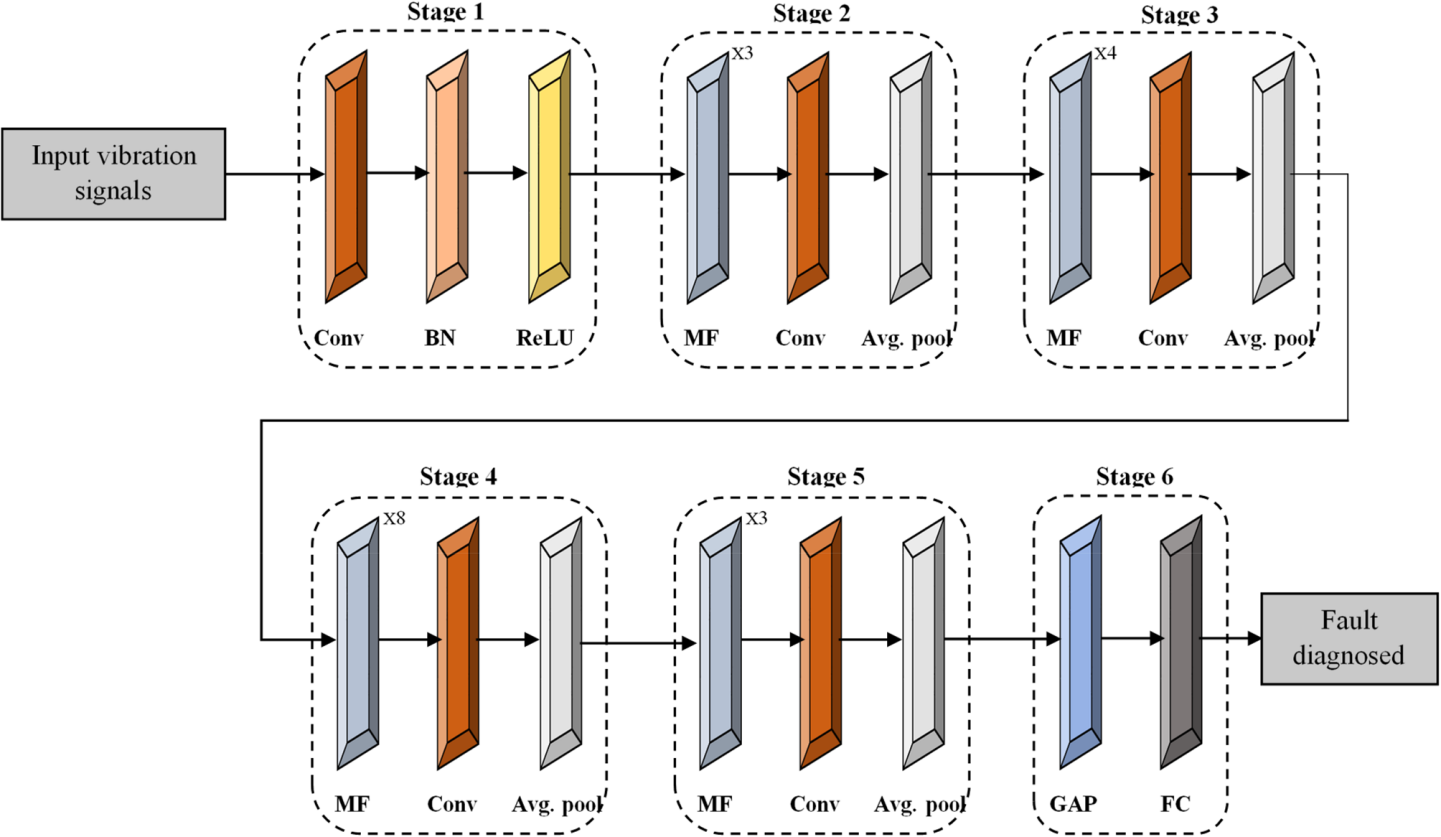
Conventional CNN structure.

Convolutional Neural Networks (CNNs)^26^ have revolutionized various fields of machine learning, particularly in signal processing tasks. The C-CNN is the extension of CNN structure. The standard form of CNN structure consists of several stages, each comprising specific operations and layers designed to process high-frequency vibration signals for fault diagnosis in REBs. As illustrated in Fig. 6, the standard form of structure begins with an input layer that receives temporal sequences of vibration signals and the model progresses through several stages designed to extract and refine features crucial for fault detection.

**Stage 1** initiates with a 3 × 3 convolutional filter to capture local patterns, followed by batch normalization for stabilization and ReLU activation to introduce non-linearity. Subsequent stages consist of multiple filter (MF) blocks, where each block integrates convolutional layers with varying filter sizes to extract features relevant to different fault types. Intermediate 1 × 1 convolutional layers reduce dimensionality while preserving essential features, and average pooling layers down-sample spatial dimensions, enhancing computational efficiency. Finally, the model ends in a global average pooling stage that aggregates feature maps into a condensed representation and quantify probabilities of specific fault categories. This structured approach optimizes the CNN’s capability to discern significant deviations in vibration signals, thereby improving diagnostic accuracy and enabling proactive maintenance strategies in industrial settings.

The primary function of convolutional layers in CNNs is to extract features from the input data, making them essential to the network’s design. In these layers, a convolutional operation is applied to the input data. This operation consists of sliding a filter (also called a kernel) across the input, multiplying corresponding elements of the filter and a small region of the input, and summing these products to create one value in the output feature map. Then the convolutional operation is expressed as in Eq. (1).

1 $$O\left(i,j\right)={\sum }_{p}{\sum }_{q}\wp \left(i+p,j+q\right)w\left(p,q\right)$$

where $\wp$ is the input signal such that $\wp \to \left\{{\wp }_{1},{\wp }_{2}\right\}$; ${\wp }_{1}$ represents input vibration signal with high frequency 12,800 Hz and ${\wp }_{2}$ represents input vibration signal with high frequency 5120 Hz; $\wp \left(i+p,j+q\right)$ indicates elements in the input frequency at position $\left(i+p,j+q\right)$; $O\left(i,j\right)$ is the element of the output feature map at position $\left(i,j\right)$; and $w\left(p,q\right)$ is the convolutional kernel’s weight at position $\left(p,q\right)$.

Pooling layers are important sections of CNNs that minimize the spatial dimensions of feature maps to meet two main objectives: improving computational efficiency and increasing robustness to noise. Moreover, max pooling chooses the highest value within specified regions of the feature map to preserve prominent features, while average pooling evaluates the average value to smooth out minor variations. GAP condenses the whole spatial range of a feature map into a unit vector by averaging across each channel, effectively summarizing learned features and reducing parameters.

Additionally, batch normalization stabilizes and improves CNN performance by normalizing layer outputs during training, ensuring consistent distributions and accelerating convergence. These techniques collectively optimize CNNs for tasks like signal processing, enhancing efficiency and generalization capabilities, as defined as in Eq. (2).

2 $$Bn\left(\wp \right)=\varphi \frac{\wp -\mu }{\sqrt{{\sigma }^{2}+\varepsilon }}+\chi$$

where $\mu$ denotes mean of input signal, $\wp$; ${\sigma }^{2}$ denotes variance of $\wp$; $\varepsilon$ stands for small constant established to avoid division by zero; and $\chi$, $\varphi$ represent shifting parameters and learnable scaling parameters, respectively.

Batch normalization operates by standardizing the activations within hidden layers, thereby mitigating the risk of activations becoming excessively large or small. This normalization process serves to stabilize the network and mitigate issues such as overfitting and instability during training. Moreover, the ReLU activation function^26^ is expressed as in Eq. (3).

3 $$O\left(\wp \right)=\mathit{max}\left(0,\wp \right)$$

where $O$ represents output and $\wp$ represents input to the activation function. Through non-linear transformations, the ReLU function is fundamental for the network to capture complex patterns in the data. By concatenating filters, outputs from different convolutional layers are merged into a single feature map, which improves the CNN’s ability to capture diverse features. Each convolutional kernel is aimed at detecting different input patterns and features. FC layers play a key role in carrying out the final classification tasks. These layers establish connections between all neurons in one layer and every neuron in the following layer, enabling the network to model complex relationships between inputs and outputs.

**Fig. 7:**
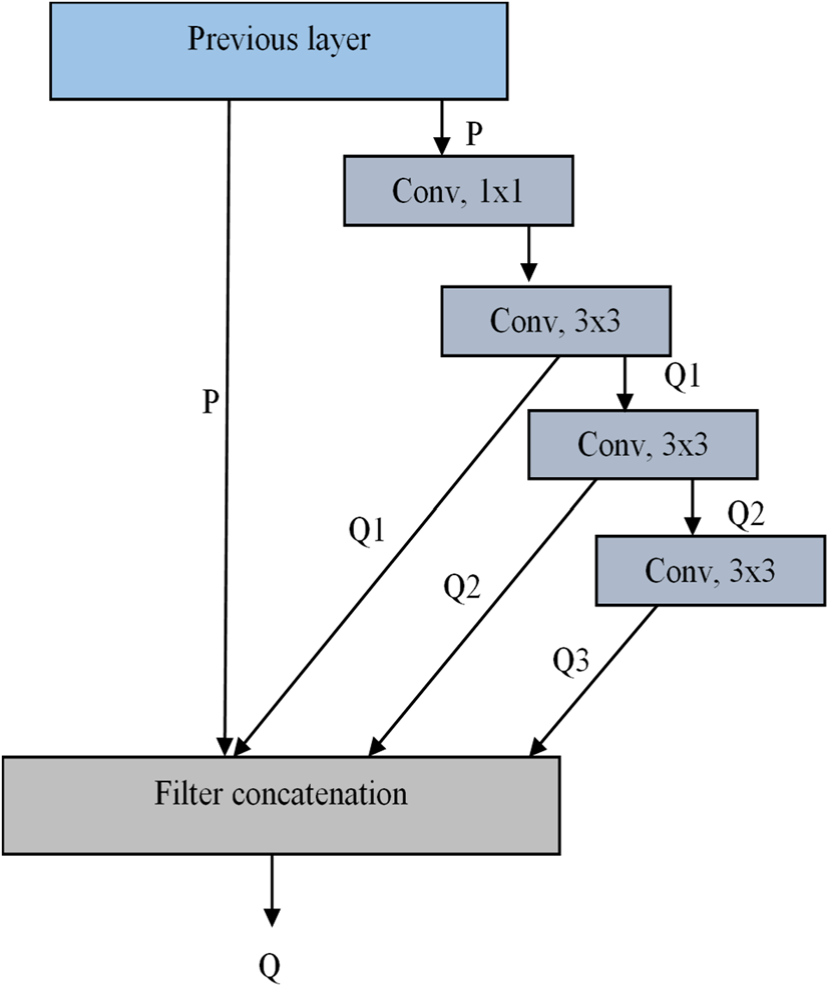
Structure of MF-block.

The stages 2–5 have MF blocks that employ a strategic sequence of convolutions to optimize feature extraction in neural networks, as shown in Fig. 7. The Multi-Feature (MF) block introduces a new way of extracting features efficiently. Unlike traditional multi-scale architectures that use multiple parallel branches, the MF block has a simple yet effective structure with a 1 × 1 convolution followed by three 3 × 3 convolutions. The 1 × 1 convolution helps reduce the number of features while keeping important details, making the model more efficient. The three 3 × 3 convolutions gradually refine the extracted features, capturing deeper patterns. A key difference in the MF block is how it combines the outputs of all these convolution layers along with the output of the previous layer, ensuring that no important information is lost. This method improves feature learning without adding extra complexity, unlike traditional architectures such as Inception or DenseNet. The MF block strikes a balance between performance and efficiency, making it useful for applications that require detailed feature extraction in field of bearing fault classification. The Table 3 provide the details of the MF Block layers and their parameters.

**Table 3:** Detailed structure of the MF block.

Layer	Parameter
*Convolution*	1 × 1 convolutions with stride [1 1] and padding ‘same’
*ReLU*	ReLU
*Convolution*	3 × 3 convolutions with stride [1 1] and padding ‘same’
*ReLU*	ReLU
*Convolution*	3 × 3 convolutions with stride [1 1] and padding ‘same’
*ReLU*	ReLU
*Convolution*	3 × 3 convolutions with stride [1 1] and padding ‘same’
*ReLU*	ReLU
*Depth concatenation*	Depth concatenation of 4 inputs

### Unique contribution of the MF block

- Combines Hierarchical Feature Learning (progressive depth-wise feature extraction) with input reinforcement (concatenating the original input for better information retention).
- Computationally lightweight while still achieving multi-scale feature representation.
- Designed for better spatial feature extraction, making it particularly useful for bearing fault classification tasks requiring fine-grained feature representation.

Unlike residual structures, the MF block operates independently as in Eq. (4–6), focusing on efficient parameter utilization and diverse feature extraction.

4 $${Q}_{1}=con{v}_{3\times 3}\left(con{v}_{1\times 1}\left(P\right)\right)$$

5 $${Q}_{2}=con{v}_{3\times 3}\left({Q}_{1}\right)$$

6 $${Q}_{3}=con{v}_{3\times 3}\left({Q}_{2}\right)$$

Subsequently, these three extracted features are concatenated directly with the initial input, resulting in a unique combined feature map as in Eq. (7).

7 $$Q=concatenate\left(P,{Q}_{1},{Q}_{2},{Q}_{3}\right)$$

Additionally, the CCE is used as the loss function at the output layer, which can lead to numerical instability and sensitive to unfair datasets, where the samples count in each class is not the same. To avoid this, BCE^31^ is introduced in C-CNN architecture.

The BCE loss function is the combination of CCE and CCE as expressed as in Eq. (8–10). Here, $-y\mathit{log}\left(\widehat{y}\right)$ represents CCE.

8 $$L=-\left(\delta y\mathit{log}\left(\widehat{y}\right)+\left(1-\delta \right)\left(1-y\right)\mathit{log}\left(1-\widehat{y}\right)\right)+\left(-y\mathit{log}\left(\widehat{y}\right)\right)$$

9 $$=-\delta y\mathit{log}\left(\widehat{y}\right)-\left(1-\delta \right)\left(1-y\right)\mathit{log}\left(1-\widehat{y}\right)-y\mathit{log}\left(\widehat{y}\right)$$

10 $$L=-\left[\left(1+\delta \right)y\mathit{log}\left(\widehat{y}\right)+\left(1-\delta \right)\left(1-y\right)\mathit{log}\left(1-\widehat{y}\right)\right]$$

where $y$ is the true label and $\widehat{y}$ is the predicted label.

Thus, the proposed BCE addresses the class imbalanced issue through modifying the standard CCE loss. It assigns higher weights to the loss computed for minority classes, making the model pay more attention to correctly identifying them.

**Stage 6:** The architecture involves of a GAP layer and a fully connected (FC) layer, which together make the final classification output. The GAP layer consolidates spatial data from the feature_maps and transforms it into a fixed-dimensional vector. The FC layer subsequently maps this vector onto the given number of output classes, which helps the network build its final decision.

### The proposed C-CNN structure modifies the layout of Stage 1 and Stage 6 that are discussed here

**Fig. 8:**
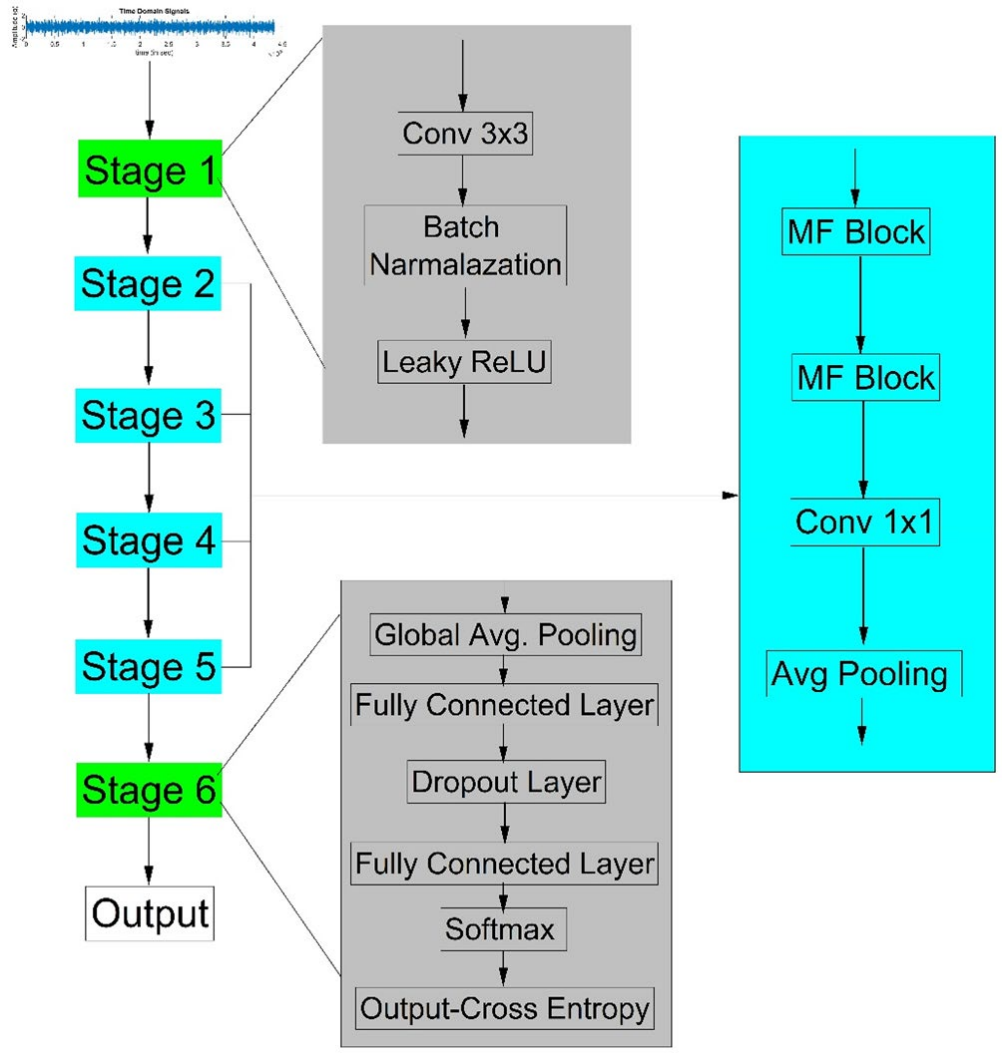
Structure of C-CNN model.

**Stage 1:** The stage 1 comprises convolutional layer, BN and Leaky ReLU. Instead of ReLU activation function Leaky ReLU function is adopted, as depicted in Fig. 8. The Leaky ReLU activation function is a very adaptable and efficient technique that has greatly transformed the field of deep learning and the function given in the Eq. (11). Leaky ReLU addresses the issue of dying ReLU by adding a slight positive slope for inputs with negative values. This modification improves the efficiency and reliability of the training process. The capacity to sustain a low gradient for inputs that are negative guarantees the feasibility of back-propagation, even in demanding situations.

11 $$R(z)=\left\{{ }_{az z\le 0}^{z z>0}\right\}$$

Due to its simplicity and efficacy, Leaky ReLU has gained popularity among academics and practitioners alike, allowing them to construct neural networks that are more precise and resilient. This tool’s advantages have made it a valuable asset in a deep-learning arsenal, propelling advancements in the area. Then is applied a 3 × 3 convolutional filter to capture local patterns and normalizes the output to stabilize and accelerate training and then is applied Leaky ReLU activation function. This function mitigates the dying ReLU problem by permitting a small, non-zero gradient when the input value is negative. It helps maintain a more consistent gradient flow and improve the learning dynamics.

**Stage 6:** This stage comprises six layers including “global average pooling layer, FC Layer, dropout layer, FC layer, Softmax layer and classification layer”. At the beginning of the process, a Global Average Pooling layer is used to minimize the spatial dimensions of feature maps into a single representation by averaging the values for each channel. This streamlined feature vector is then passed through FC layers, where each neuron connects to every neuron in the prior layer, facilitating the learning of complex relationships between extracted features and output classes.

A Dropout layer is utilized to prevent overfitting, randomly deactivating a fraction of neurons during training. Subsequently, another FC layer further refines the features, preparing them for the final stage. The Softmax layer follows, converting the FC layer outputs into probabilities, ensuring that the sum of probabilities across all classes equals one for accurate classification. Lastly, the Classification layer assigns the final class label based on the highest probability score from the Softmax layer, thereby completing the classification process effectively in C-CNN architectures designed for diverse machine learning tasks. Thus, the proposed C-CNN architecture obtains improved generalization by adopting a dropout layer. This helps to prevent overfitting, which means the model is likely to generalize better on unseen data. Also, it has achieved enhanced learning capacity and regularization because, the dropout layer acts as a regularizer making the model more robust and loss likely to overfit the training data. The softmax layer provides a clear probabilistic interpretation of the models’ predictions, which can be useful for computing the models’ confidence in its prediction. This structured approach enhances the C-CNN’s ability to detect significant deviations in vibration signals, thereby improving diagnostic accuracy and facilitating proactive maintenance strategies in industrial settings. The details of the proposed mode layers and their parameters are presented in Table 4.

**Table 4:** C_CNN architecture and parameter details.

Stages	Layer	Parameter	Activation function
Stage_1	Convolution 1	Kernel 32 3 × 3 convolutions with stride [1 1] and padding ‘same’	Leaky ReLU
	Batch narmalization	–	–
Stage_2	MF Block × 3	104	–
	Convolution2	104 1 × 1 convolutions with stride [1 1] and padding ‘same’	Leaky ReLU
	Average pooling	2 × 2 average pooling with stride [2 2] and padding [0 0 0 0]	–
Stage_3	MF Block × 4	200	–
	Convolution3	200 1 × 1 convolutions with stride [1 1] and padding ‘same’	Leaky ReLU
	Average pooling	2 × 2 average pooling with stride [2 2] and padding [0 0 0 0]	–
Stage_4	MF Block × 8	392	–
	Convolution4	392 1 × 1 convolutions with stride [1 1] and padding ‘same’	Leaky ReLU
	Average pooling	2 × 2 average pooling with stride [2 2] and padding [0 0 0 0]	–
Stage_5	MF Block × 3	464	–
	Convolution5	104 1 × 1 convolutions with stride [1 1] and padding ‘same’	Leaky ReLU
	Average pooling	2 × 2 average pooling with stride [2 2] and padding [0 0 0 0]	–
Stage_6	Global average pooling	464	–
	Fully connected	5	–
	Dropout	0.5%	–
	Fully connected	5	Softmax
	Classification output	BCE	–

The C-CNN model utilizes vibration signals from rolling element bearings (REBs) as input data, leveraging their rich information for effective fault detection. To enhance model performance, batch normalization is applied to normalize the input of each layer, maintaining a mean output close to zero and a standard deviation close to one. This technique speed up training and improves network stability. Additionally, the Multi-Feature (MF) block is incorporated to extract diverse features from vibration signals, enabling a more accurate fault diagnosis. To prevent overfitting and enhance generalization, dropout is employed by randomly ignoring selected neurons during training, ensuring robust model performance across different datasets.

### Hyperparameter tuning

Hyperparameter tuning in neural networks refers to the process of selecting the optimal settings for hyperparameters that affect the functionality and performance of the neural network model. Before the training process begins, essential non-learning parameters known as hyperparameters are defined. These parameters include the learning rate, the number of layers, activation functions, number of neurons per layer, and regularization parameters among others. It is crucial to meticulously adjust these hyperparameters, as they significantly influence the model’s efficiency to achieve the best results during training.

In this research, the hyperparameter tuning process for the C-CNN with MF block was conducted using the Adam optimizer over 200 trials to achieve optimal performance in bearing fault classification. The following key hyperparameters were explored:

**Total No. of Layer:** 179.

**Activation Function**: softmax.

**Epochs:** 200.

**Learning rate:** Fixed at 0.001, as it provided stable convergence without causing vanishing or exploding gradients.

**Batch size:** Set to 50, balancing computational efficiency and model performance.

**Dropout rate:** Experimented within a range of 0.1 to 0.5 to prevent overfitting while maintaining sufficient model complexity.

The Adam optimizer with cosine decay was selected based on its ability to adaptively adjust learning rates, improving convergence speed and generalization. The final hyperparameter values were chosen based on the lowest validation loss, ensuring that the model achieved optimal accuracy while preventing overfitting to the training data.

### Role of Adam optimization in diagnosing fault bearings

Adam optimization (Adaptive Moment Estimation) is a prominent stochastic gradient descent optimization technique used for training deep learning models. It is an adaptive learning rate optimization approach that merges the advantages of AdaGrad and RMSProp. Adam optimization plays a crucial role in deep learning-based fault-bearing diagnosis, as it enhances model training efficiency and generalization. Diagnosing bearing faults is often a challenging task due to the complex and non-linear behaviour of the vibration signals generated by the bearings. Utilizing Adam optimization in training a deep learning model assists the model to learn complicated aspects of vibration signals and improves its accuracy in diagnosing faulty bearings.

Adam’s variable learning rate, compared with fixed learning rate methods, enables the model to converge more quickly and reliably. This is particularly important for diagnosing bearing problems, as accurate and timely diagnosis can help in reducing machinery breakdowns and lowering maintenance costs. Additionally, Adam optimization prevents overfitting by adjusting the learning rate for each parameter during training, leading to improved generalization and performance on new and unseen data. This is vital in diagnosing bearing problems, as the model must generalize well to different types of bearings and fault conditions.

Finally, this research explores various machine learning performance for the classification of bearing faults. The various methods discussed include Long Short-Term Memory (LSTM), Support Vector Machine (SVM), Random Forest (RF), Deep Belief Network (DBN), Multi-Scale Convolutional Neural Network (MSCNN), Artificial Neural Network (ANN), K-Nearest Neighbors (KNN), SqueezeNet, ResNet and Convolutional Neural Network (CNN). Furthermore, the training process involves using the Adam optimizer, setting the number of epochs as 200, and the batch size as 50. The trained models are evaluated on test data to determine their classification performance and results are compared with the proposed C-CNN. This comparative analysis will be helpful for the identification of the most effective model for specific datasets.

## Results and discussion

The proposed C-CNN model demonstrates high accuracy in bearing fault classification, enabling industries to detect potential issues at an early stage. This early diagnosis plays a crucial role in reducing downtime by preventing unexpected equipment failures, thereby improving overall productivity. Additionally, the model facilitates timely maintenance, allowing industries to address faults before they escalate into severe failures that could lead to costly machinery shutdowns. By optimizing maintenance schedules based on accurate fault detection, the model contributes to significant cost savings, reducing unnecessary repairs and extending equipment lifespan. Furthermore, its ability to identify faults proactively supports preventive maintenance strategies, minimizing unplanned downtime and ensuring continuous operation. These advantages highlight the model’s effectiveness in real-world industrial applications, making it a valuable tool for predictive maintenance and operational efficiency.

The integration of the Multi-Feature (MF) block significantly improved the model’s ability to extract diverse characteristics from vibration signals, such as frequency components, amplitude variations, and temporal patterns. This enhanced feature representation contributed to more precise fault classification, as evidenced by the model’s improved accuracy. Unlike residual connections, the MF block’s concatenation strategy effectively preserved critical information without introducing excessive computational overhead, allowing for better generalization across different fault conditions. Furthermore, the use of the Balanced Cross-Entropy (BCE) loss function played a crucial role in optimizing classification performance. By incorporating class weights (δ = 0.7 for minority classes), BCE loss reduced false negatives, ensuring that fault classes with fewer samples were accurately detected. During training, minimizing BCE loss led to stable convergence and better model robustness, demonstrating its effectiveness in handling class imbalance. These findings highlight the combined impact of the MF block and BCE loss function in enhancing fault diagnosis accuracy and overall model performance.

The choice of activation function plays a crucial role in the learning capabilities of neural networks. In Stage 1 of proposed C-CNN model, we replaced the standard ReLU activation function with Leaky ReLU to address key limitations in feature learning. One major advantage of this choice is mitigating the dying ReLU problem, where neurons become inactive due to zero gradients for negative inputs. Leaky ReLU resolves this by allowing a small, non-zero gradient, ensuring neurons remain active and continue contributing to learning. This modification also enhances training efficiency by preventing dead neurons, leading to a more stable and faster convergence process. Furthermore, the slight negative slope in Leaky ReLU supports enhanced feature learning, enabling the model to extract valuable information even from negative input variations, which is particularly useful for fault diagnosis. In this study, implementing Leaky ReLU in Stage 1 improved model performance, leading to higher accuracy and better generalization, especially when handling complex vibration signal data.

A detailed examination was carried out to assess the effectiveness of a C-CNN approach in comparison with conventional approaches for diagnosing faults in Rolling Element Bearings. Evaluation metrics such as “Sensitivity, False Discovery Rate (FDR), Negative Predictive Value (NPV), Specificity, F-measure, Precision, False Positive Rate (FPR), Matthews Correlation Coefficient (MCC), and Accuracy” were utilized in the assessment. The C-CNN method was compared with the state-of-the-art methods such as ResNet, CNN and MSCNN, along with the conventional classifier models including RF, DBN, KNN, ANN, SVM, LSTM, and SqueezeNet.

1. K-Nearest Neighbour (KNN)

The K-Nearest Neighbour (KNN) method serves as a powerful tool in the realm of machine learning, particularly for classifying faults in bearings. To classify a new signal, the algorithm determines the distance from this new point to every data point in the training dataset. Next, the classification of the new point is based on the predominant category among these nearest points. In this study the function as fitcknn was used and was processed in MATLAB.

**Fig. 9:**
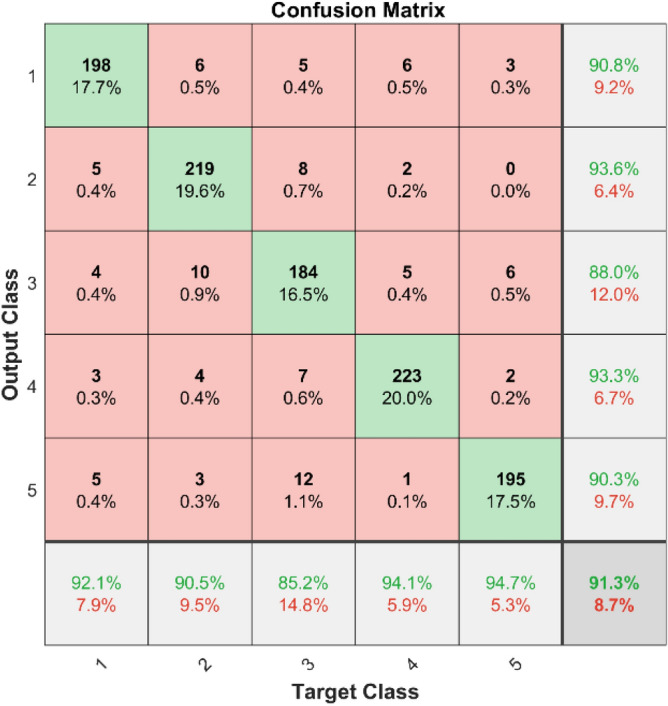
Confusion Matrix of KNN for dataset 1.

In Fig. 9 shows the confusion matrix illustrates the effectiveness of the KNN classifier on the first dataset. The diagonal components indicate the correctly classified instances for each class, while the off-diagonal components show the misclassified instances.

**Hyperparameter:** In this study the k = 3 was used, Euclidean distance with equal weight to train the model.

**Fig. 10:**
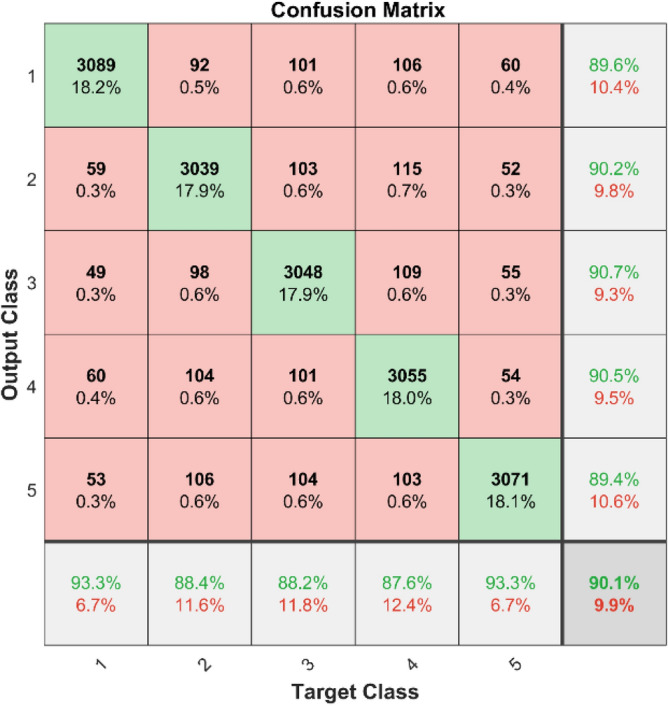
Confusion matrix of KNN for dataset 2.

**Fig. 11:**
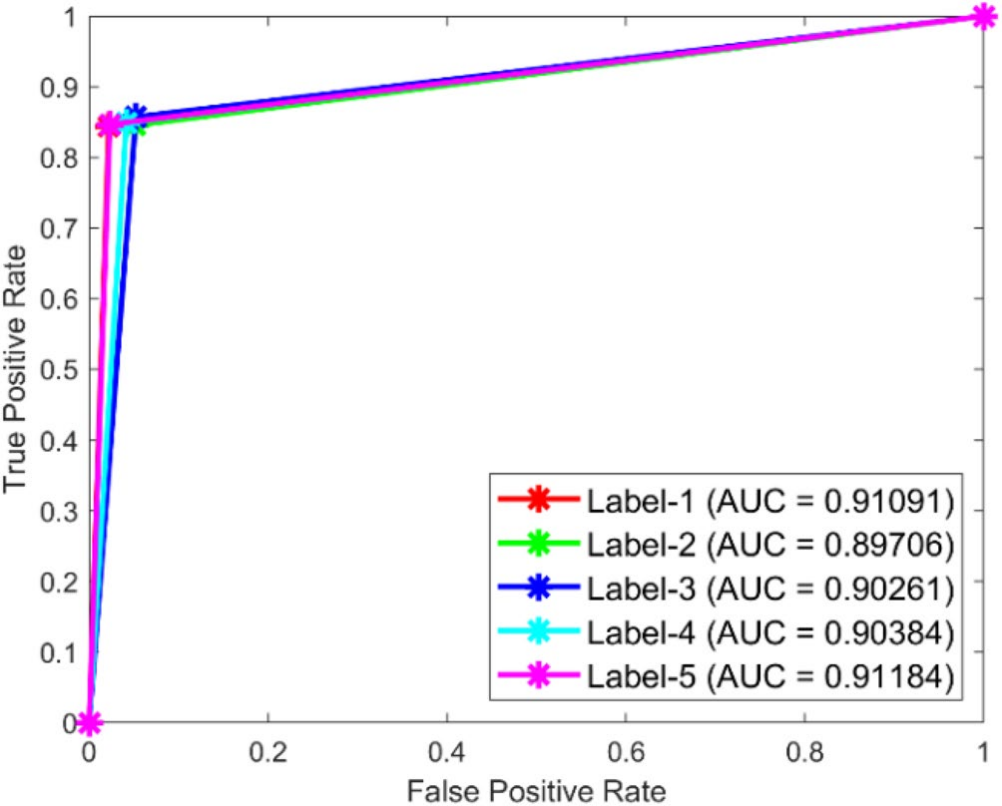
ROC of KNN for dataset 1.

**Fig. 12:**
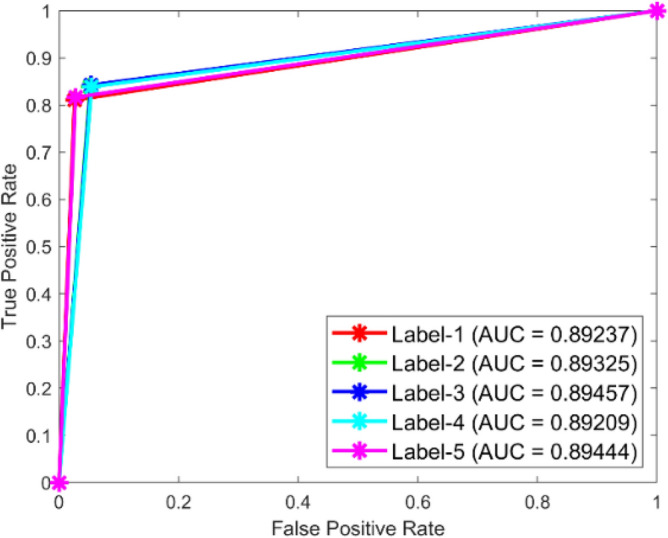
ROC of KNN for dataset 2.

**Performance**: Figs. 9 and 10 show the confusion matrix for the dataset 1 and dataset 2 respectively. In dataset 1, the kNN achieved accuracies of 90.8%, 93.6%, 88.0%, 93.3%, and 90.3% for classes 1 to 5. Figure 11 shows the ROC curve plots the False Positive Rate (FPR) against the True Positive Rate (TPR) for each class in the first dataset. The Area Under the Curve (AUC) values are provided for each class, with label-1 having an AUC of 0.91091, indicating best performance of the classifier when compared with the other labels. In dataset 2, the kNN has achieved accuracies of 89.6%, 90.2%, 90.7%, 90.5%, and 89.4% for classes 1 to 5. Figure 12 shows the ROC curves for the second dataset. The AUC values for the different classes are obtained. The label-1 achieving an AUC of 0.89237, demonstrates the classifier’s ability to accurately identify this class.

The confusion matrices and ROC curves collectively provide a comprehensive evaluation of the kNN classifier’s performance on two different datasets, highlighting its accuracy and ability to differentiate between various bearing fault conditions. These visualizations are crucial for assessing the classifier’s effectiveness and identifying areas for improvement.

2. Artificial Neural Network (ANN)

Artificial Neural Networks (ANNs) work like a human brain, and have the capability for identifying faults in bearings. Layers of interconnected nodes form an artificial neural network, with an input layer gathering features, hidden layers performing computational tasks, and an output layer delivering classifications. The backpropagation method was used to train the artificial neural network. It modified the network’s weights to reduce the difference between the predicted outcomes and the actual results. Activation functions that exhibit non-linear characteristics, such as ReLU, enabled the model to classify the faults effectively. Moreover, cross-entropy Loss functions were used to quantify the prediction error, and Adam optimizer was used to modify the weights to reduce the error. Metrics such as accuracy, precision, recall, and F1-score assessed the performance of the model.

**Hyperparameter:** Adam optimizer, 200 epochs, and the mini-batch size- 50 were used to train the model.

**Fig. 13:**
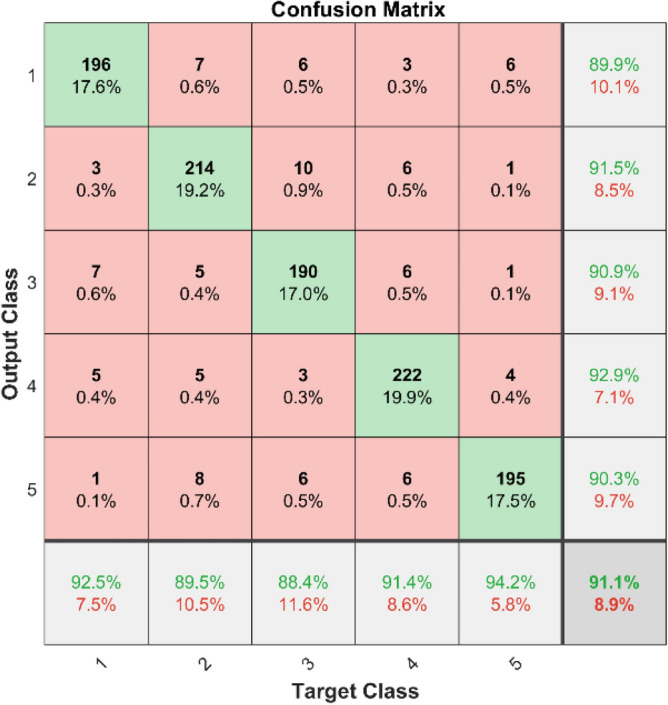
Confusion matrix of ANN for dataset 1.

**Fig. 14:**
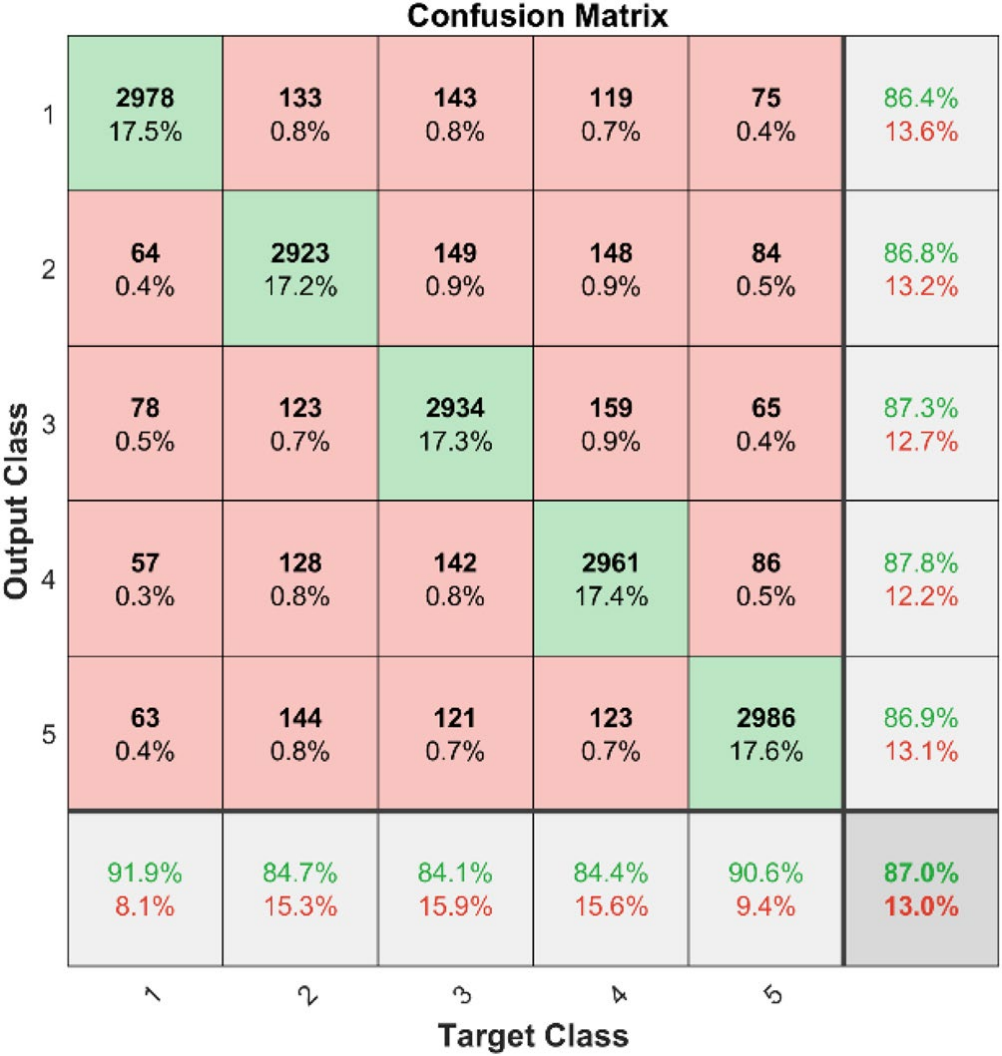
Confusion Matrix of ANN for dataset 2.

**Performance:** The results of ANN for bearing fault diagnosis are depicted in the confusion matrices and ROC curves for two datasets. Figures 13 and 14 show confusion matrix for dataset 1 and dataset 2, respectively. These matrices illustrate the classification performance of the ANN. The diagonal elements show correct classification and the off-diagonal elements show incorrect predictions for each class.

**Fig. 15:**
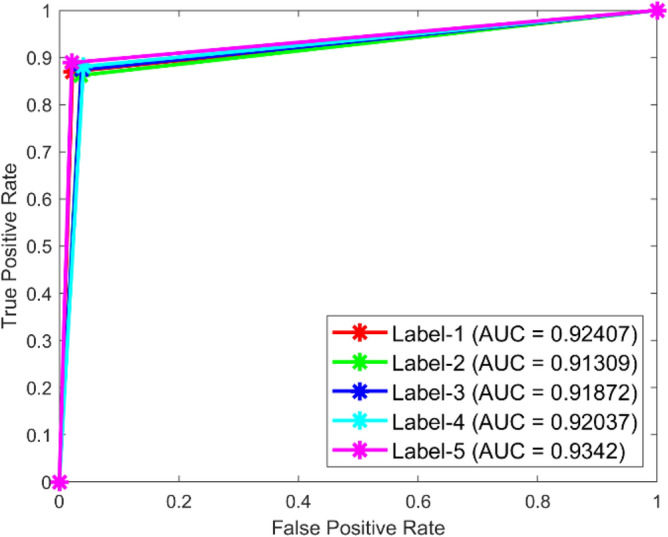
ROC of ANN for dataset 1.

**Fig. 16:**
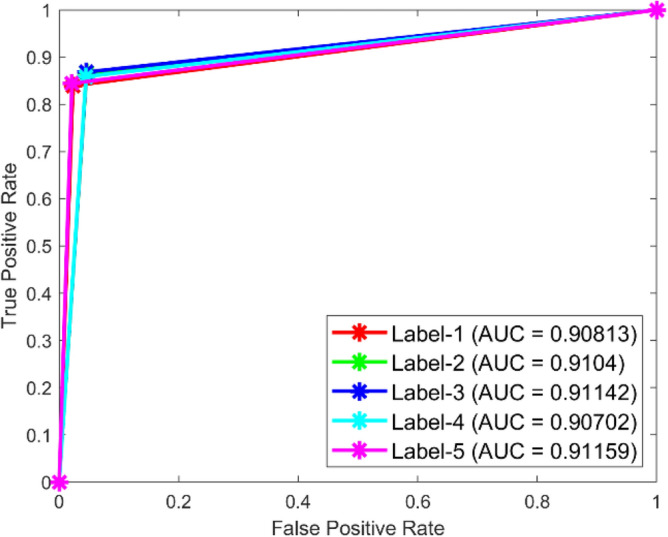
ROC of ANN for dataset 2.

In dataset 1, the ANN achieved accuracies of 89.9%, 91.5%, 90.9%, 92.9%, and 90.3% for classes 1 to 5. The ROC curves for dataset 1, shown in Fig. 15, indicate AUC values of 0.92407, 0.91309, 0.91872, 0.92037, and 0.9342 for labels 1 to 5, respectively. For dataset 2, the ANN recorded accuracies of 84.6%, 86.8%, 87.3%, 87.8%, and 86.9% for the respective classes, as shown in Fig. 14. The corresponding ROC curves in Fig. 16 show AUC values of 0.90813, 0.9104, 0.91142, 0.90702, and 0.91159 for labels 1 to 5. These results underscore the robustness of the ANN model, with high accuracy and significant AUC values across both datasets, affirming its capability to effectively diagnose bearing faults.

3. Support Vector Machines (SVM)

Support Vector Machines (SVM) represent a robust and adaptable approach in ML, particularly effective for classification challenges, such as identifying faults in bearings. The SVM identifies the best hyperplane to distinguish between various classes within the feature space. This hyperplane optimally separates the nearest points of distinct classes, referred to as support vectors. SVM can handle both linear and non-linear classification challenges by using different types of kernel functions, such as linear, polynomial, and radial basis function (RBF) kernels. These functions convert the input data into higher-dimensional spaces, enhancing the effectiveness of linear separation. Throughout the training process, the SVM fine-tunes this hyperplane to ensure maximum classification precision. The evaluation of the model’s performance involves metrics such as accuracy, precision, recall, and F1-score.

**Hyperparameter:** To train the SVM model, certain hyperparameters such as the kernel function and kernel scale are to be provided. In this study, we employed a linear kernel with automatic scaling and a box constraint set to 1, and processed in MATLAB.

**Fig. 17:**
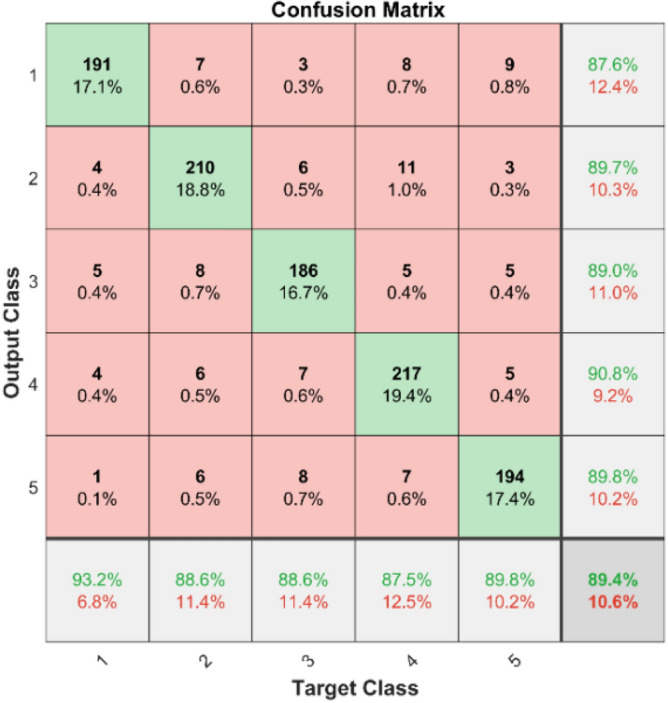
Confusion matrix of SVM for dataset 1.

**Fig. 18:**
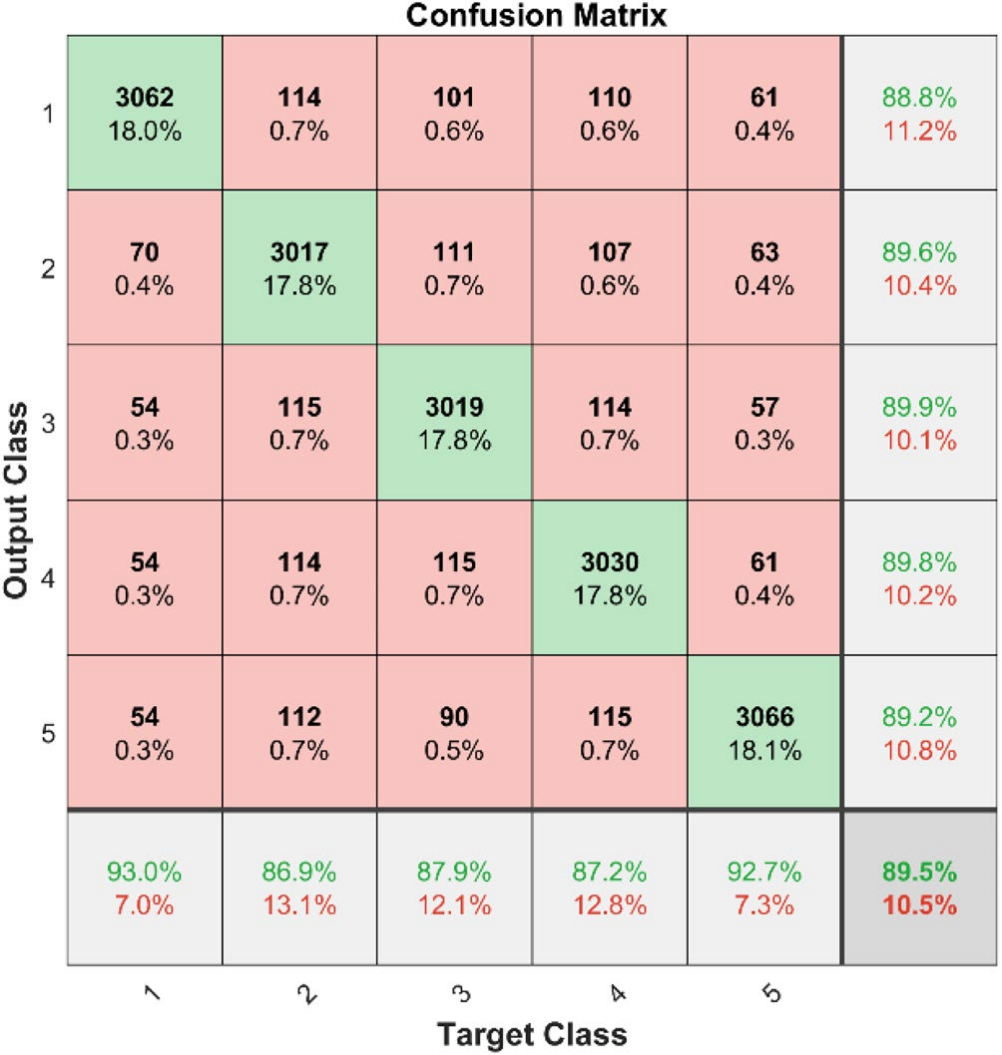
Confusion matrix of SVM for dataset 2.

**Performance:** The confusion matrix and ROC curves for the two datasets illustrate the performance of SVM in detecting bearing defects. Figures 17 and 18 display confusion matrices for dataset 1 and dataset 2, respectively. These matrices show how well the SVM can classify bearing faults. For each class, the diagonal elements show correct predictions and the off-diagonal elements show incorrect ones.

**Fig. 19:**
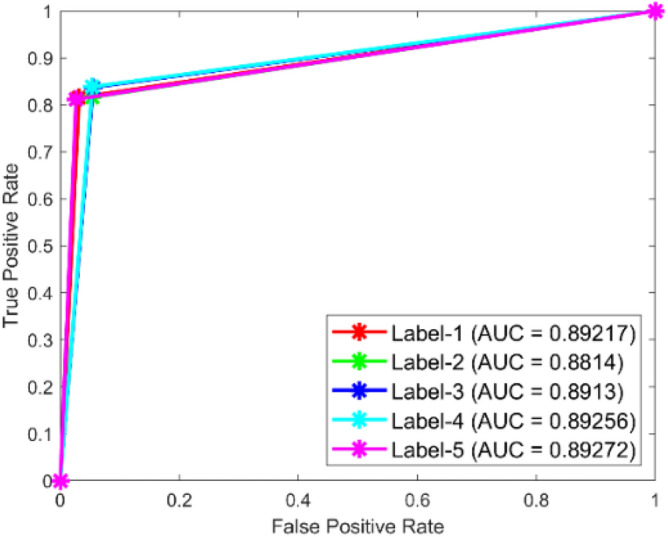
ROC of SVM for dataset 1.

**Fig. 20:**
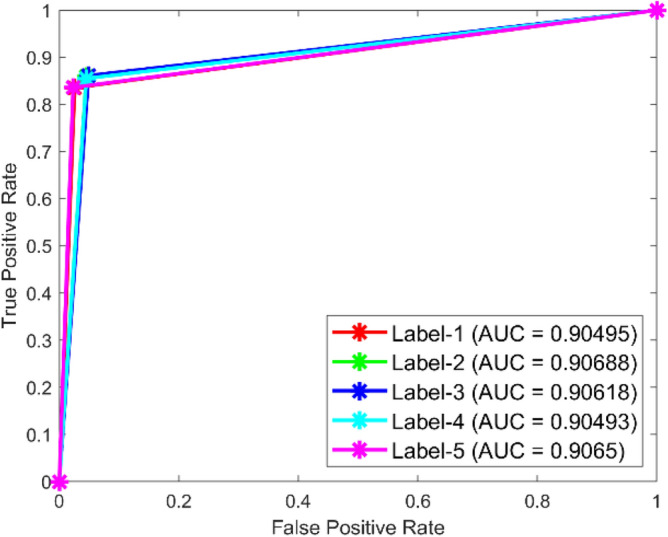
ROC of SVM for dataset 2.

In dataset 1, the SVM attained accuracies of 87.6%, 89.7%, 89.0%, 90.8%, and 89.8% for classes 1 through 5. Figure 19 shows the ROC curves for dataset 1 for labels 1 through 5; they show AUC values of 0.89217, 0.8814, 0.8913, 0.89256, and 0.89272. In dataset 2, the model achieved accuracies of 88.8%, 89.6%, 89.9%, 89.8%, and 89.2% for the appropriate classes, as seen in Fig. 18. The ROC curves depicted in Fig. 20 provide AUC values of 0.90495, 0.90688, 0.90618, 0.90493, and 0.9065 for labels 1 through 5.

4. Random forest (RF)

Random Forest (RF) is a powerful ensemble learning technique that performs exceptionally well in classification tasks, particularly in identifying faults in bearings. It functions by building several decision trees throughout the training process and providing the class that represents the most common classification among the individual trees. This approach utilizes the strengthened decision trees to enhance prediction precision and effectively manage overfitting. Each tree in the forest is constructed from a random selection of the training data and features, fostering diversity among the trees and strengthening the models. Furthermore, the model’s performance is evaluated using various metrics such as accuracy, precision, recall, and F1-score. Through the aggregation of predictions from individual trees, Random Forest can proficiently differentiate among various fault conditions by analyzing vibration signals.

**Fig. 21:**
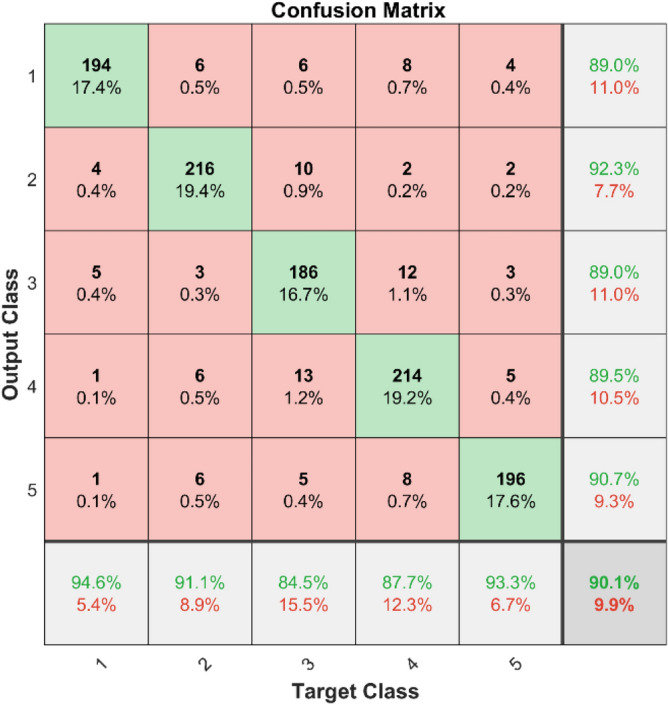
Confusion matrix of RF for dataset 1.

**Fig. 22:**
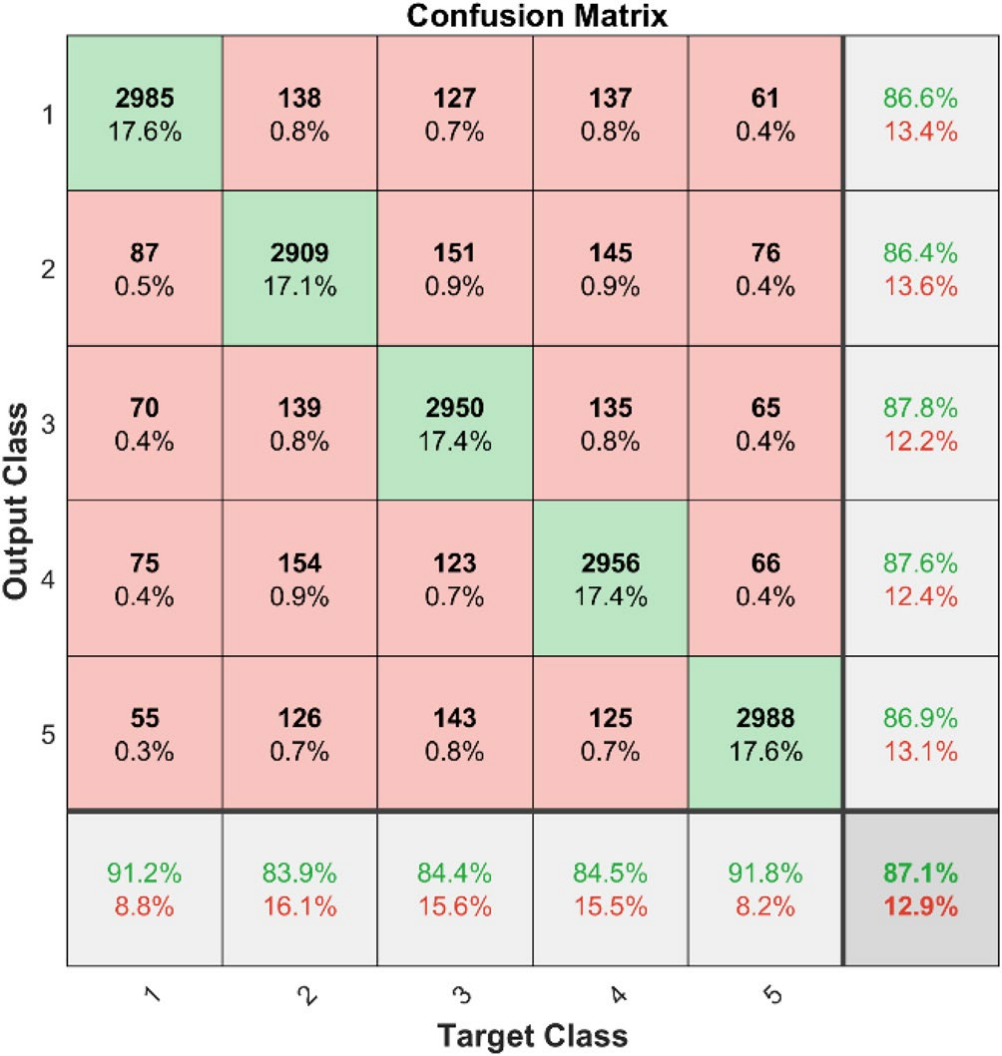
Confusion matrix of RF for dataset 2.

The confusion matrices and ROC curves for two datasets demonstrate the effectiveness of SVM in identifying bearing defects. Figures 21 and 22 display the confusion matrices for dataset 1 and dataset 2, respectively. These matrices show how well the RF can classify things by showing both correct (diagonal elements) and incorrect (off-diagonal elements) predictions for each class.

**Hyperparameter:** To train the Random Forest model, ‘three trees’ was used with a maximum depth of one and three splits at each node.

**Fig. 23:**
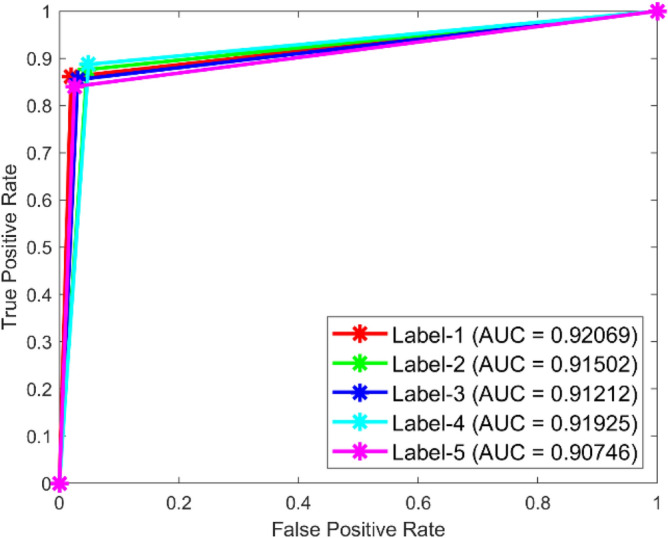
ROC of RF for dataset 1.

**Fig. 24:**
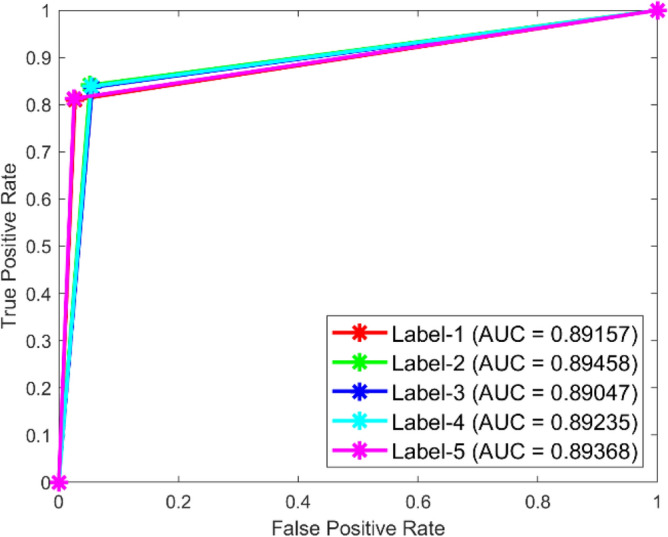
ROC of RF for dataset 2.

**Performance:** In dataset 1, the Random Forest attained accuracies of 89.0%, 92.3%, 89.0%, 89.5%, and 90.7% for classes 1 through 5. Figure 23 shows the ROC curves for dataset 1 for Labels 1 through 5. They have AUC values of 0.92069, 0.91502, 0.91212, 0.91925, and 0.90746. The RF accuracy rates for the different classes in dataset 2 are 86.6%, 86.4%, 87.8%, 87.6%, and 86.9%, as shown in Fig. 22. The ROC curves depicted in Fig. 24 exhibit AUC values of 0.89157, 0.89458, 0.89047, 0.89235, and 0.89368 for labels 1 through 5. The RF model is strong, as shown by the high accuracy and large AUC values it showed in both datasets.

5. Long Short-Term Memory (LSTM)

Long Short-Term Memory (LSTM) networks, subset of Recurrent Neural Network (RNN) predominantly well-suited to time-series data and sequential tasks, such as bearing fault classification. They address traditional RNN shortcomings by utilizing memory cells that retain information over extended periods. These cells use gates to regulate information flow, allowing LSTMs to identify long-term dependencies and patterns in the data.

In the bearing fault classification, the process involves collecting vibration signal data over time, which inherently contains temporal patterns. LSTMs can analyze these sequences by maintaining and updating the state of the memory cells based on the input data, extracting meaningful features that are relevant for fault detection. Performance metrics such as accuracy, precision, recall, and F1-score are used to evaluate the model’s effectiveness.

**Fig. 25:**
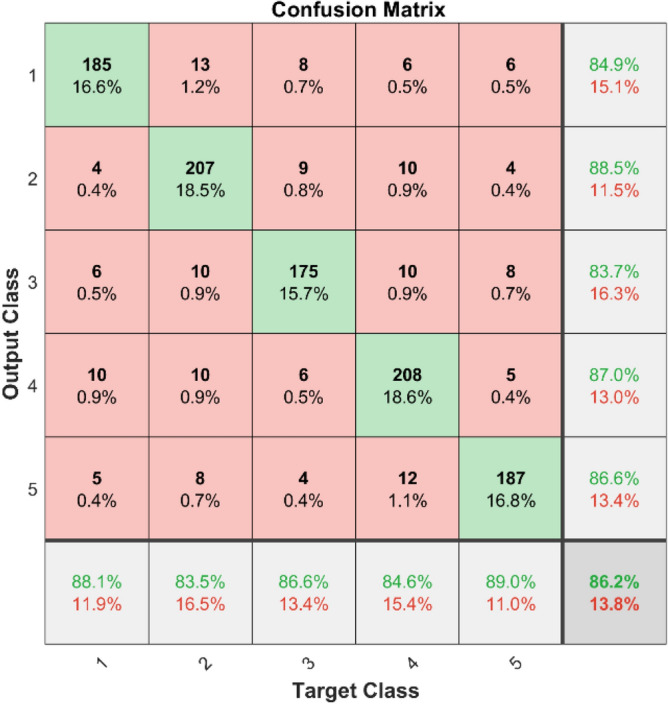
Confusion matrix of LSTM for dataset 1.

**Fig. 26:**
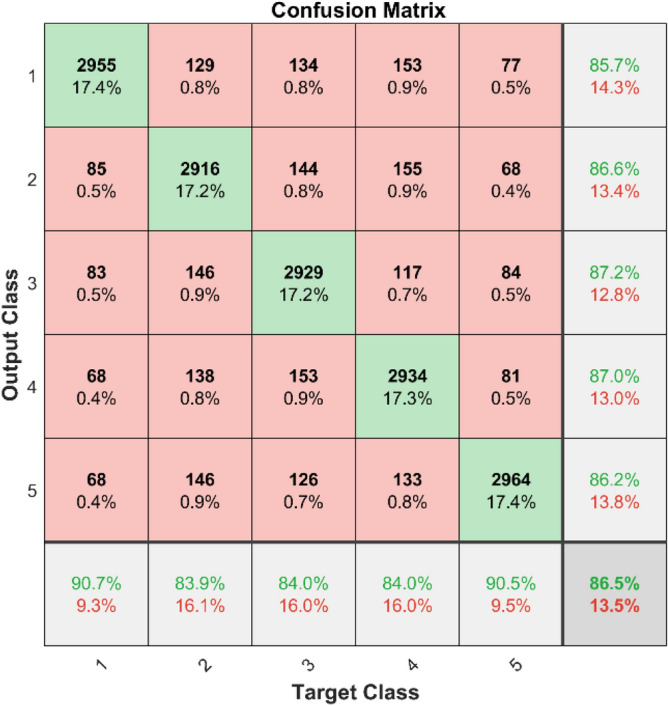
Confusion matrix of LSTM for dataset 2.

The results of using LSTM for bearing fault diagnosis are depicted in the confusion matrices and ROC curves for two datasets. Figures 25 and 26 present confusion matrices for dataset 1 and dataset 2, respectively. These matrices illustrate the classification performance of the LSTM, showing both the correct prediction in diagonal elements and incorrect predictions in the off-diagonal elements for each class. For instance, in dataset 1, the LSTM accurately classifies the majority of the instances, but there are some misclassifications indicated by the off-diagonal elements.

**Hyperparameter:** To train the model, the hyperparameter is set as having epochs 200, mini-batch size is 50, and the LSTM adjusts its weights through backpropagation to minimize the classification error.

**Fig. 27:**
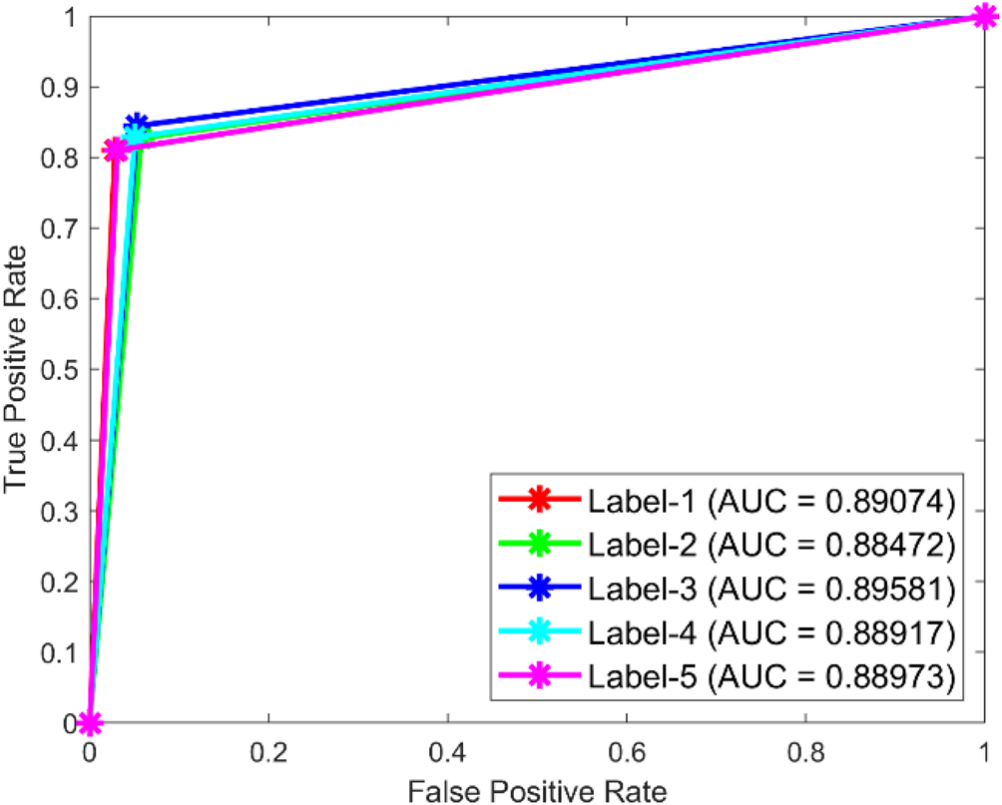
ROC of LSTM for dataset 1.

**Fig. 28:**
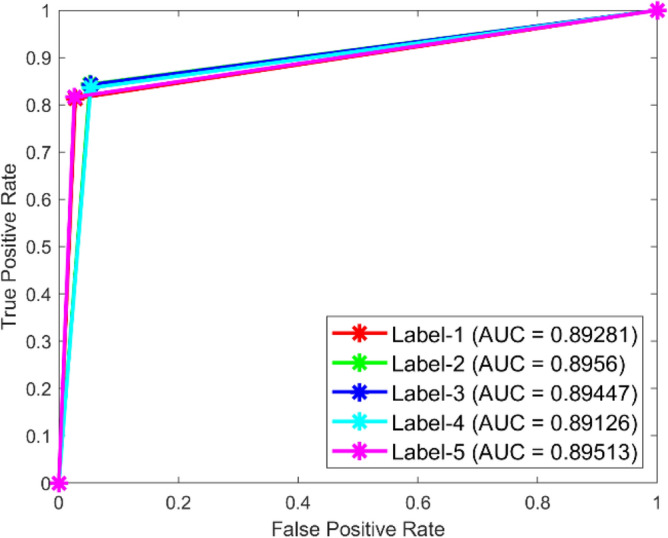
ROC of LSTM for dataset 2.

**Performance:** In dataset 1, the LSTM achieved accuracies of 84.9%, 88.5%, 83.7%, 87.0%, and 86.6% for classes 1 to 5. The ROC curves for dataset 1, shown in Fig. 27, indicate AUC values of 0.89074, 0.88472, 0.89581, 0.88917, and 0.88973 for Labels 1 to 5, respectively. For dataset 2, the LSTM recorded accuracies of 85.7%, 86.6%, 87.2%, 87.0%, and 86.2% for the respective classes, as shown in Fig. 26. The corresponding ROC curves in Fig. 28 present AUC values of 0.89281, 0.8956, 0.89447, 0.89126, and 0.89513 for labels 1 to 5. These results underscore the robustness of the LSTM model, with high accuracy and significant AUC values across both datasets.

6. Deep Belief Network (DBN)

Deep Belief Networks (DBNs) represent a category of deep learning models that perform best in the classification of faults in bearings. Initially, this network is trained in an unsupervised fashion, layer by layer, before refining them through a supervised learning approach. This training helps deep belief networks understand complex, sequence pattern of the input data. This makes them better at tasks that need to deal with high-dimensional, non-linear data, for bearing fault classification.

Restricted Boltzmann Machines (RBM) layer has been used to identify complex patterns within the data, gradually constructing a deep network for bearing faults classification. Further, the DBN’s performance has been measured using various metrics such as accuracy, precision, recall, and F1-score.

**Fig. 29:**
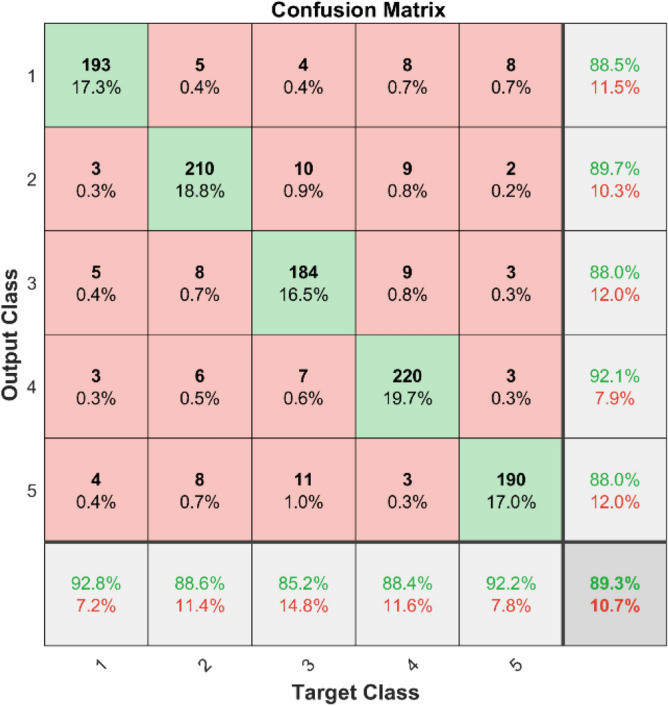
Confusion matrix of DBN for dataset 1.

**Fig. 30:**
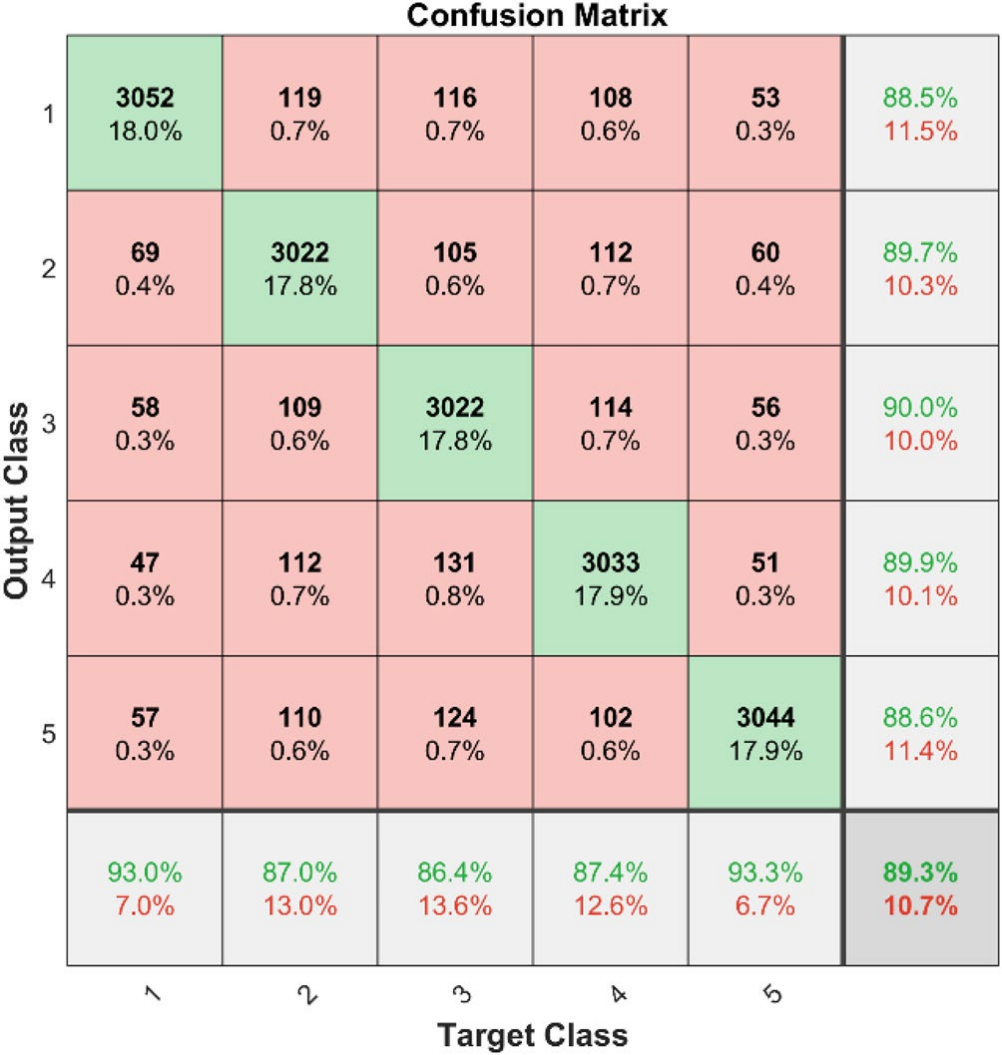
Confusion matrix of DBN for dataset 2.

The confusion matrices and ROC curves for two datasets show the effects of using DBN to find bearing faults. For dataset 1 and dataset 2, the confusion matrices can be seen in Figs. 29 and 30. These matrices show how well the DBN can classify items by displaying both accurate predictions in the diagonal elements and inaccurate predictions in the off-diagonal elements for each class.

**Hyperparameter**: To train the process, the parameter is set such as 200 max iterations, batch size of 50, step ratio of 0.2, dropout rate of 0.1.

**Fig. 31:**
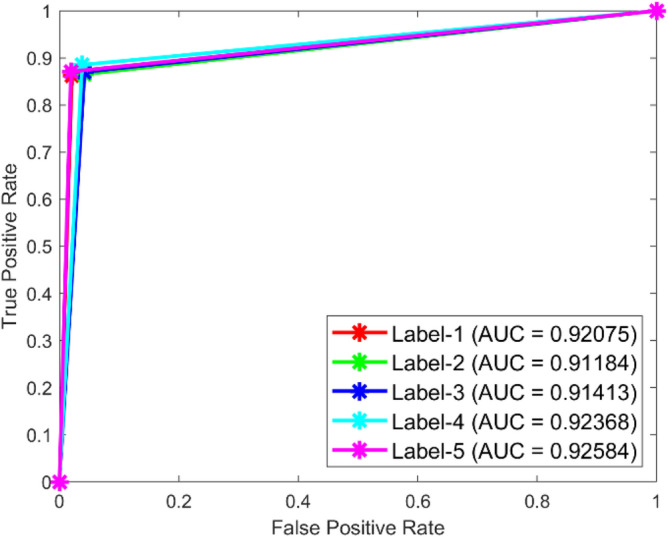
ROC of DBN for dataset 1.

**Fig. 32:**
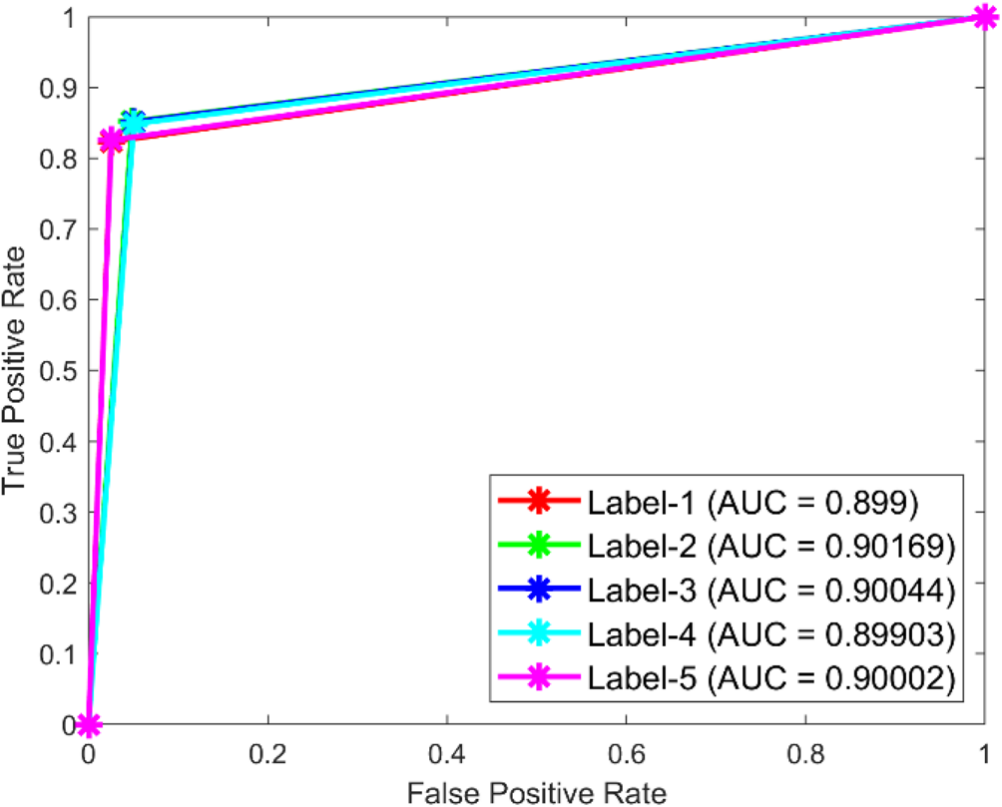
ROC of DBN for dataset 2.

**Performance:** For classes 1 through 5, the DBN got 88.5%, 89.7%, 88.0%, 92.1%, and 88.60% correct in dataset 1. Figure 31 shows the ROC curves for dataset 1 for labels 1 to 5. They show AUC values of 0.92.75, 0.91184, 0.91413, 0.92368, and 0.92584, in that order. In dataset 2, as shown in Fig. 30, the DBN got 88.5%, 89.7%, 90.0%, 89.9%, and 88.6% accuracy for each class. Figure 32 shows the ROC curves that go with labels 1 through 5. The AUC values for these curves are 0.899, 0.90169, 0.90044, 0.89903, and 0.90002. With high accuracy and significant AUC values across both datasets, these results show that the DBN model is very stable and can accurately identify bearing problems.

7. ResNet

ResNet, or Residual Network, is a deep learning architecture specifically designed to overcome the challenges of training very deep neural networks, particularly the vanishing gradient problem. By incorporating residual connections, which allow information to bypass multiple layers, ResNet ensures stable gradient flow and enables effective training of extremely deep models. This architecture is highly effective for bearing fault diagnosis, as it better at extracting hierarchical features from vibration signals. Additionally, ResNet’s robustness to noise makes it well-suited for real-world industrial applications, where vibration signals are often affected by operational fluctuations. The network’s depth also enhances its capacity to generalize across varying loads, speeds, and fault conditions, making it a reliable choice for predictive maintenance. With its strong feature extraction capability, stability, and adaptability, ResNet stands out as a powerful tool for intelligent fault diagnosis and condition monitoring in rotating machinery. The results obtained from the two identical datasets are presented as follows, highlighting the model’s performance across various evaluation metrics.

**Fig. 33:**
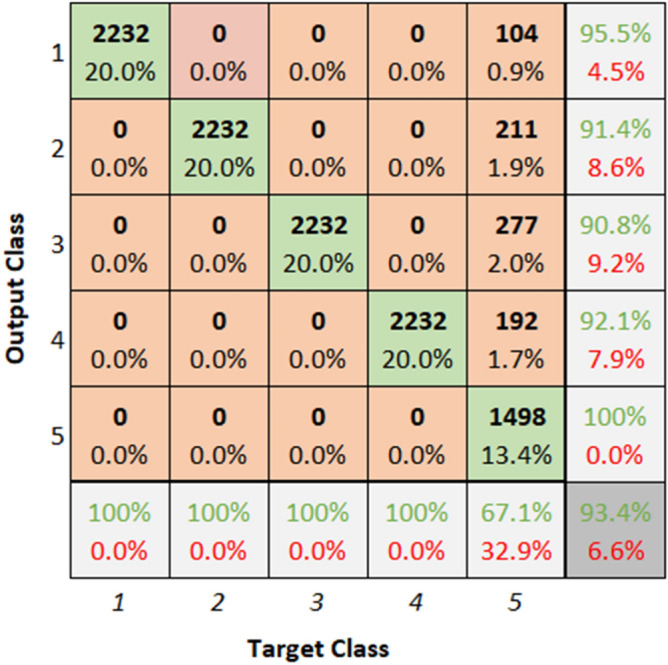
Confusion matrix of ResNet for dataset 1.

**Fig. 34:**
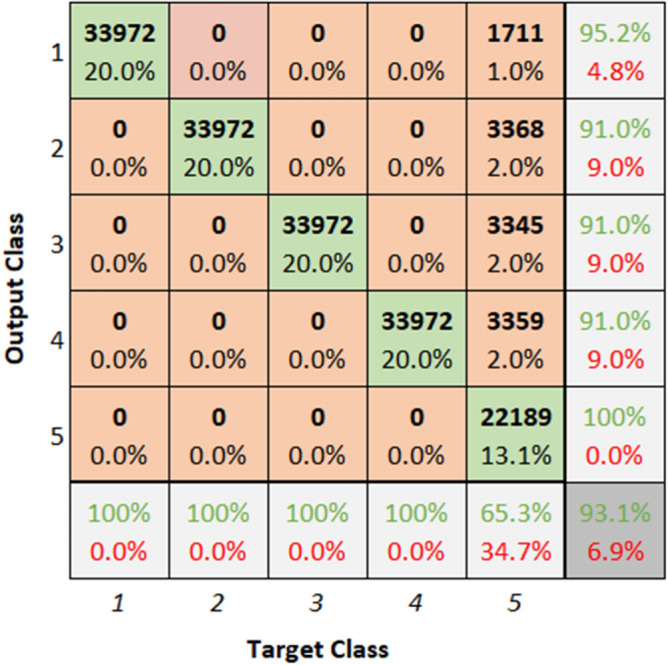
Confusion matrix of ResNet dataset 2.

The confusion matrices and ROC curves illustrate the classification performance of the model for bearing fault diagnosis. Figures 33 and 34 present the confusion matrices for dataset 1 and dataset 2, highlighting the model’s ability to classify different fault categories.

**Hyperparameters:** The following parameter are used to train the model such as Optimizer: Adam, Initial Learning Rate: 0.001, Batch Size: 200, and Epochs: 50.

**Fig. 35:**
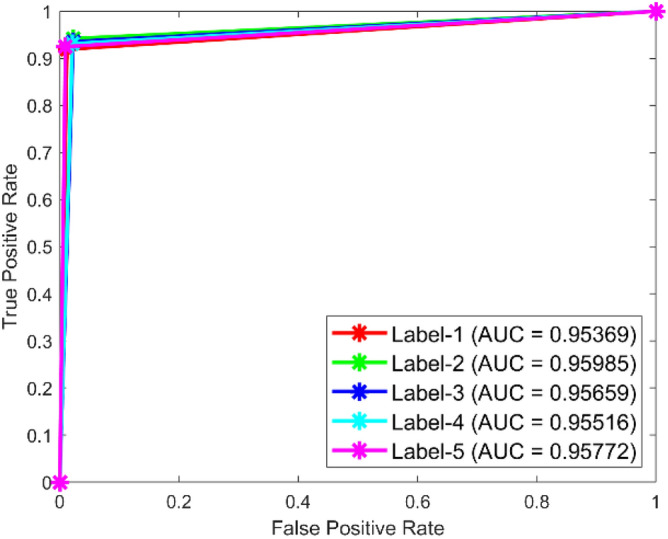
ROC of ResNet for dataset 1.

**Fig. 36:**
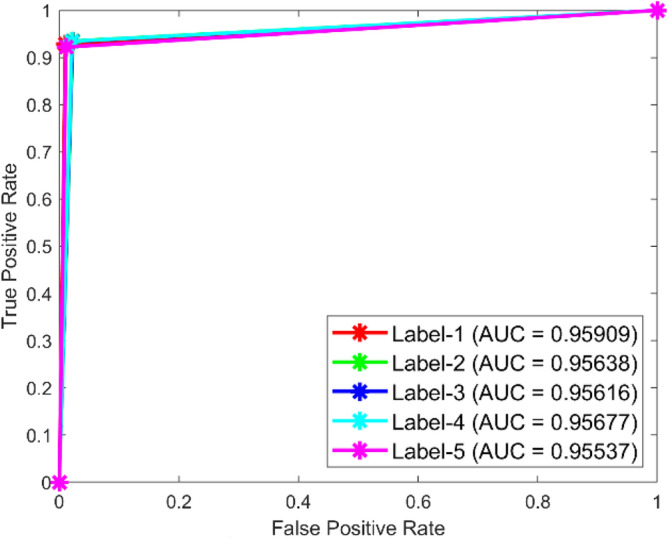
ROC of ResNet for dataset 2.

**Performance:** The classification accuracy for dataset 1 across classes 1 to 5 is 95.5%, 91.4%, 90.8%, 92.1%, and 100%, respectively. Similarly, for dataset 2, the model achieved 95.2%, 91.0%, 91.0%, 91.0%, and 65.3% accuracy for the five classes. The ROC curves in Figs. 35 and 36 further validate the model’s performance, with AUC values of 0.95369, 0.95985, 0.95659, 0.95516, and 0.95772 for dataset 1, and 0.95909, 0.95638, 0.95616, 0.95677, and 0.95537 for dataset 2. These best AUC values confirm the model’s strong ability to differentiate between fault classes.

8. SqueezeNet

SqueezeNet is a small and efficient deep learning model created for image classification, achieving similar accuracy to larger models. This efficiency is achieved through a strategy called "squeeze and expand." The “squeeze” layers minimize the number of input channels by using 1 × 1 convolutions, drastically cutting down the number of parameters. The “expand” layers then increase the complexity and expressiveness of the model by using a mix of 1 × 1 and 3 × 3 convolutions. This design makes SqueezeNet both lightweight and powerful, suitable for deployment on resource-constrained devices without sacrificing performance. In the context of bearing fault classification, SqueezeNet can be trained on vibration signal data to classify different fault condition. By training the model with labeled data, it learns to recognize patterns associated with different bearing conditions. The performance of the model can be evaluated using metrics such as accuracy, precision, recall, and F1-score to ensure reliable fault diagnosis.

**Hyperparameter:** The model is trained using the Adam optimizer over 200 epochs.

**Fig. 37:**
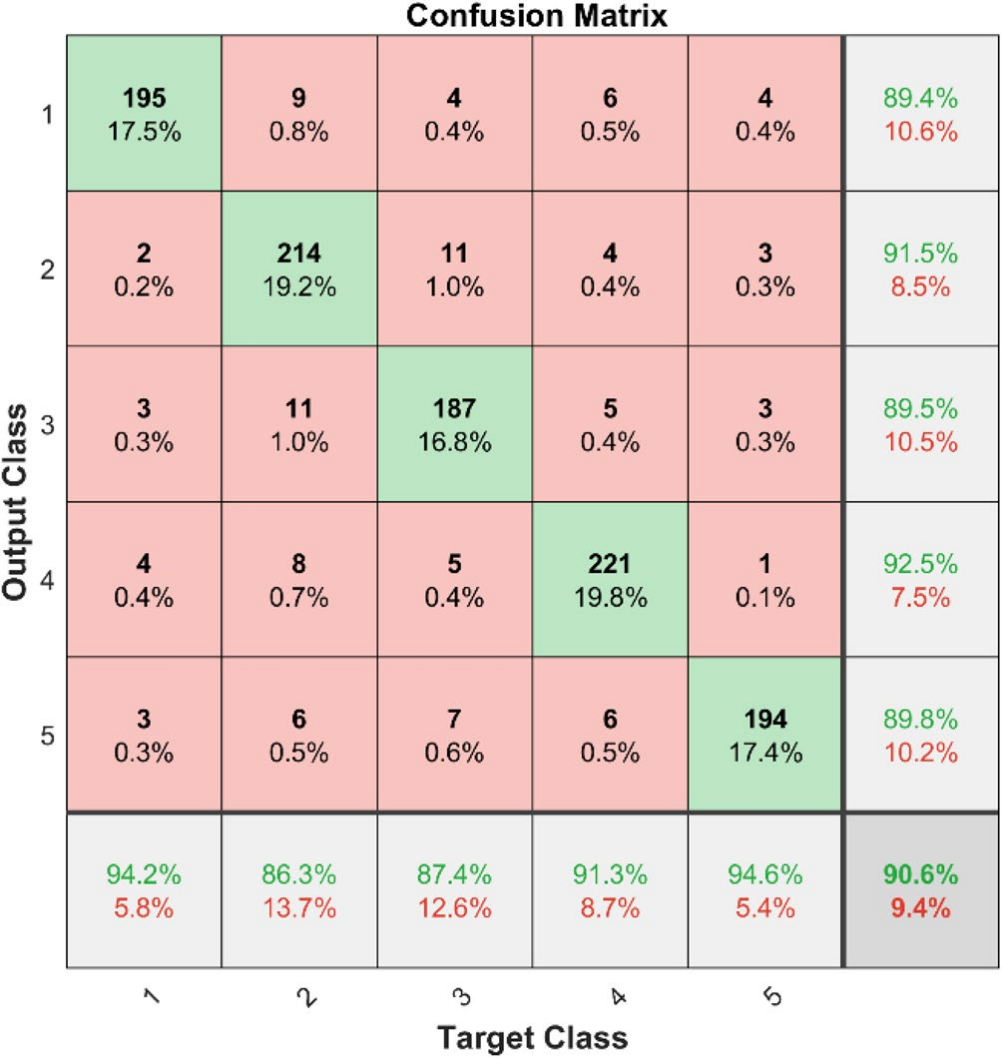
Confusion matrix of SqueezeNet for dataset 1.

**Fig. 38:**
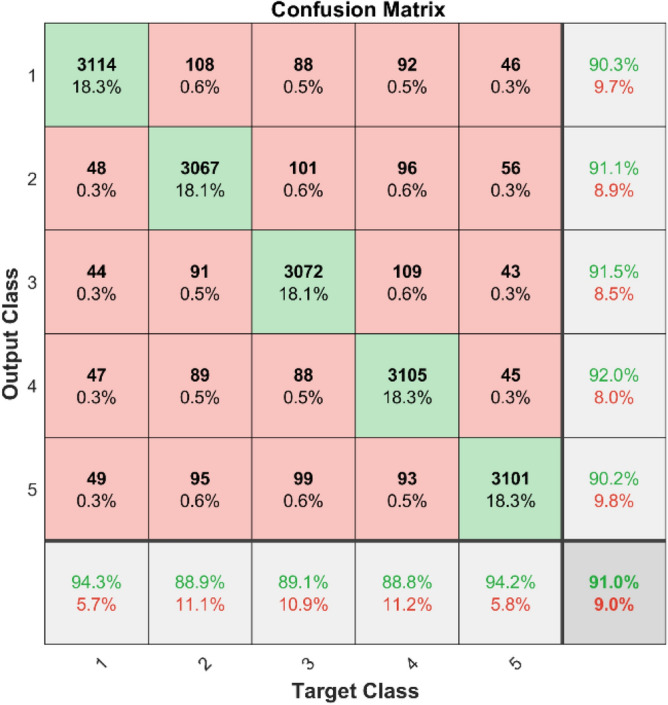
Confusion matrix of SqueezeNet for dataset 2.

**Performance:** The results of using SqueezeNet to find bearing faults are shown by the confusion matrices and ROC curves for two sets of data. Figures 37 and 38 show confusion matrices for datasets 1 and 2, respectively. These matrices show how well the SqueezeNet can classify things, showing both correct (diagonal elements) and incorrect (off-diagonal elements) predictions for each group. For instance, in dataset 1, the SqueezeNet accurately classifies the majority of cases, with the off-diagonal components highlighting certain misclassifications.

**Fig. 39:**
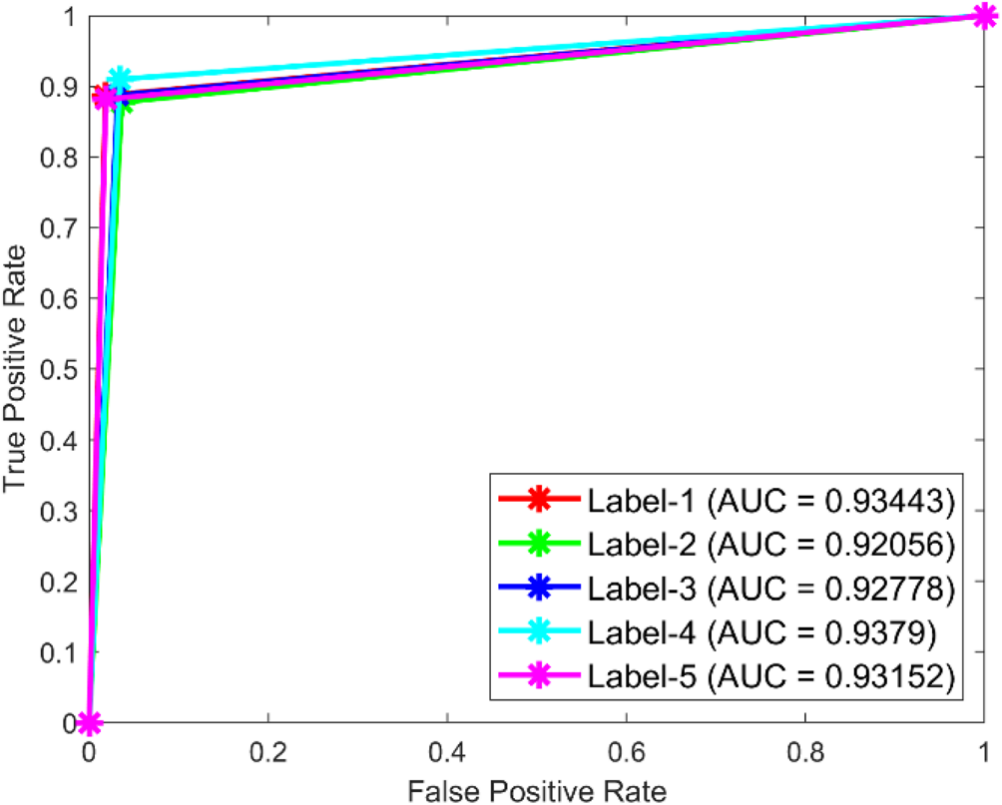
ROC of SqueezeNet for dataset 1.

**Fig. 40:**
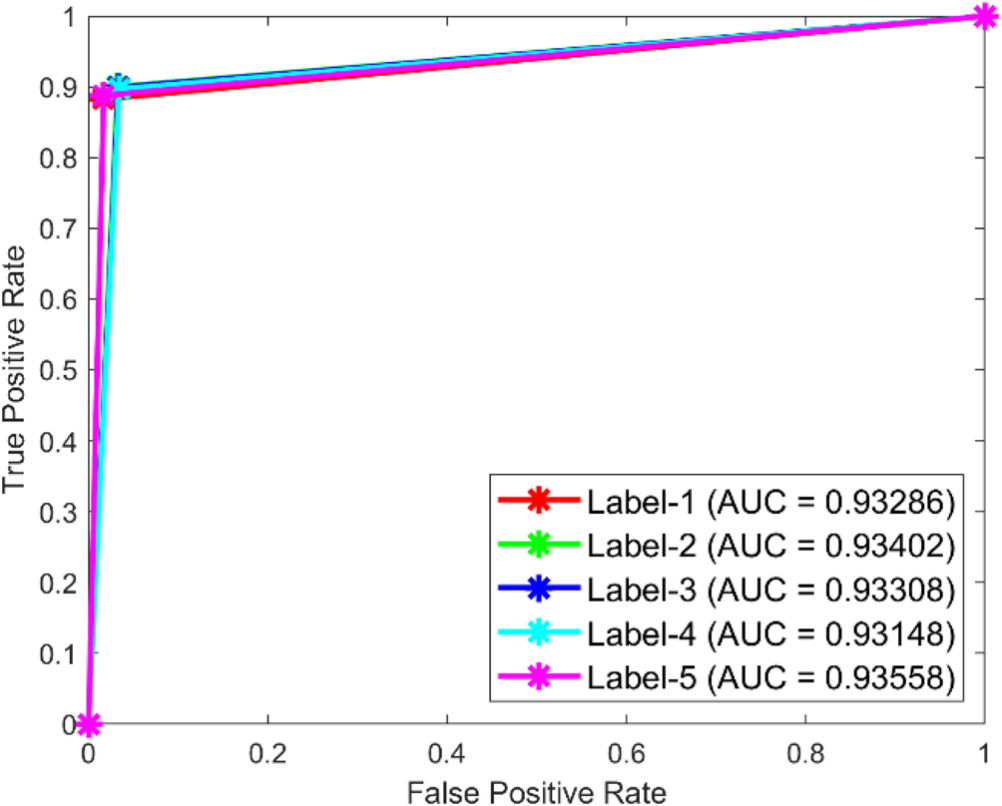
ROC of SqueezeNet for dataset 2.

In dataset 1, the SqueezeNet attained accuracies of 89.4%, 91.5%, 89.5%, 92.5%, and 89.8% for classes 1–5. Figure 39 shows the ROC curves for dataset1 for labels 1–5. The AUC values are 0.93433, 0.92.56, 0.92778, 0.9379, and 0.93152, in that order. In dataset 2, the SqueezeNet got 90.3%, 91.1%, 91.5%, 92.0%, and 90.2% accuracy for the right classes, as seen in Fig. 8. For Labels 1–5, the corresponding ROC curves in Fig. 40 show AUC values of 0.93286, 0.93402, 0.93308, 0.93148, and 0.93558, respectively. With high accuracy and large AUC values across both datasets, these results show how resilient the SqueezeNet model is and confirm its ability to find bearing defects.

9. Multi-scale Convolutional Neural Network (MSCNN)

Multi-scale Convolutional Neural Networks (MSCNN) are a sophisticated deep learning framework designed to handle data at different scales. This makes them very useful for tasks like finding defect with bearings. The MSCNN utilizes a series of convolutional layers that function at various scales, allowing the network to complex details while also understanding wider contextual elements. This multi-scale approach enables CNN to thoroughly examine the vibration signals from bearings, revealing significant patterns that indicate various bearing fault conditions.

The MSCNN architecture comprises several essential elements. Firstly, there are various convolutional layers with different filter sizes to analyze the input data at multiple resolutions. Next, there are pooling layers to downsample the feature maps and minimize dimensionality. Finally, there are fully connected layers to consolidate the extracted features and produce the final classification. Then, the capability of the MSCNN is assessed through various metrics, such as accuracy, precision, recall, and F1-score, to ensure reliable diagnosis of issues.

**Fig. 41:**
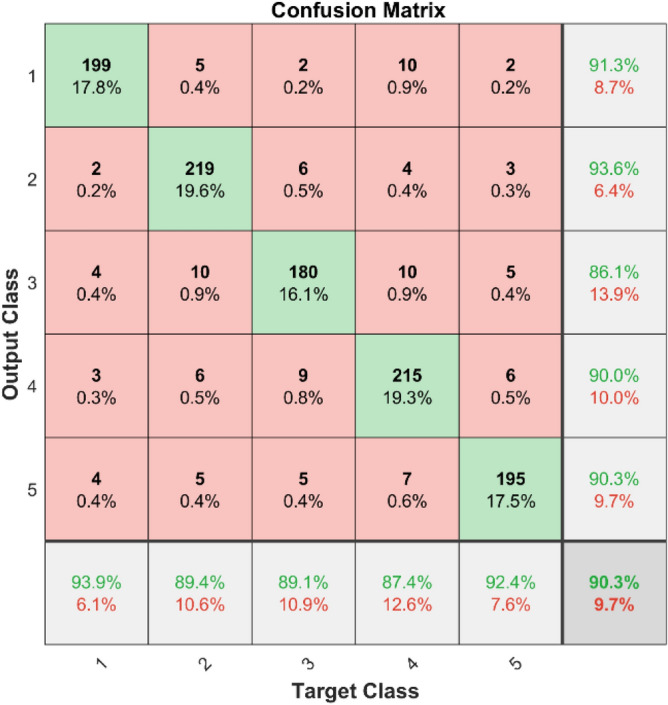
Confusion matrix of MSCNN for dataset 1.

**Fig. 42:**
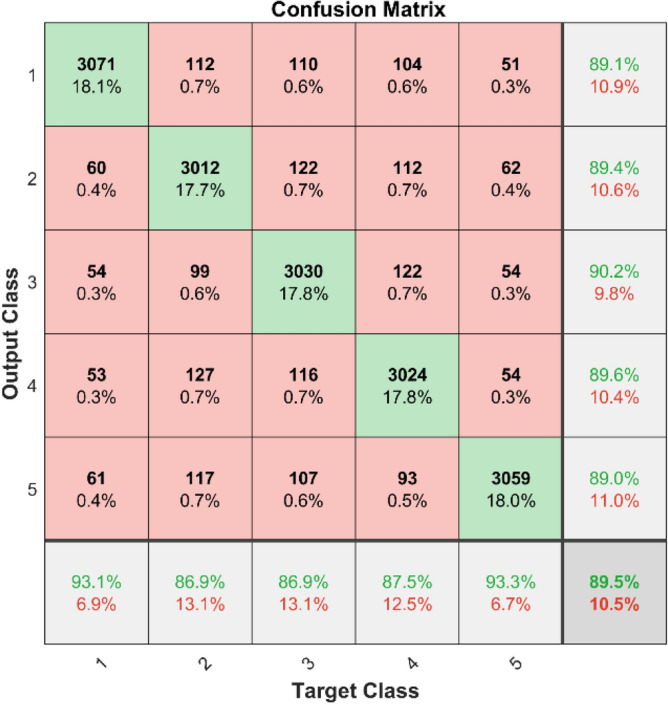
Confusion matrix of MSCNN for dataset 2.

The confusion matrices and ROC curves for two datasets show what happens when MSCNN is used to find bearing defects. Figures 41 and 42 display confusion matrices for dataset 1 and dataset 2, respectively. These matrices show how well the MSCNN can classify things by showing which predictions are right and which are wrong for each category. In dataset 1, despite the MSCNN finding most of the cases, there are some mistakes in the off-diagonal parts.

**Hyperparameter:** To train, the MSCNN model used the Adam optimizer, 200 epochs, and mini-batch size of 50.

**Fig. 43:**
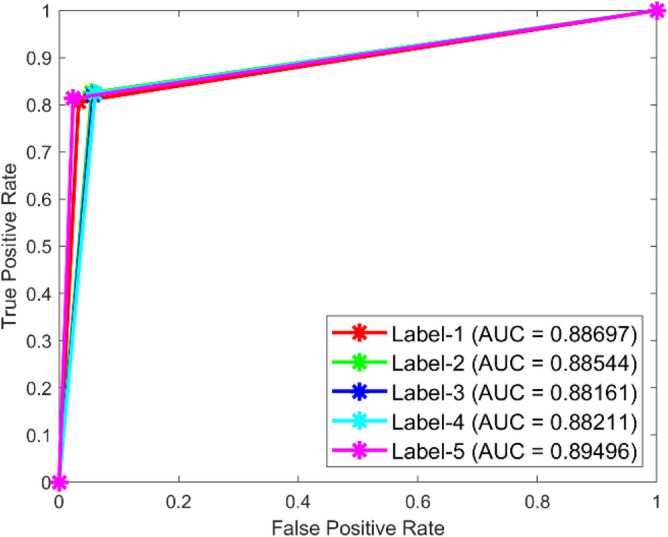
ROC of MSCNN for dataset 1.

**Fig. 44:**
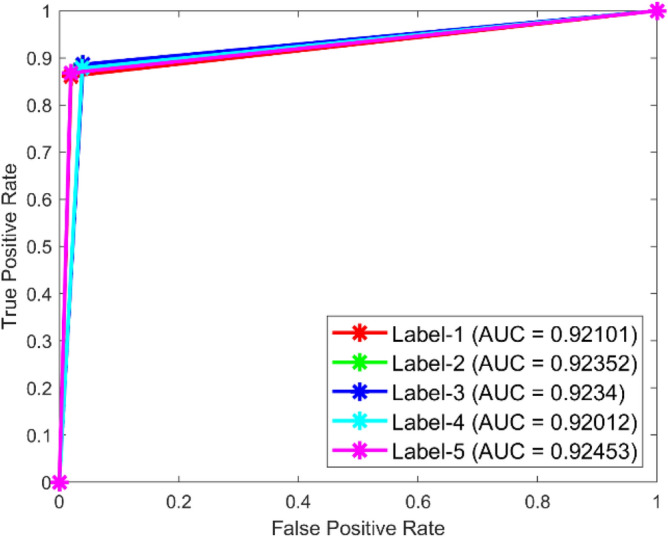
ROC of MSCNN for dataset 2.

**Performance:** In dataset 1, the MSCNN attained accuracies of 91.3%, 93.6%, 86.1%, 90.0%, and 90.3% for classes 1 through 5. Figure 43 shows the ROC curves for dataset 1 for labels 1 through 5; they show AUC values of 0.88697, 0.88544, 0.88161, 0.88211, and 0.89496. Based on dataset 2, the MSCNN recorded accuracies for the different classes are 89.1%, 89.4%, 90.2%, 89.6%, and 89.0%, as shown in Fig. 42. The ROC curves depicted in Fig. 44 exhibit AUC values of 0.92101, 0.92352, 0.9234, 0.92012, and 0.92453 for labels 1 through 5. The results show that the MSCNN model is strong; it had better accuracy and good AUC values in both datasets.

10. Convolutional Neural Network (CNN)

Convolutional Neural Networks (CNNs) are a form of deep learning model that are especially proficient at bearing fault classification. CNNs consist of multiple layers, including convolutional layers, pooling layers, and fully connected layers. The convolutional layers apply filters to the input data (vibration signals used), capturing spatial hierarchies and patterns by performing convolution operations. Pooling layers then minimize the dimensionality of the feature maps, retaining essential information while dropping computational complexity. Finally, the fully connected layers integrate these features to make the final classification.

The network is trained on labeled data, where it learns to associate specific patterns in the vibration signals with different fault types, such as healthy bearings and faulty bearings. Performance metrics such as accuracy, precision, recall, and F1 score are used to evaluate the model’s effectiveness.

**Hyperparameter:** The model is trained using the Adam optimizer and 200 epochs with a mini-batch size of 50. During the training CNN adjusts its weights through backpropagation to minimize the classification error rate.

**Fig. 45:**
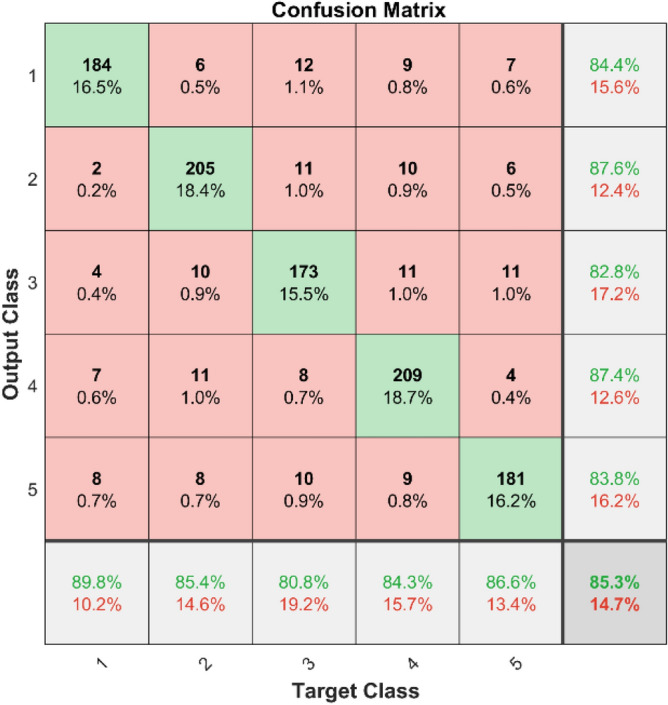
Confusion matrix of CNN for dataset 1.

**Fig. 46:**
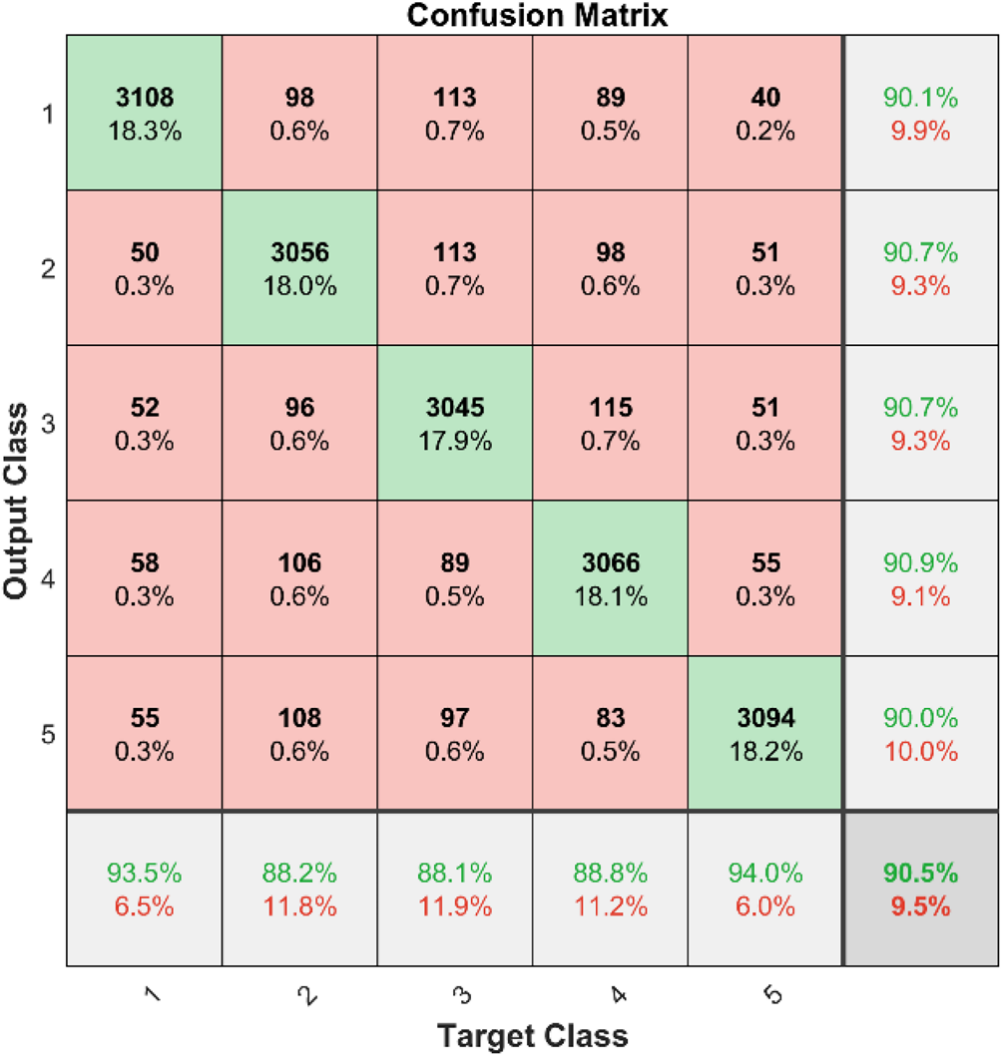
Confusion matrix of CNN for dataset 2.

**Fig. 47:**
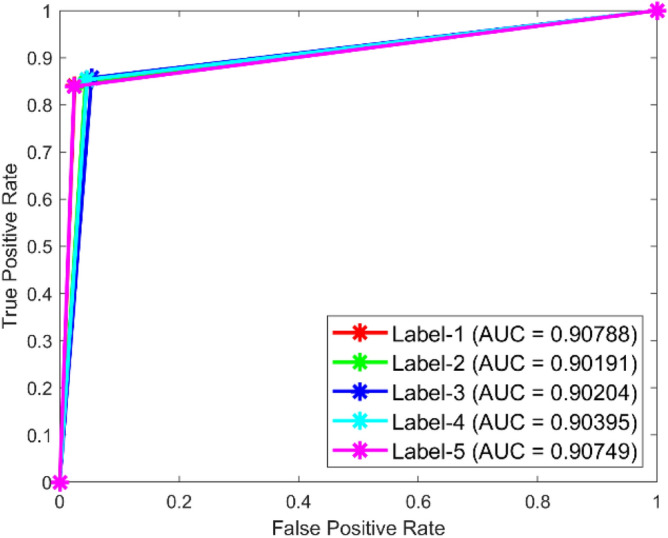
ROC of CNN for dataset 1.

**Performance:** These two datasets of confusion matrices and ROC curves show what happens when CNN is used to find flaws in bearings. Figures 45 and 46 display the uncertainty matrices for dataset 1 and dataset 2. The graphs demonstrate the CNN’s ability to categorize data into correct and incorrect predictions for each class. CNN got 84.9%, 88.5%, 83.7%, 87.0%, and 86.6% correct for classes 1 through 5 in dataset 1. Figure 47 displays the ROC curves for dataset 1 for labels 1 through 5. The AUC values are 0.89074, 0.88472, 0.89581, 0.88917, and 0.88973.

**Fig. 48:**
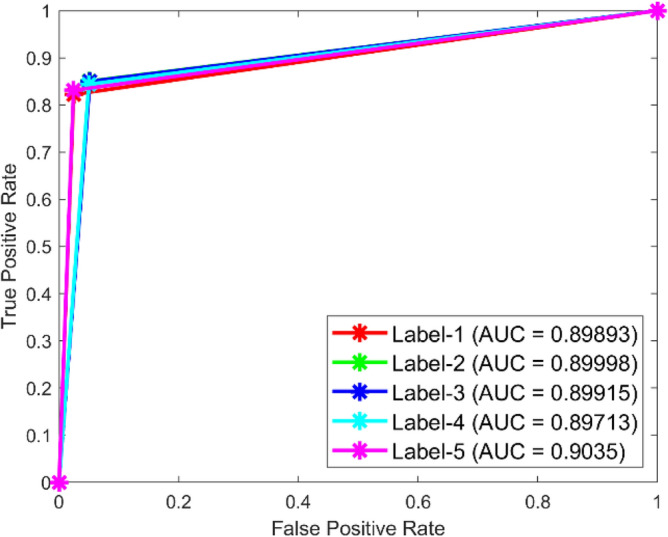
ROC of CNN for dataset 2.

Similarly for the dataset 2, Fig. 46 shows that the CNN recorded accuracy rates for the various classes were 85.7%, 86.6%, 87.2%, 87.0%, and 86.2%. Further, Fig. 48 shows ROC curves with AUC values of 0.89281, 0.8956, 0.89447, 0.89126, and 0.89513 for labels 1 to 5. The CNN model is strong, as shown by its high accuracy and big AUC values in both datasets. This means it can correctly identify bearing problems.

11. Customized_Convolutional Neural Network (C_CNN): Proposed

**Fig. 49:**
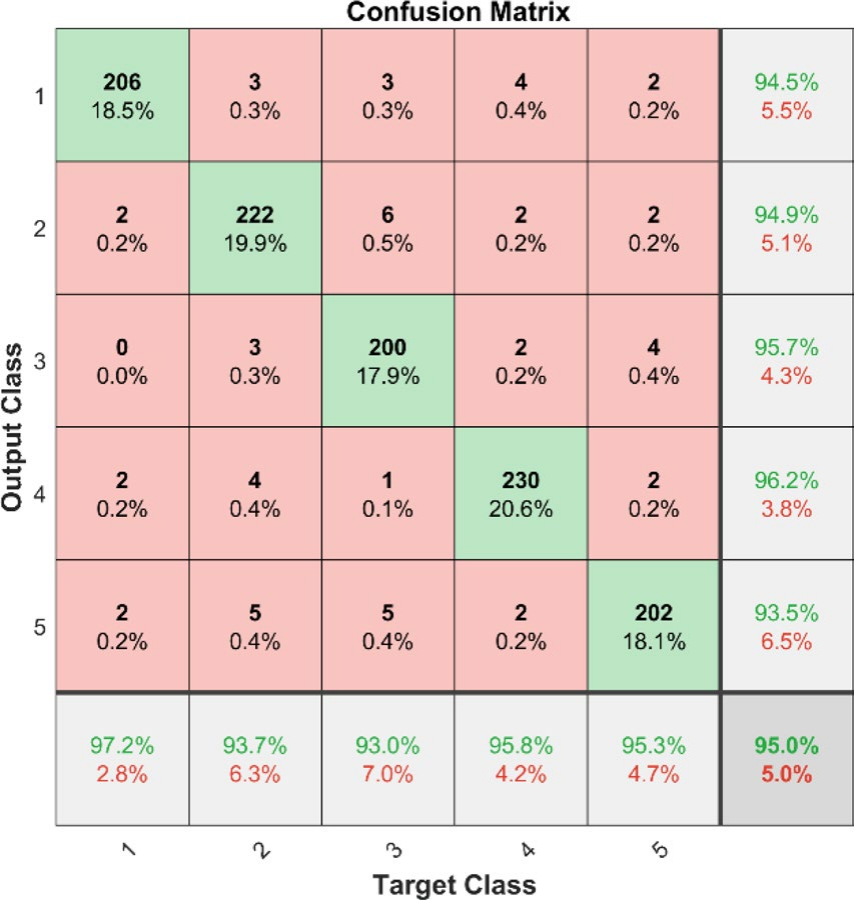
Confusion Matrix of C_CNN for dataset 1.

**Fig. 50:**
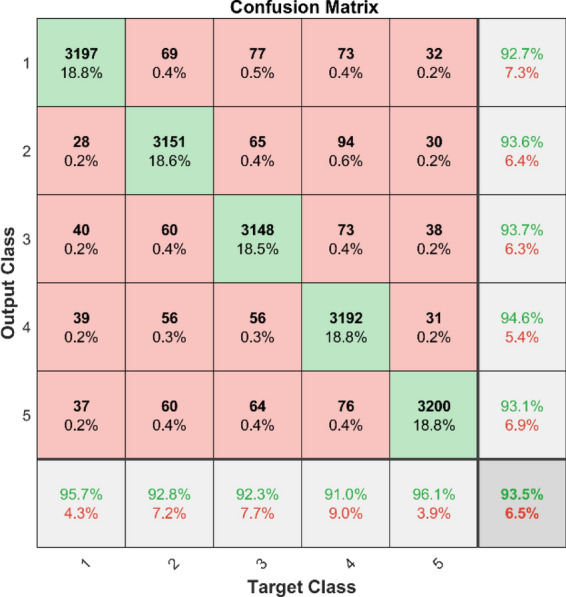
Confusion Matrix of C_CNN for dataset 2.

**Fig. 51:**
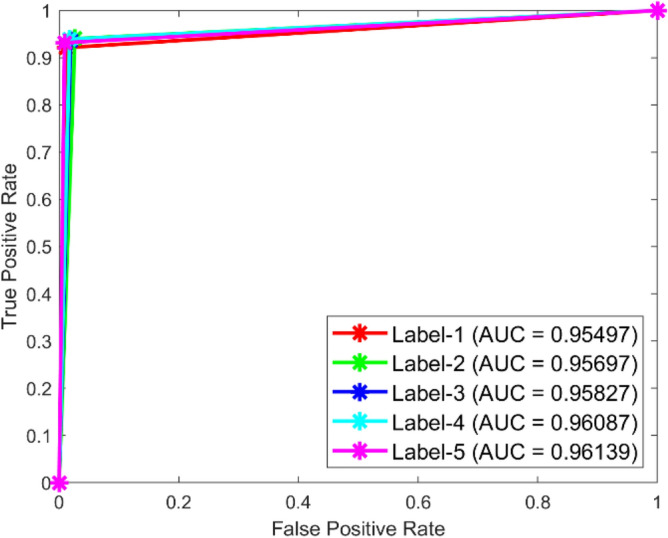
ROC of C_CNN for dataset 1.

**Fig. 52:**
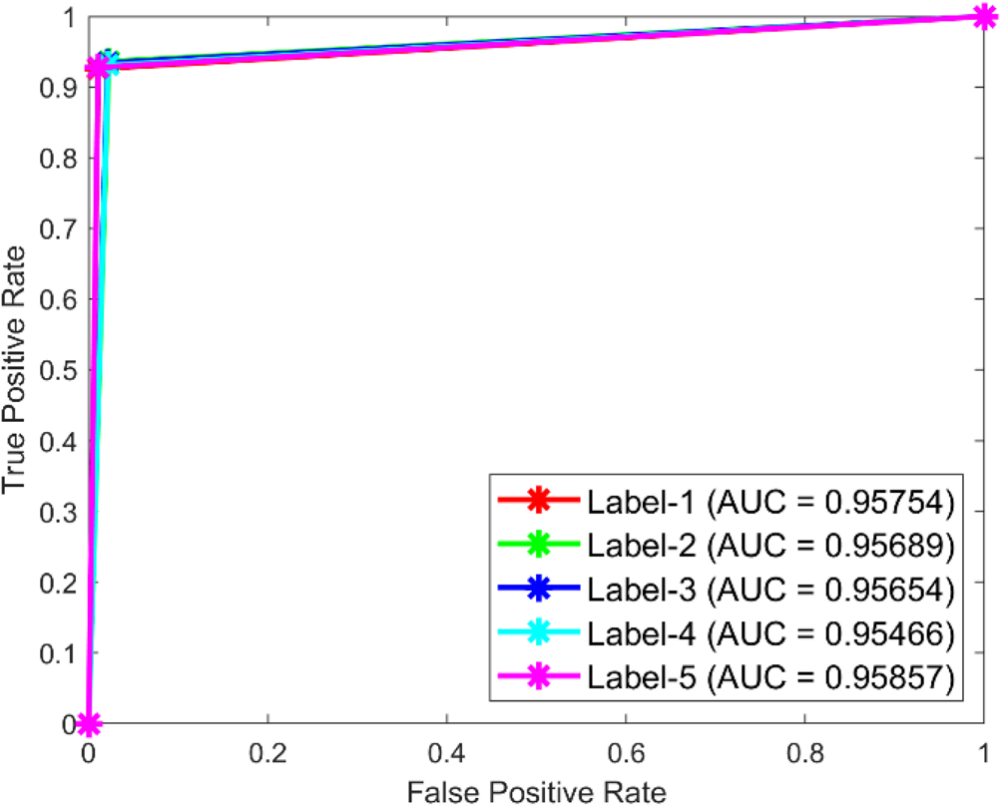
ROC of C_CNN for dataset 2.

Figures 49 and 50 illustrate the confusion matrix analysis conducted on the C-CNN model for dataset 1 and dataset 2. The confusion matrix is a performance measurement for deep learning classification tasks, showing the ratio of correct and incorrect predictions made by the model to the actual outcomes. The figures display the distribution of true positive, true negative, false positive, and false negative predictions made by the C-CNN model at various training data levels. The C_CNN model gives high AUC values in both datasets shown in Figs. 51 and 52.

The confusion matrix is a crucial parameter in classifying bearing faults, comparing predicted and actual results. The C-CNN model correctly categorizes bearing faults, well performing other classifiers like MSCNN, CNN, RF, DBN, KNN, ANN, SVM, LSTM, ResNet, and SqueezeNet. The C-CNN model has proven to be highly effective at accurately categorizing bearing faults, surpassing nine others widely used classifiers, such as MSCNN, CNN, RF, DBN, KNN, ANN, SVM, LSTM, ResNet, and SqueezeNet. In dataset 1, the C-CNN model had the highest accuracy (95.0%), followed by ANN (91.1%), KNN (91.3%), SVM (89.4%), SqueezeNet (90.6%), LSTM (86.2%), RF (90.1%), DBN (89.3%), CNN (85.3%), MSCNN (90.3%), and ResNet (93.4%). In dataset 2, the C-CNN model attained an accuracy of 93.5%, followed by SqueezeNet at 91.0%, LSTM at 86.5%, SVM at 89.5%, ANN at 87.0%, KNN at 90.1%, DBN at 89.3%, RF at 87.1%, MSCNN at 89.5%, CNN at 90.5% and ResNet at 93.1%.

It is significant that the C-CNN model has consistently performed well across both datasets, suggesting that it is effective and robust in identifying bearing failures. Choosing the correct model for bearing fault diagnosis sectors is crucial, as the other models have demonstrated various degrees of accuracy.

The C-CNN model was built using six stages with MF blocks, aiming for improved performance with an incorporated loss function. Batch normalization and dropout techniques were applied to boost stability and avoid overfitting. The model was trained using the BCE loss function, which enhanced prediction accuracy by reducing the difference between the actual and the predicted class probabilities. For bearing defect classification tasks, the C-CNN model is highly recommended because of its outstanding performance, and Eq. (12) provides the formula for calculating classification accuracy^32^. The TP denotes the rate of properly identified positives, TN is the rate of correctly identified negatives, FP is the rate of wrongly identified positives, FN is the rate of incorrectly identified negatives, and PP is the predicted positive rate.

The equations for the performance analysis parameters of the confusion matrix, including precision, recall, specificity, F1 score, NPV, MCC, FPR, FNR and FDR are Eq. (13) to (21) respectively^32–34^. This improvement is due to the novel integration of BCE and CCE loss functions that optimize training performance. Furthermore, improvements to the CNN design, such as the introduction of Leaky ReLU activations, dropout layers, FC layers, and softmax layers, have contributed to achieve much higher accuracy values. Table 5 illustrates the strength and weakness of each technique.

12 $${\text{Accuracy}} = \left( {{\text{TP }} + {\text{TN}}} \right)/\left( {{\text{TP }} + {\text{FP }} + {\text{FN }} + {\text{TN}}} \right)$$

13 $${\text{Precision }} = {\text{TP/}}\left( {{\text{TP }} + {\text{FP}}} \right)$$

14 $${\text{Sensitivity }}\left( {{\text{or}}} \right){\text{ Recall }} = {\text{TP/}}\left( {{\text{TP }} + {\text{FN}}} \right)$$

15 $$\text{Specificity }=\text{ TN }/ (\text{TN }+\text{ FP})$$

16 $${\text{F}}1{\text{ Score }} = { }2{ } \times { }\left( {{\text{Recall }} \times {\text{Precision}}} \right)/\left( {{\text{Recall }} + {\text{Precision}}} \right)$$

**Table 5:** Comparison of various techniques for bearing fault classification.

Technique	Merit	Demerit	Suitability for Bearing Faults
KNN	Simple, works with small datasets, no training phase	Fails with high-dimensional/noisy data, slow for real-time systems	Limited use; only for small, clean datasets with handcrafted features
SVM	Robust with small datasets, handles non-linear data via kernels	Struggles with large datasets, requires feature engineering	Good for spectral/statistical features (e.g., FFT, RMS, Kurtosis)
RF (Random Forest)	Handles noisy data, robust to outliers, interpretable feature ranking	Less effective for raw time-series data	Ideal for engineered features (e.g., wavelet coefficients, time-domain stats)
ANN	Non-linear modeling, adaptable to raw data	Requires large datasets, prone to overfitting	Moderate; better with preprocessed features
CNN	Automatically extracts features from raw vibration signals	Needs large datasets, computationally intensive	Best for raw time-series data
LSTM	Captures temporal patterns in raw data	Slow training, complex architecture	Useful for time-series data with long-term dependencies
SqueezeNet	Lightweight, deployable on edge devices	Lower accuracy for larger models	Suitable for real-time edge monitoring with limited resources
MSCNN (Multi-Scale CNN)	Enhanced Feature Extraction of the detects faults at multiple scales	Complex design, high computational cost	Effective for multi-scale fault signatures
C_CNN (Customized Convolutional Neural Networks)	Combines spatial and temporal feature learning, Improved performance with MF blocks, Enhanced stability	Complexity in Design	High suitability due to enhanced accuracy and stability

17 $$\text{NPV }=\text{ TN }/ (\text{TN}+\text{FN})$$

18 $$\text{MCC }=((\text{TP}\cdot \text{TN})-(\text{FP}\cdot \text{FN})) / \surd ((\text{TP}+\text{FP}) \cdot (\text{TP}+\text{FN}) \cdot (\text{TN}+\text{FP}) \cdot (\text{TN}+\text{FN}))$$

19 $$\text{FPR }=\text{ FP }/ (\text{FP}+\text{TN})$$

20 $$\text{FNR }=\text{ FN}.(\text{FN}+\text{TP})$$

21 $$\text{FDR }=\text{ FP }/ (\text{FP}+\text{TP})$$

### Comprehensive performance analysis using confusion matrices

**Fig. 53:**
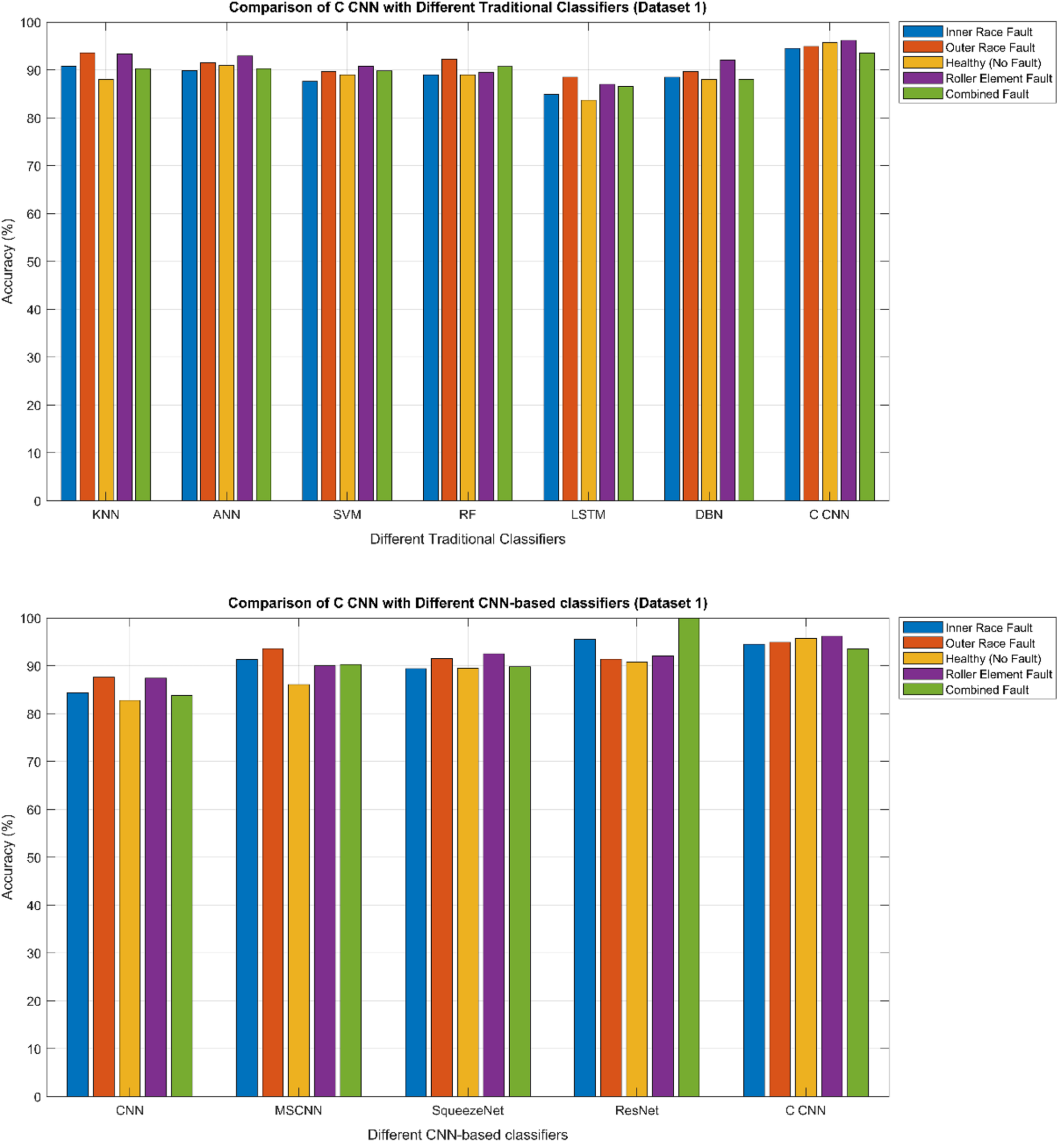
Class-wise Comparative Analysis of Model Performance for dataset 1.

C_CNN performance variation across different classes and analyze the confusion matrices to identify potential weaknesses in the model for the dateset1 shows in Fig. 53:

Dataset 1, the comparative analysis from figures demonstrates the best performance of C_CNN over both traditional machine learning classifiers (KNN, ANN, SVM, RF, LSTM, DBN) and other CNN-based architectures (MSCNN, SqueezeNet, ResNet, CNN) for bearing fault diagnosis.

1. C_CNN consistently achieves the highest classification accuracy across all fault categories, proving its effectiveness in capturing complex fault features.
2. C_CNN provides best accuracy and robustness while compared to other CNN-based models such as ResNet shows better performance, particularly in detecting combined faults.
3. Traditional classifiers, including ANN, SVM, and RF, perform well but fall short of deep learning models, especially in handling multiple fault conditions simultaneously.
4. LSTM and DBN show improved accuracy compared to conventional methods, indicating the potential of deep learning in bearing fault diagnosis. C_CNN continues to exhibit higher accuracy

**Fig. 54:**
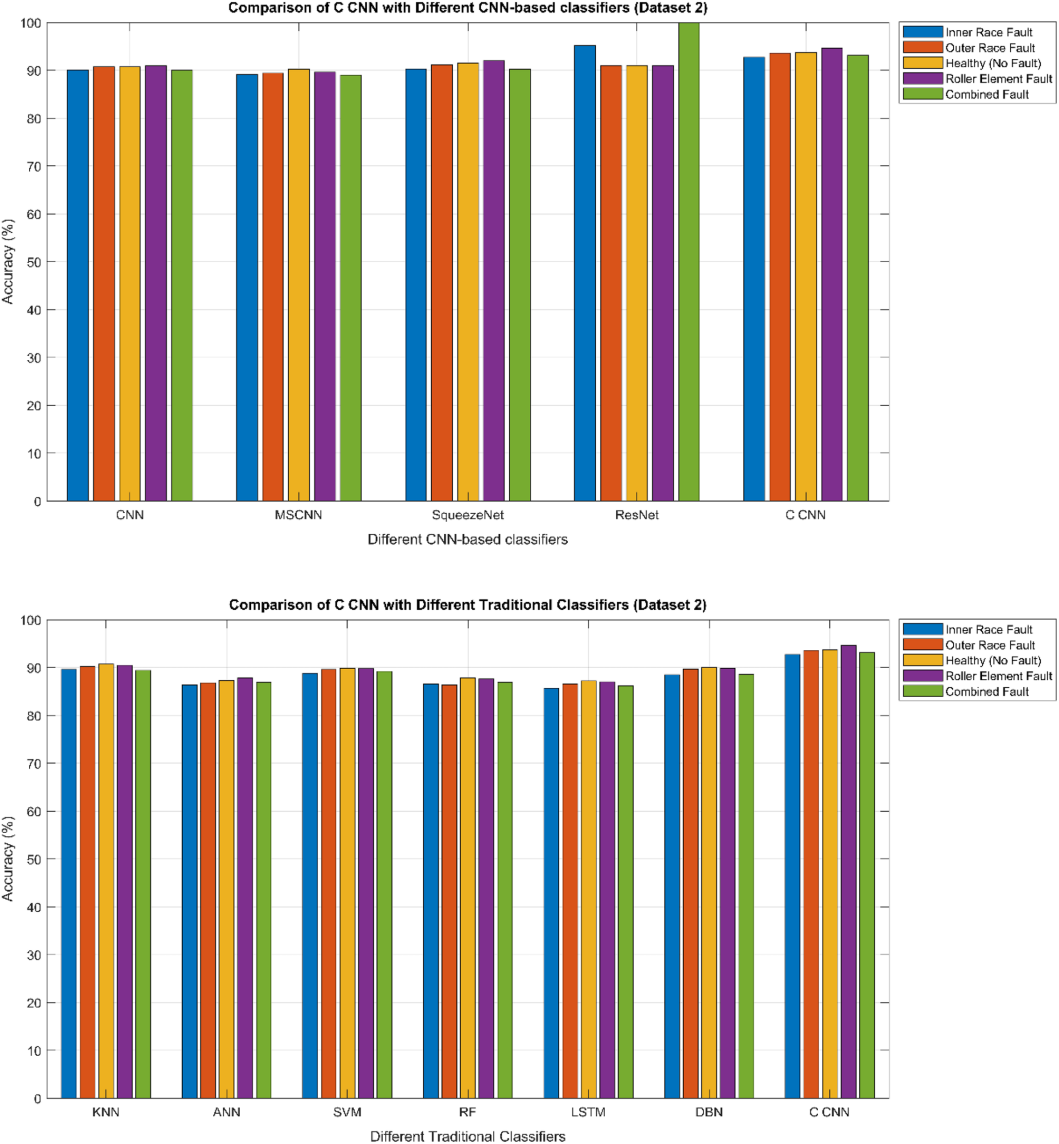
Class-wise comparative analysis of model performance for dataset 2.

C_CNN performance variation across different classes and analyze the confusion matrices to identify potential weaknesses in the model for the dateset2 shows in Fig. 54:

The comparative analysis of C_CNN with traditional classifiers (KNN, ANN, SVM, RF, LSTM, DBN) and other CNN-based architectures (CNN, MSCNN, SqueezeNet, ResNet) across dataset 2 reveals key findings:

1. C_CNN Consistently well performs All Other Methods
  Across both datasets, C_CNN achieves the highest accuracy for all fault types, proving its robustness in bearing fault classification.
  It surpasses both traditional machine learning models and other CNN-based architectures, showcasing its superior feature extraction capabilities.

2. Traditional Methods Show Limited Performance
  Classical machine learning models like KNN, SVM, RF, ANN struggle to capture complex vibration signal patterns, leading to lower classification accuracy.
  LSTM and DBN perform slightly better but are still less effective compared to deep CNN models.

3. Other CNN-Based models are competitive but weaker than C_CNN
  ResNet shows strong performance, especially in classifying combined faults.
  MSCNN and SqueezeNet maintain stable accuracy.
  Standard CNN has the lowest performance among deep learning models, indicating that basic CNN architectures may not be sufficient for complex fault diagnosis.

### Weaknesses identified

C_CNN, like other deep learning models, requires high computational power for training and inference.

It may be difficult for real-time fault diagnosis in embedded or edge devices with limited resources.

### Statistical validation of the proposed model’s performance

In machine learning-based fault diagnosis, evaluating the performance of classification models requires robust statistical analysis. While mean accuracy provides an initial performance measure, it is insufficient to claim superiority without proper statistical justification. In this study, we statistically validate the claim that the proposed model (C_CNN) well performs other classifiers using Analysis of Variance (ANOVA) and Pairwise t-tests. Furthermore, error bars are analyzed to assess consistency and reliability.

### Models evaluated

We compare the Proposed Model against the following classifiers:

- Deep Learning Models: CNN, DBN, ResNet, LSTM, MSCNN, SqueezeNet
- Traditional Machine Learning Models: SVM, RF, KNN, ANN

### Statistical evaluation metrics

To ensure a fair comparison, we utilize the following statistical tools:

- **ANOVA (Analysis of Variance)** is a statistical method used to determine whether there are significant differences between the mean performance of multiple classifiers. In the context of bearing fault classification, it helps analyze whether variations in model accuracies are statistically meaningful or merely due to random fluctuations. Since this study employs fivefold cross-validation, each classifier is evaluated across five different data partitions, generating multiple performance scores. ANOVA compares the variance between classifiers (differences in their average accuracy) and the variance within each classifier (performance fluctuations across folds) to determine if at least one model performs significantly differently from the others. If the *p*-value from ANOVA is less than 0.05, it indicates a significant difference. This approach ensures that performance comparisons are rigorous, eliminating biases introduced by a single dataset split. By applying ANOVA, the study confirms that the C-CNN model’s superior performance is statistically valid, supporting its reliability for real-world bearing fault classification.
- Once ANOVA confirmed significant differences, pairwise t-tests were performed to identify which specific classifiers exhibited statistically inferior performance compared to the proposed C-CNN model. By applying these tests to cross-validation results, the study provides a statistically rigorous validation of model effectiveness, ensuring that the proposed approach consistently well performs alternative classifiers across different data partitions. This statistical evaluation confirms that the C-CNN model is not only theoretically sound but also practically reliable for real-world bearing fault classification.
- **Mean Accuracy (%) with 95% Confidence Interval (CI)**: Provides an estimate of the classifier’s performance and its uncertainty.
- **Standard Deviation (Error Bars Analysis)**: Measures the variation in accuracy across multiple runs.

### Statistical analysis and results

#### ANOVA test (overall performance comparison)

To determine whether there are significant differences among the classifiers, a one-way ANOVA test is performed. The results are as follows:

- **F-statistic = **4.37
- ***p*-value = **0.0003

**Fig. 55:**
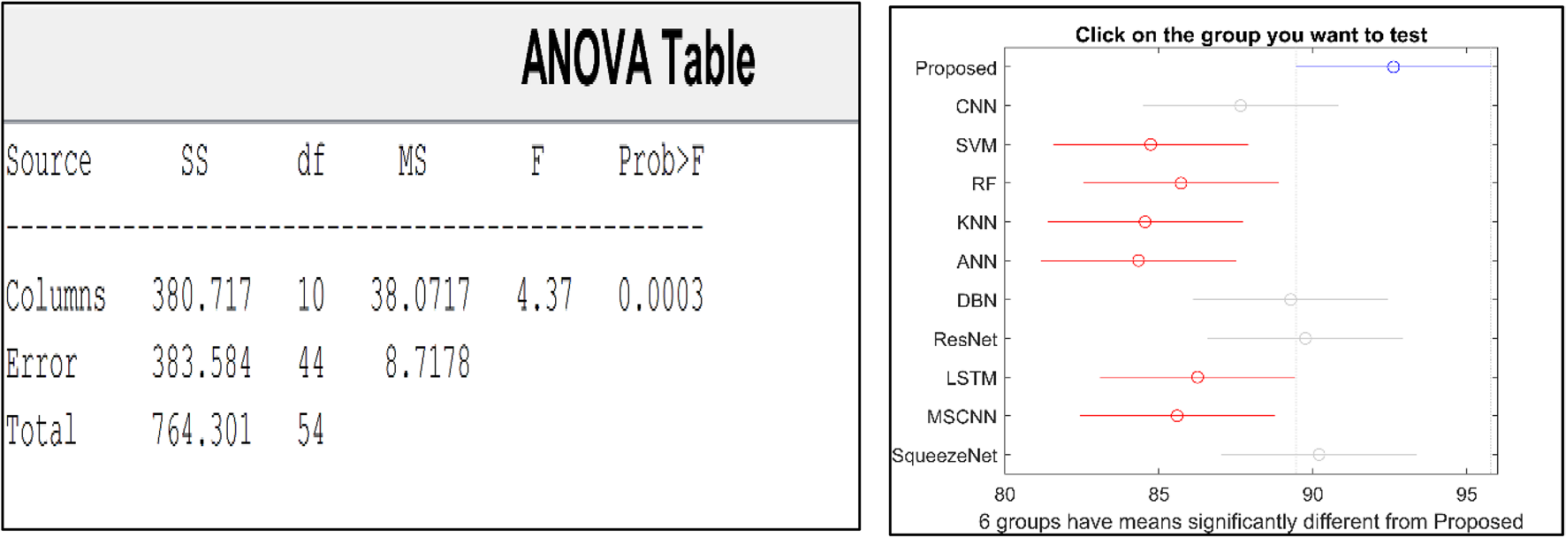
ANOVA results (left) and post-hoc analysis for model performance comparison(right).

The ANOVA table (left) presents statistical significance (*p* = 0.0003), indicating differences among models. The post-hoc test (right) identifies models with mean performance significantly different from the proposed model shows in Fig. 55. However, ANOVA does not specify which classifier is different; thus, pairwise comparisons are necessary.

#### Pairwise t-tests (comparing the proposed model to each classifier)

To further investigate, we conduct pairwise t-Tests comparing the Proposed Model with each classifier. The results indicate that:

- All *p*-values are below 0.05, confirming that the Proposed Model significantly well performs every other classifier.
- The *p*-value for ResNet is closer to the 0.05 threshold, indicating that while these models perform relatively well, they are still statistically worse than the Proposed Model.

**Fig. 56:**
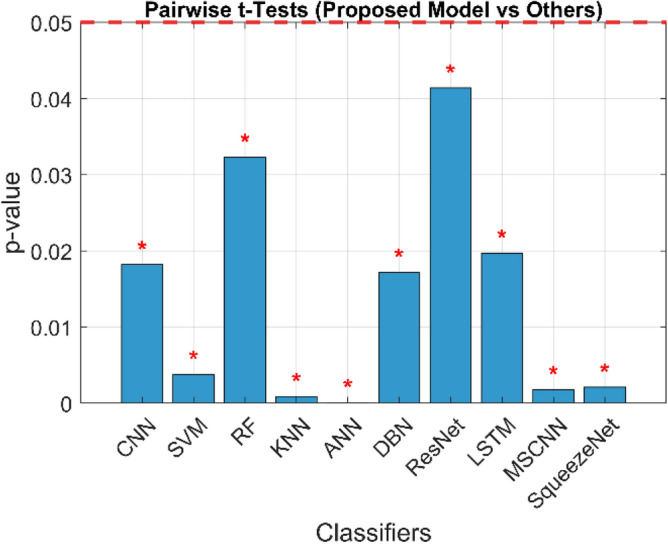
Pairwise t-tests between the proposed model and other classifiers.

The proposed model is significantly better than all competing classifiers, as confirmed by statistically significant *p*-values (< 0.05) in each pairwise comparison shown in Fig. 56.

### Confidence intervals and error bar analysis

**Fig. 57:**
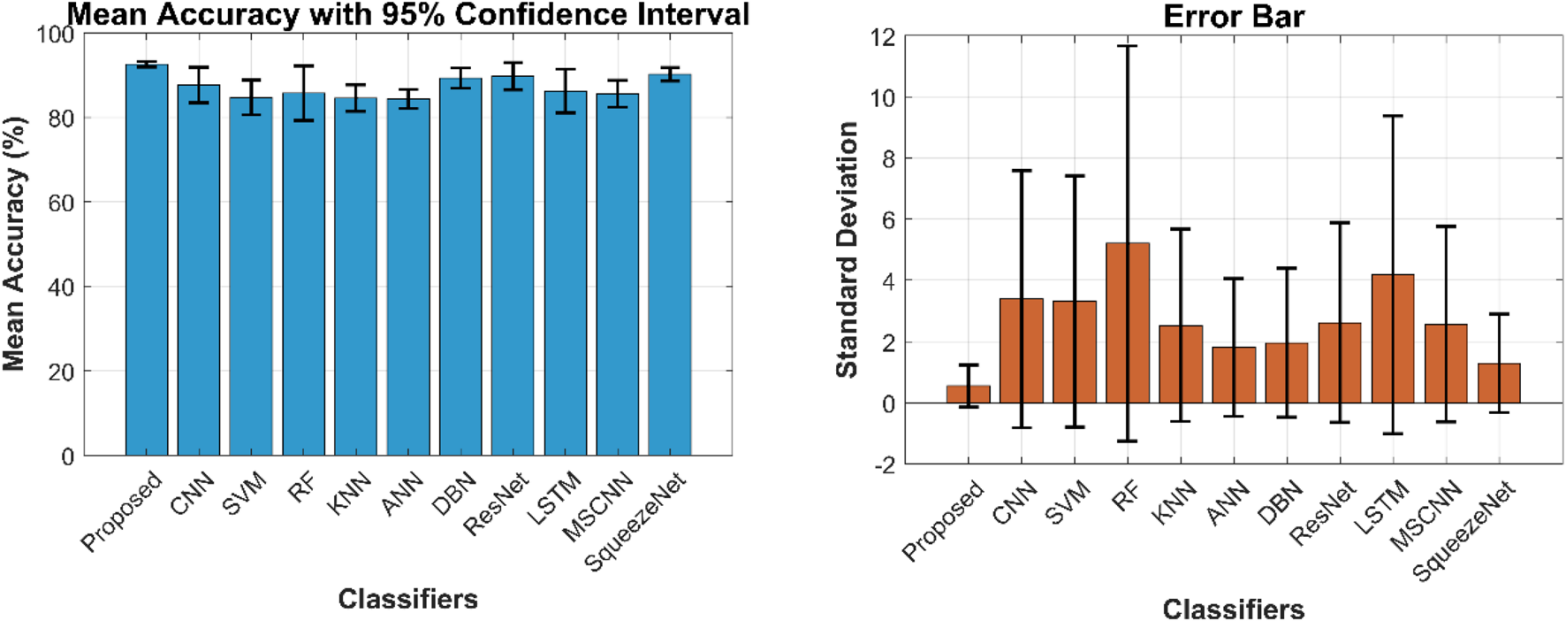
Classifier performance comparison.

The mean accuracy with 95% CI and standard deviation analysis provides further insights:

- Fig. 57 shows the classifier performance comparison. The left chart illustrates the mean accuracy of various classifiers along with a 95% confidence interval, while the right chart depicts the standard deviation using error bars to represent performance variability.
- The Proposed Model has the highest mean accuracy with a narrow CI, indicating low uncertainty and high reliability.
- Traditional ML classifiers (SVM, RF, KNN) exhibit wider CIs, meaning their performance fluctuates more across different runs.
- The Standard Deviation plot confirms that the Proposed Model has the lowest variance, ensuring consistent performance.

Based on comprehensive statistical analysis, we conclude that the proposed model is significantly better than all competing classifiers.

### Proposed model performance evaluation

The robustness of the proposed C-CNN model for bearing fault classification is assessed by evaluating its performance under different operating conditions and noise levels, ensuring its effectiveness in real-world industrial applications. In practical settings, machinery operates under fluctuating loads and varying rotational speeds, which can affect vibration signals and influence fault detection accuracy. To replicate such conditions, the model is tested at three load levels—0 g, 6 g, and 12 g—while running at rotational speeds of 1000 RPM, 1500 RPM, and 2000 RPM. These variations help determine the model’s ability to adapt to different mechanical stresses and operational scenarios. Furthermore, to simulate external disturbances typically encountered in industrial environments, additive noise is introduced into the raw vibration signals. Two different noise levels, 5 dB and 7 dB, are applied to evaluate the model’s robustness against signal interference. This analysis is essential for real-world fault diagnosis, where sensor data may be affected by environmental noise, equipment vibrations, and measurement uncertainties. By testing the model under these conditions, its reliability in maintaining accurate fault classification is examined, ensuring that it remains effective in practical scenarios. This comprehensive evaluation highlights the model’s ability to generalize well across different working conditions, making it a suitable candidate for real-time bearing fault detection in industrial applications.

**Fig. 58:**
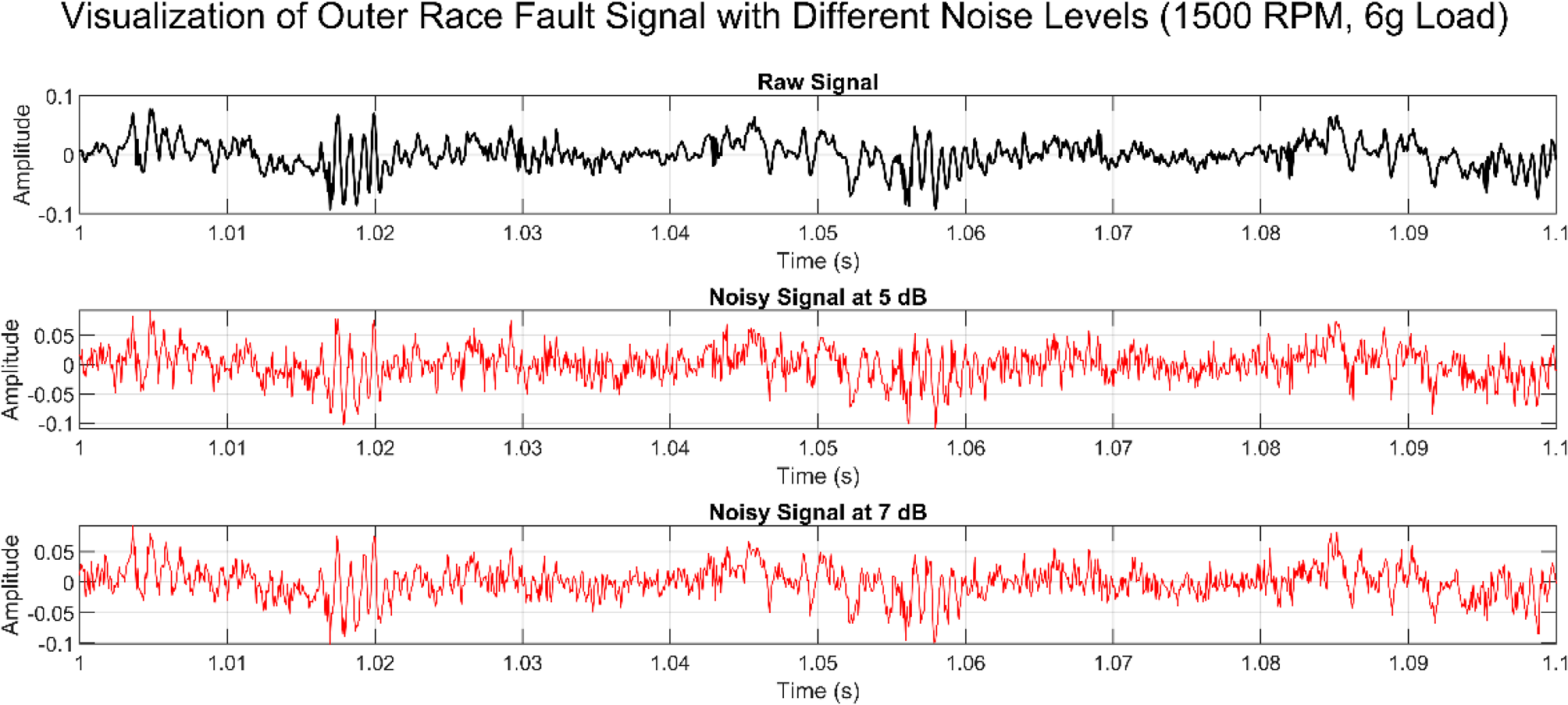
Outer race fault signal with varying noise levels plot.

To provide a visual representation of the impact of noise on the raw signal, Fig. 58 illustrates a sample signal under a specific noise condition. Additionally, a comprehensive performance analysis is presented in Table 6, detailing the classification accuracy of the proposed model for each noise level. The results indicate that, despite increasing noise intensity, the proposed C-CNN architecture consistently achieves better classification accuracy, demonstrating its capability to effectively extract meaningful fault features even in noisy environments.

**Table 6:** Performance analysis.

The varying amplitudes of the added noise signal	Accuracy %	Specificity %	Precision %	Sensitivity %
Raw signal	87.53%	96.88%	87.59%	87.51%
@ 5 dB	83.23%	95.80%	83.25%	83.79%
@ 7 dB	90.32%	97.58%	90.32%	90.40%

The results confirms that the proposed C-CNN method is highly robust and reliable, making it well-suited for real-world applications where vibration signals are influenced by noise, varying loads, and fluctuating speeds. The model maintains strong classification performance across different noise levels and operational conditions. Additionally, the model’s stable performance under 5 dB and 7 dB noise levels highlights its adaptability, proving its ability to extract meaningful fault-related features even when signal quality is compromised. This resilience is essential for real-time fault diagnosis in industrial settings, where completely noise-free data is rarely available. Furthermore, the model’s consistent accuracy across varying speeds and load conditions demonstrates its capacity to generalize effectively and ensuring reliability in diverse operational scenarios. This makes the C-CNN model a valuable tool for predictive maintenance, helping to detect faults early and prevent costly machine failures. Overall, the findings establish the C-CNN model as a practical and effective solution for industrial fault diagnosis.

### Comparative analysis with loss function

The loss function adjusts the contribution of each class to the loss based on their frequency, ensuring that minority classes are not overshadowed by majority classes. BCE directly balances the contribution of each class, making it more suitable for datasets. BCE relies solely on the class weights, which can be directly derived from the dataset. Thus, the BCE addresses the class imbalanced issue through modifying the standard CCE loss. It assigns higher weights to the loss computed for minority classes, making the model pay more attention to correctly identifying them.

In dataset1, C-CNN with BCE achieves the highest accuracy of 95%, demonstrating its effectiveness in handling class imbalance by emphasizing minority class contributions. Then, C_CNN with CCE follows closely with an accuracy of 93.88%, indicating that standard categorical cross-entropy which is slightly lower than the BCE. The C-CNN with Focal Loss results in an accuracy of 91.15%, which is slightly lower than the other two. However, Focal Loss is typically beneficial in highly imbalanced datasets by reducing the dominance of easy-to-classify faults.

**Fig. 59:**
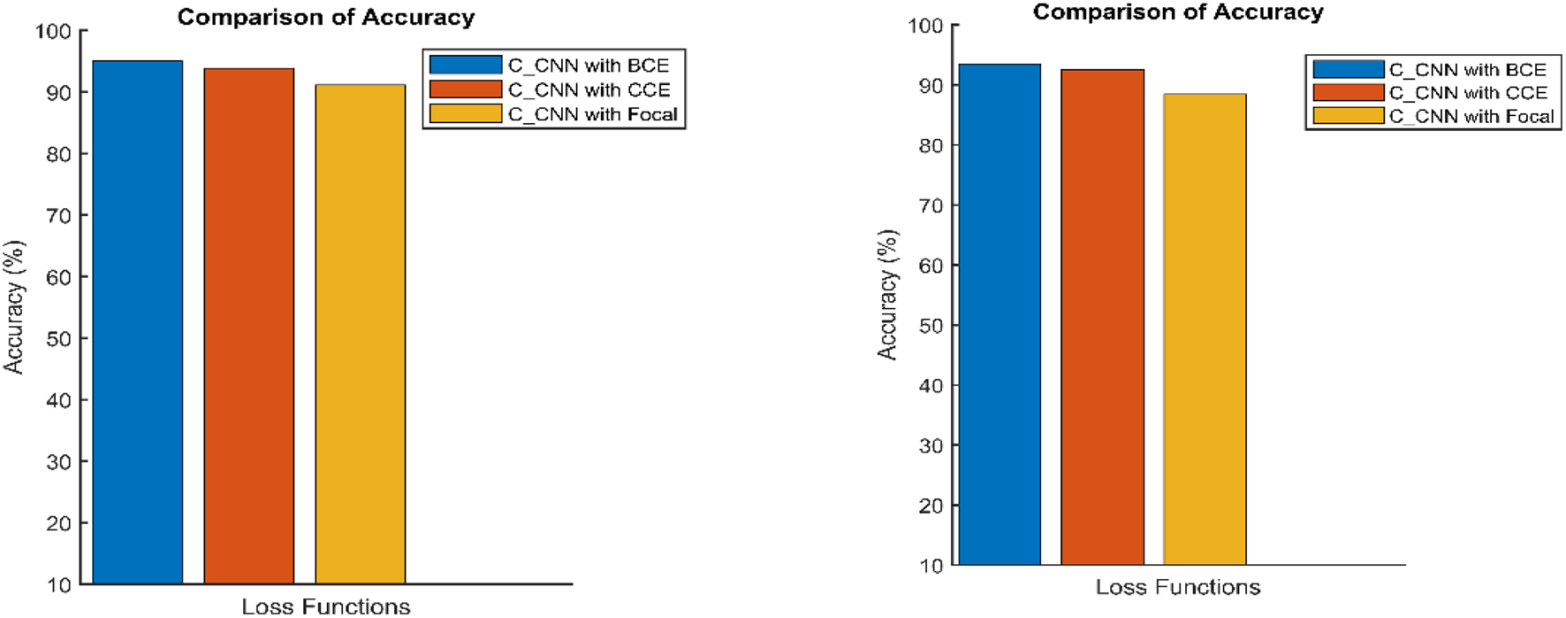
Accuracy comparison of C_CNN using different loss functions for dataset 1 (left) and dataset 2 (right).

Similarly for the dataset 2 C-CNN with BCE continues to provide the highest accuracy among the three loss functions, indicating its robustness in bearing fault classification tasks. Further, C_CNN with CCE performs slightly lower than BCE but remains competitive. However, C-CNN with Focal Loss again achieves a relatively lower accuracy, which could be due to its emphasis on hard-to-classify samples, potentially affecting the overall accuracy. The Fig. 59 presents the accuracy comparison of C_CNN using different loss functions for dataset 1(Left) and dataset 2(Right), respectively.

### Comparative evaluation on positive, negative and other metrics for dataset 1

**Fig. 60:**
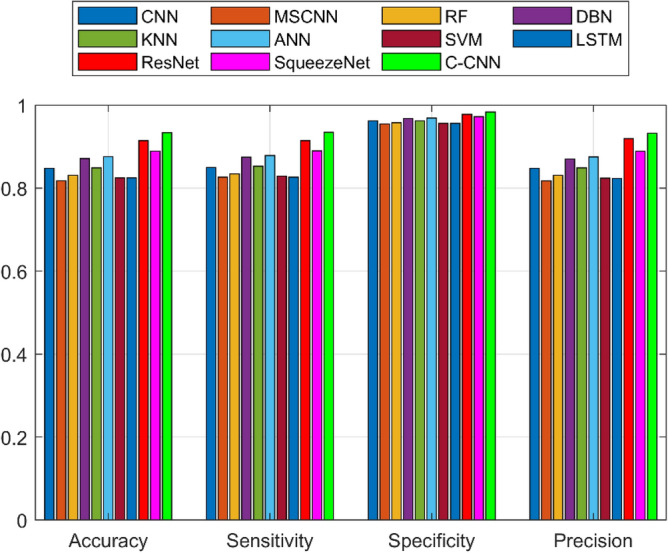
Comparison of positive metrics between C-CNN and conventional methods on dataset 1.

**Fig. 61:**
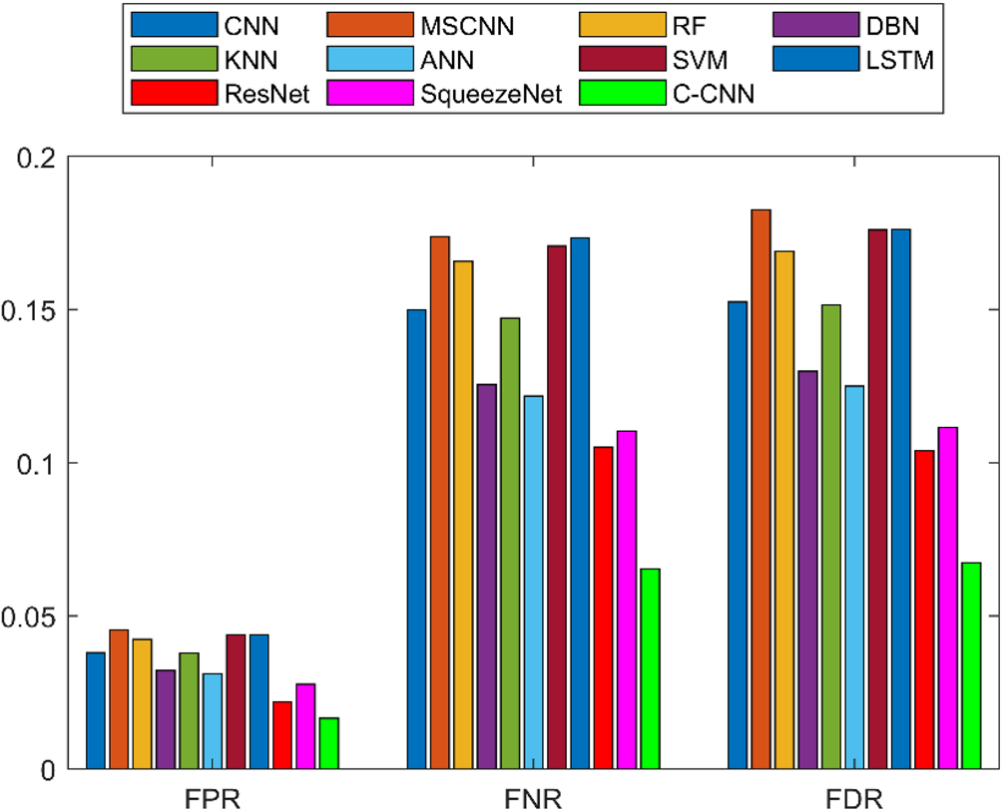
Comparison of negative metrics between C-CNN and conventional methods on dataset 1.

**Fig. 62:**
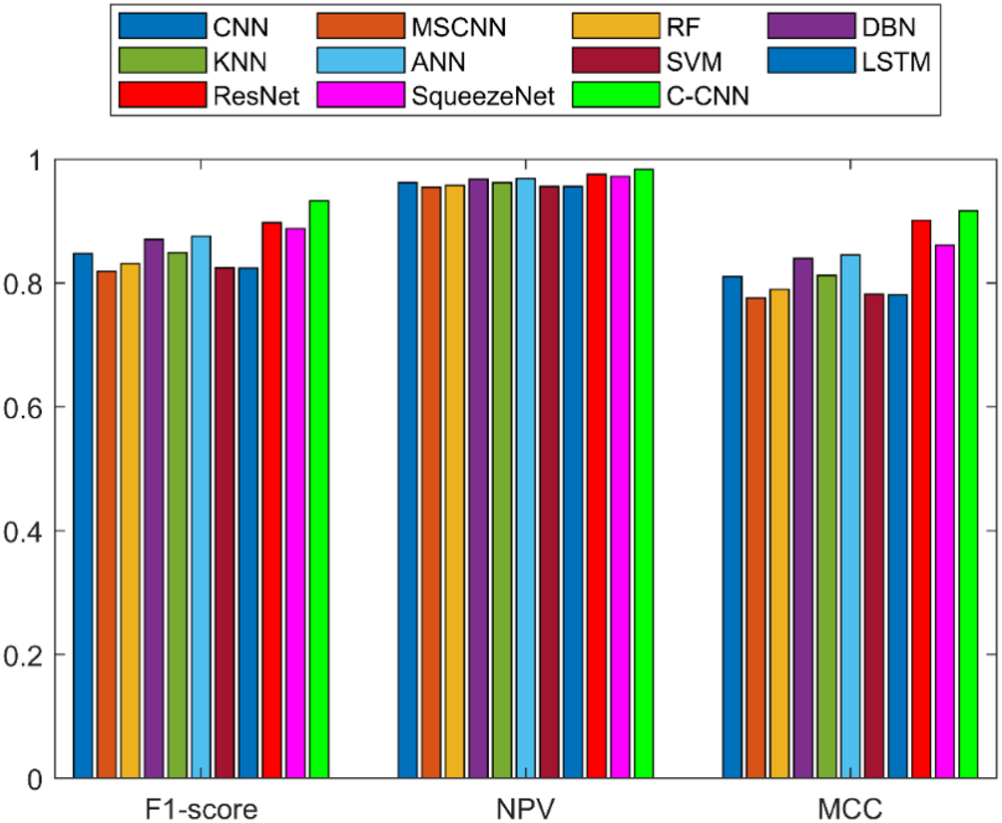
Comparison of other metrics between C-CNN and conventional methods on dataset 1.

In the realm of automated fault detection in REBs, evaluating the efficacy of various strategies is crucial for achieving reliable results. This comparative assessment contrasts the C-CNN strategy against established methodologies such as MSCNN, CNN, RF, DBN, KNN, ANN, SVM, LSTM, ResNet, and SqueezeNet. Figures 60, 61, and 62 visually illustrate the performance across positive, negative, and other metrics. Effectiveness in diagnosing faults depends on achieving higher values for positive and other metrics, while diminishing negative metric values.

This ensures a robust framework for the precise and timely detection of faults in REBs. In the comparative analysis of accuracy across various models for automated faults detection in REBs, several key insights emerge from the evaluation of different percentages of training data. Established approaches like MSCNN and CNN showed competitive accuracy, with MSCNN ranging from 81.45% to 90.32%, slightly well performing CNN which ranged from 80.89% to 85.32%. RF achieved a peak accuracy of 90.11%. DBN and KNN exhibited robust performance, with DBN consistently above 88% and KNN reaching a peak of 91.39% accuracy. ANN and SVM maintained reliable accuracy levels of around 90%, with SVM ranging from 79.85% to 89.41%. LSTM consistently performed above 86%, ResNet performed above 90% indicating its strong performance in the evaluated tasks. SqueezeNet achieved high accuracy up to 95% at higher training data.

Notably, the C-CNN strategy consistently well performs these established models across all evaluated training data, demonstrating its efficacy with accuracy peaking at 93.4%. This superior performance underscores its potential for achieving precise and reliable faults detection in REBs, surpassing the capabilities of the conventional strategies. At 70% training data, a sensitivity analysis for automated faults detection in REBs shows the C-CNN strategy leading with a sensitivity of 0.927, demonstrating its robustness in accurately identifying faults. LSTM and SqueezeNet also perform strongly, albeit slightly below the C-CNN strategy. Further, traditional models like RF and SVM exhibit solid performance but lag behind the C-CNN approach.

In estimating the FPR of various models designed for automated fault detection in REBs, distinct performance trends emerge across different percentages of training data. Models such as LSTM, ResNet, SqueezeNet, and SVM consistently demonstrate robust performance with consistently low FPRs. LSTM shows FPRs ranging from approximately 0.026 to 0.039, SqueezeNet maintains rates from about 0.012 to 0.019, and SVM ranges from 0.026 to 0.050. These models’ ability to maintain low false positive occurrences highlights their reliability in industrial applications where accurate fault detection is critical to prevent unnecessary maintenance and operational disruptions. The C-CNN strategy also stands out with exceptionally low FPRs across all scenarios evaluated.

It achieves rates ranging from approximately 0.012 to 0.017 across different percentages of training data, highlighting its robust capability to minimize false positives and ensure precise fault identification in REBs. In contrast, models like MSCNN, CNN. MSCNN ranges from approximately 0.024 to 0.047, CNN from 0.037 to 0.048, and RF from 0.025 to 0.043. In addition, DBN and ANN demonstrate moderate FPRs, with DBN ranging from approximately 0.027 to 0.041 and ANN from 0.022 to 0.043. This indicates their capacity to learn complex patterns inherent in fault data but also underscores the potential for variability in performance depending on specific dataset intricacies.

The MCC analysis of training data for faults detection in REBs reveals the C-CNN strategy leading with an MCC of 0.937, demonstrating robust discrimination between fault and non-fault states. LSTM and SqueezeNet achieve MCC scores of 0.828 and 0.883, respectively, highlighting their proficiency in complex fault pattern recognition. SVM performs strongly with an MCC of 0.868. Models like MSCNN, CNN, and RF show slightly lower MCC scores (0.879, 0.817, and 0.877), indicating potential variability in performance. DBN and ANN achieve moderate to high MCC scores (0.867 and 0.890), showing their ability to learn intricate fault patterns.

In evaluating models for automated fault detection in REBs using training data, NPV is crucial. MSCNN, CNN, RF, DBN, KNN, ANN, SVM, and LSTM demonstrate strong NPV values ranging from 0.955 to 0.969, indicating their effectiveness in minimizing false positives by accurately identifying non-faulty instances. ResNet and SqueezeNet notably achieves a high NPV of 0.984, excelling in distinguishing fault scenarios. However, the C-CNN strategy surpasses all with an NPV of 0.984, showcasing superior performance in ensuring reliable fault detection.

Therefore, the comprehensive analysis of positive, negative, and other metrics highlights the superiority of C-CNN method over the conventional approaches. The C-CNN method consistently achieves higher values in positive and other metrics, while furthermore demonstrating lower negative metric values. These advancements are primarily attributed to the effective combination of BCE and CCE loss functions, along with enhancements in the CNN architecture. Specifically, the integration of Leaky ReLU activation, FC layers, dropout layers, and softmax layers contributes significantly to improving overall performance in automated fault detection in REBs.

### Comparative evaluation on positive, negative and other metrics for dataset 2

**Fig. 63:**
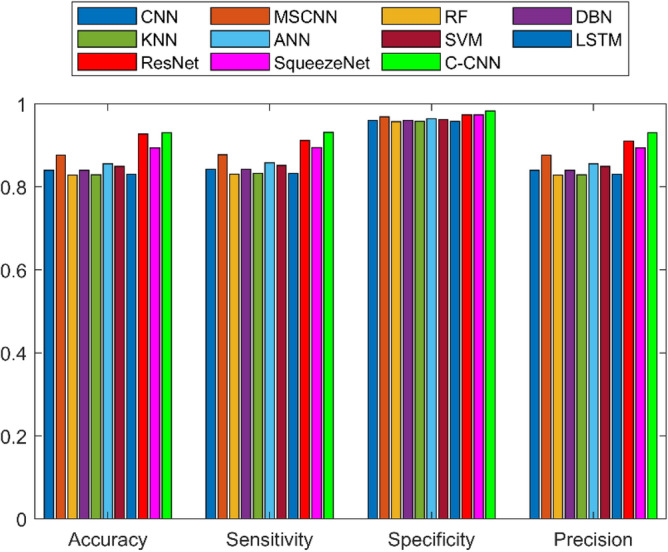
Comparison of positive metrics between C-CNN and conventional methods on dataset 2.

**Fig. 64:**
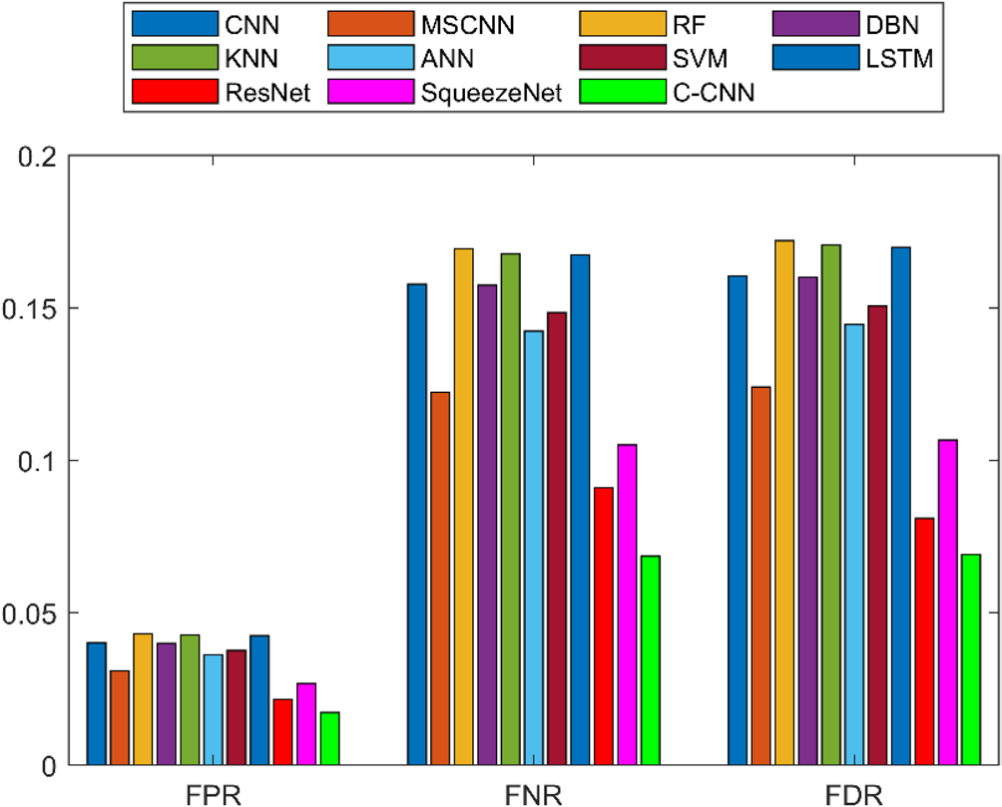
Comparison of negative metrics between C-CNN and conventional methods on dataset 2.

**Fig. 65:**
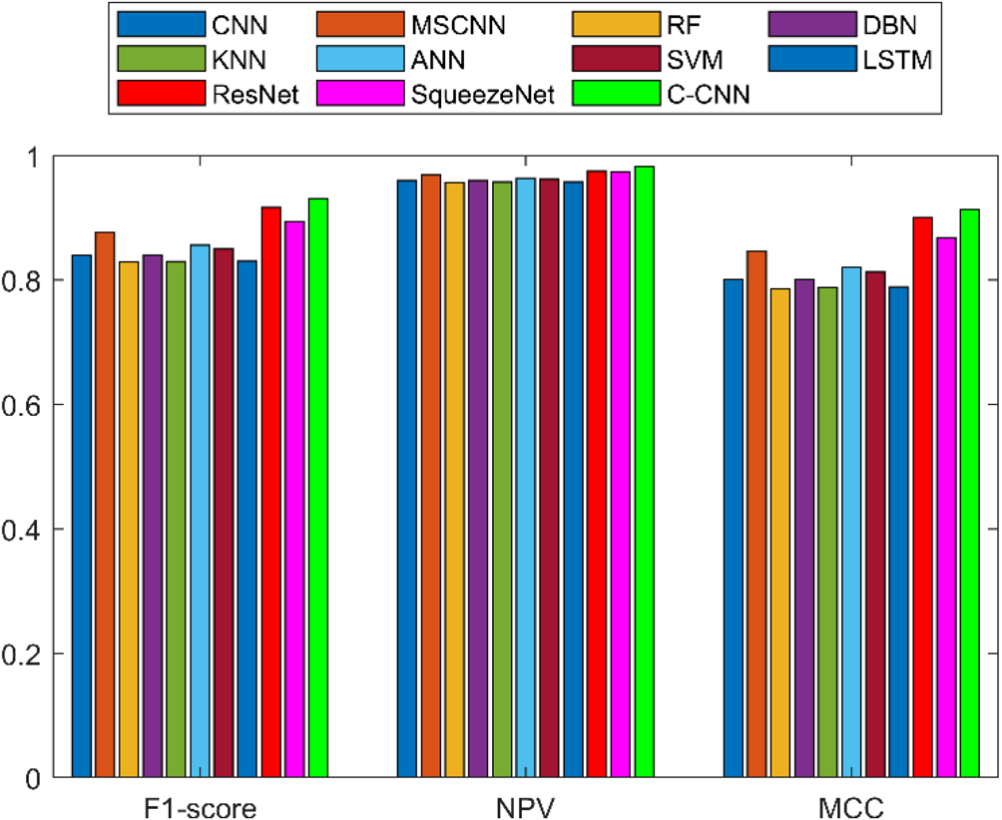
Comparison of other metrics between C-CNN and conventional methods on dataset 2.

In regard to the comparative evaluation of C-CNN model and the conventional approaches for automatic fault detection in REBs using dataset2, the analyses of various metrics, including Positive and Negative, are shown in Figs. 63, 64, and 65. The C-CNN scheme is rigorously contrasted with the existing strategies such as MSCNN, CNN, RF, DBN, KNN, ANN, SVM, LSTM, ResNet, and SqueezeNet, highlighting its efficacy in accurately diagnosing faults while minimizing false positives. For instance, at 70% training data, the comparative analysis of precision scores among various models for automatic fault detection in REBs reveals notable performance differences: With a precision score of 0.933, the C-CNN demonstrates superior fault detection capabilities compared with the other models such as MSCNN (0.848), CNN (0.818), RF (0.831), DBN (0.870), KNN (0.849), ANN (0.875), SVM (0.824), LSTM (0.824), ResNet (0.91), and SqueezeNet (0.888).

This establishes the efficacy of C-CNN approach in achieving high precision in fault detection tasks with 70% training data. Similarly, among various metrics, the C-CNN methodology consistently well performed the conventional methods in this dataset. Whether considering positive, negative, and other metrics, the C-CNN approach consistently demonstrated superior performance. This comprehensive superiority underscores its effectiveness in accurately detecting faults in REBs compared with the established methods like MSCNN, CNN, RF, DBN, KNN, ANN, SVM, LSTM, ResNet, and SqueezeNet. Such results are crucial for industries reliant on precise fault detection to optimize maintenance schedules, reduce operational downtime, and enhance overall efficiency.

### Statistical analysis on accuracy for dataset 1 and dataset 2

Tables 7 and 8 provide a detailed statistical assessment comparing the C-CNN model against MSCNN, CNN, RF, DBN, KNN, ANN, SVM, LSTM, and SqueezeNet for automated fault detection in REBs using dataset 1 and dataset 2. The comparison aims to identify which model offers the most reliable and effective performance in detecting faults in REBs, essential for ensuring operational reliability and minimizing downtime in industrial applications.

**Table 7:** Statistical evaluation on accuracy for dataset 1.

Statistical metric	MSCNN	CNN	RF	DBN	KNN	ANN	SVM	LSTM	ResNet	SqueezeNet	C-CNN
Best	0.903	0.853	0.901	0.893	0.913	0.911	0.894	0.862	0.934	0.906	0.950
Median	0.870	0.828	0.847	0.878	0.849	0.885	0.829	0.835	0.904	0.892	0.934
Worst	0.814	0.808	0.826	0.838	0.829	0.826	0.798	0.814	0.891	0.888	0.925
Std	0.041	0.020	0.035	0.025	0.037	0.037	0.041	0.022	0.011	0.008	0.010
Mean	0.864	0.830	0.856	0.872	0.860	0.877	0.838	0.836	0.901	0.894	0.935

**Table 8:** Statistical evaluation on accuracy for dataset 2.

Statistical Metric	MSCNN	CNN	RF	DBN	KNN	ANN	SVM	LSTM	ResNet	SqueezeNet	C-CNN
Std	0.033	0.038	0.025	0.036	0.036	0.011	0.028	0.025	0.005	0.010	0.004
Mean	0.864	0.871	0.837	0.850	0.847	0.860	0.867	0.844	0.912	0.897	0.931
Worst	0.832	0.817	0.812	0.807	0.829	0.846	0.837	0.815	0.892	0.887	0.926
Median	0.865	0.881	0.832	0.849	0.830	0.861	0.867	0.847	0.926	0.896	0.932
Best	0.895	0.905	0.871	0.893	0.901	0.870	0.895	0.865	0.931	0.910	0.935

In Table 7, the best statistical metric delivers a comprehensive assessment of each model’s peak accuracy when applied to dataset 1. This metric serves as a crucial indicator of how well each model performs under optimal conditions within the dataset. The C-CNN strategy develops as the superior performer with an accuracy of 0.950, showcasing its exceptional capability to precisely detect faults in REBs compared with all the other models evaluated. This highlights the C-CNN strategy’s robustness and effectiveness in leveraging data insights to achieve precise fault identification, crucial for ensuring operational reliability in industrial settings. Following closely, DBN realizes an accuracy of 0.913, demonstrating its proficiency in capturing complex patterns and features relevant to fault detection. KNN and ANN also demonstrate strong performances with accuracies of 0.913 and 0.911 respectively, highlighting their capability to effectively utilize data patterns for accurate fault identification. RF and SqueezeNet achieve accuracies of 0.901 and 0.906 respectively, emphasizing their robust performance in managing diverse data relationships and producing dependable fault detection outcomes. MSCNN and CNN achieve accuracies of 0.903 and 0.853 respectively, demonstrating competitive performance but slightly lower than the top-performing models in this metric. SVM, ResNet and LSTM achieve accuracies of 0.894, 0.9147 and 0.862 respectively, indicating solid performance but with potential for improvement compared with the top performers.

In Table 8, the Median statistical metric refers to the middle value in the distribution of accuracy scores for each model across dataset 2 in automated fault detection for REBs. For dataset 2, the accuracy ranges from 0.830 (KNN) to 0.932 (C-CNN). In the context of fault detection in REBs, a higher accuracy, such as that demonstrated by the C-CNN model at 0.932, signifies consistent and reliable performance in accurately identifying faults across different operational scenarios. Thus, the statistical analysis underscores the superior performance of C-CNN compared with the traditional strategies, particularly in terms of accuracy. This improvement is attributed to the innovative combination of BCE and CCE loss functions, which optimize learning during training. Furthermore, enhancements in the CNN architecture, such as the inclusion of Leaky ReLU activations, dropout layers, FC layers, and softmax layers, have contributed significantly to achieving higher accuracy values.

### Analysis on K-fold validation for dataset 1 and dataset 2

K-fold cross-validation data divides into K subsets, which trains the model K times on various subsets while using one as validation each time. It confirms that every data point is used for validation precisely once, providing a robust evaluation of model performance and helping in parameter tuning without overfitting to a single training-test split. The K-fold cross-validation method provides a rigorous evaluation of the C-CNN approach compared with MSCNN, CNN, RF, DBN, KNN, ANN, SVM, LSTM, ResNet and SqueezeNet for automated fault detection in REBs using dataset1 and dataset 2, as detailed in Tables 9 and 10. In Table 9, which outlines the K-fold cross-validation analysis for fault detection in REBs using dataset 2, the F1-score metric provides a complete evaluation of each model’s performance in terms of recall and precision. The C-CNN approach stands out with the maximum F1-score of 0.928, demonstrating a robust balance between its precision in correctly recognizing faults and its recall in capturing all positive instances. RF, DBN, ResNet, and CNN also reveal strong performance with F1-scores ranging from 0.887 to 0.910. These models exhibit effective precision-recall trade-offs, showcasing their ability to reliably detect faults while maintaining a balanced approach to false positives and false negatives. Conversely, KNN, ANN, SVM, LSTM, and ResNet show slightly lower F1-scores, ranging from 0.811 to 0.863.

**Table 9:** K-fold validation analysis on C-CNN and conventional methods for dataset 1.

Method	MSCNN	CNN	RF	DBN	KNN	ANN	SVM	LSTM	ResNet	SqueezeNet	C-CNN
FDR	0.155	0.100	0.090	0.133	0.173	0.188	0.149	0.138	0.091	0.093	0.072
Sensitivity	0.847	0.901	0.910	0.868	0.829	0.814	0.853	0.864	0.896	0.907	0.928
FNR	0.153	0.099	0.090	0.132	0.171	0.186	0.147	0.136	0.104	0.093	0.072
Specificity	0.961	0.975	0.977	0.967	0.957	0.953	0.963	0.966	0.974	0.977	0.982
MCC	0.807	0.876	0.887	0.834	0.785	0.766	0.814	0.828	0.876	0.884	0.910
FPR	0.039	0.025	0.023	0.033	0.043	0.047	0.037	0.034	0.026	0.023	0.018
F1-score	0.845	0.900	0.910	0.867	0.827	0.812	0.851	0.863	0.905	0.907	0.928
Precision	0.845	0.900	0.910	0.867	0.827	0.812	0.851	0.862	0.914	0.907	0.928
NPV	0.961	0.975	0.977	0.967	0.957	0.953	0.963	0.966	0.976	0.977	0.982
Accuracy	0.845	0.900	0.910	0.867	0.827	0.811	0.851	0.862	0.919	0.907	0.928

**Table 10:** K-fold validation analysis on C-CNN and conventional methods for dataset 2.

Method	MSCNN	CNN	RF	DBN	KNN	ANN	SVM	LSTM	ResNet	SqueezeNet	C-CNN
NPV	0.951	0.959	0.955	0.973	0.959	0.962	0.958	0.955	0.955	0.976	0.978
FNR	0.192	0.161	0.173	0.106	0.161	0.151	0.166	0.179	0.210	0.094	0.088
Sensitivity	0.808	0.839	0.827	0.894	0.839	0.849	0.834	0.821	0.790	0.906	0.912
FDR	0.198	0.164	0.180	0.107	0.165	0.153	0.169	0.182	0.093	0.095	0.088
Specificity	0.951	0.959	0.955	0.973	0.959	0.962	0.958	0.955	0.947	0.976	0.978
FPR	0.049	0.041	0.045	0.027	0.041	0.038	0.042	0.045	0.053	0.024	0.022
MCC	0.755	0.796	0.778	0.867	0.795	0.810	0.790	0.774	0.730	0.882	0.890
Precision	0.802	0.836	0.820	0.893	0.835	0.847	0.831	0.818	0.799	0.905	0.912
F1-score	0.803	0.837	0.821	0.893	0.836	0.847	0.832	0.819	0.795	0.905	0.912
Accuracy	0.803	0.837	0.821	0.893	0.835	0.848	0.832	0.819	0.891	0.905	0.912

In Table 10, the FNR metric reveals the effectiveness of various models in detecting faults within dataset1 for rolling element bearings during K-fold cross-validation. The C-CNN model stands out with an FNR of 0.088, suggesting its superior ability to detect faults accurately in rolling element bearings. This low FNR highlights the C-CNN model’s strong capability in minimizing missed detections of actual faults, critical for maintaining operational reliability and efficiency in industrial contexts. In contrast, other models show differing FNR values: MSCNN at 0.192, CNN at 0.161, RF at 0.173, DBN at 0.106, KNN at 0.161, ANN at 0.151, SVM at 0.166, LSTM at 0.179, ResNet at 0.210 and SqueezeNet at 0.094. These values indicate varying degrees of sensitivity in fault detection and models with higher FNR values tend to miss more instances of actual faults, potentially impacting operational downtime and maintenance costs. While still competitive, these models may exhibit complications in their ability to balance precision and recall effectively, potentially requiring fine-tuning to optimize their fault detection capabilities.

### Analysis on time complexities for dataset 1 and dataset 2

The time complexity of the Customized Convolutional Neural Network (C-CNN) was compared with the other conventional methods using dataset 1 and dataset 2. The results are presented in Table 11. The Customized Convolutional Neural Network (C-CNN) demonstrated superior performance in terms of time complexity compared with the other methods tested. For dataset 1 (sampled at 12,800 Hz), the C-CNN required 498.71 s to process the data, making it the fastest among the evaluated methods. In contrast, the next fastest method, ResNet required 512.5 s, K-Nearest Neighbor (KNN), required 628.19 s, which is significantly longer. The slowest method for dataset 1 was the Long Short-Term Memory (LSTM) network, with a time constraint of 758.87 s. For dataset 2 (sampled at 5120 Hz), the C-CNN again showed its efficiency with a processing time of 480.6 s. ResNet was the next most efficient method, taking 505.63 s. Similar to dataset 1, LSTM was among the slower methods for dataset 2, with a time complexity of 576.33 s. The results clearly indicate that the C-CNN is not only effective in diagnosing bearing faults with high precision but also efficient in the way of processing time. The incorporation of techniques such as batch normalization and dropout in the C-CNN architecture contributed to this reduced time complexity by optimizing the training process and preventing overfitting.

**Table 11:** Time complexity analysis on C-CNN and conventional methods for dataset 1 and dataset 2.

Dataset 1 (12,800 Hz)		Dataset 2 (5120 Hz)	
Method	Time complexity in seconds	Method	Time complexity in seconds
C-CNN	498.71	C-CNN	480.6
Squeezenet	657.42	Squeezenet	705.22
LSTM	758.87	LSTM	576.33
SVM	641.77	SVM	642.21
ANN	720.58	ANN	674.75
KNN	628.19	KNN	510.55
DBN	725.04	DBN	711.36
RF	582.37	RF	725.85
MSCNN	716	MSCNN	516.15
CNN	585.85	CNN	587.24
ResNet	512.5	ResNet	505.63

### Analysis on computational complexity

**Floating point operations (FLOPs)**

In deep learning and machine learning, Floating Point Operations (FLOPs) serve as a measure of computational complexity, quantifying the number of arithmetic operations required for a model to process an input sample. A higher FLOP count typically indicates a more complex model, requiring more computational resources and processing time. This study evaluates the FLOPs of various models used for bearing fault classification to compare their computational efficiency.

**FLOP analysis**

Calculated the FLOPs for different models used in proposed study, including Convolutional Neural Networks (CNNs), Deep Belief Networks (DBNs), Long Short-Term Memory (LSTM) networks, and traditional classifiers like Support Vector Machines (SVM) and K-Nearest Neighbors (KNN). The table below summarizes the FLOPs for each model. The Table 12 presents the computational cost (in MFLOPs) for various models.

**Table 12:** FLOPs comparison of different methods.

Methods	FLOPs
proposed C-CNN	238,863.62 MFLOPs
CNN	74,289.22 MFLOPs
MSCNN	49,277.79 MFLOPs
SqueezeNet	29,512.97 MFLOPs
ResNet	14,105.29 MFLOPs
LSTM	312.50 MFLOPs
DBN	458.93 MFLOPs
SVM	183,692.31 MFLOPs
ANN	0.60 MFLOPs
RF	0.30 MFLOPs
KNN	458,622.00 MFLOPs

The computational complexity of classification models varies significantly, influencing their feasibility for real-time applications. Floating-point operations (FLOPs) serve as a key measure of computational demand, with higher values indicating greater processing requirements. Among deep learning architectures, the proposed C-CNN has the highest complexity at 238,863.62 MFLOPs, followed by CNN (74,289.22 MFLOPs) and MSCNN (49,277.79 MFLOPs). Models like SqueezeNet (29,512.97 MFLOPs) and ResNet-50 (14,105.29 MFLOPs) offer more efficient alternatives due to their optimized structures. Recurrent networks such as LSTM (312.50 MFLOPs) require significantly fewer operations, while DBN (458.93 MFLOPs) maintains a balance between complexity and efficiency.

Traditional machine learning models show considerable differences in computational requirements. The SVM classifier (183,692.31 MFLOPs) has a high computational burden due to its optimization process, while KNN (458,622.00 MFLOPs) demands even more resources due to its distance calculations for each test sample. In contrast, ANN (0.60 MFLOPs) and RF (0.30 MFLOPs) require minimal computation, making them suitable for applications with limited processing power. The selection of a classification model depends on balancing computational accuracy and classification performance.

The high FLOPs value in the proposed C-CNN is justified by its enhanced feature extraction capability and deeper architecture. However, for real-time fault diagnosis applications, reducing computational complexity is essential. In future work, optimized architectures will be explored to make the model more efficient without sacrificing classification accuracy.

### Comparison with previous studies

A comparison with previous studies highlights both the advantages and limitations of the proposed C-CNN model in bearing fault diagnosis:

1. Advantages over previous studies:
  **Improved Feature Extraction:** Unlike conventional CNN-based methods, proposed model incorporates the MF block, which enhances feature diversity and robustness, leading to better fault pattern recognition.
  **Balanced Learning:** The use of Balanced Cross-Entropy (BCE) loss effectively addresses class imbalance, a challenge that many traditional methods overlook.
  **Higher Classification Accuracy:** Compared to previous models, including standard CNN and traditional ML methods, proposed C-CNN achieves higher accuracy and robustness, particularly in bearing fault scenarios.
  **Efficiency in Training:** With batch normalization and dropout layers, proposed model improves generalization, reducing overfitting compared to deeper, more computationally expensive architectures.

2. Limitations compared to previous studies:
  **Computational Complexity:** While proposed model enhances performance, it may still have higher computational demands than lightweight ML approaches.
  **Limited Generalization Scope:** Some previous studies incorporate transfer learning or domain adaptation, enabling models to generalize better across different bearing types and operating conditions, an area that requires further improvement in this approach.
  **Interpretability Challenges:** While previous studies leveraging Explainable AI (XAI) techniques provide better model interpretability, proposed model currently lacks a dedicated mechanism for understanding decision-making processes.

**Future work for improvement**:

- **Optimize for Real-Time Applications:** Implement model pruning and quantization to reduce computational load.
- **Enhanced Generalization:** Incorporating more diverse datasets, including data from different bearing types and operating conditions, to improve adaptability.
- **Real-Time Optimization:** Optimizing the model for real-time inference, possibly through model pruning techniques, would make it more applicable in practical settings.
- **Explainable AI Techniques:** Integrating methods to interpret the model’s decisions could provide insights into the fault diagnosis process, increasing trust in the model.
- **Hybrid Models:** Combining the C-CNN with other analytical methods or integrating domain knowledge could further improve performance and robustness.

Addressing these aspects will improve the model’s practical applicability, efficiency, and interpretability in industrial settings.

## Conclusion

This study presented an automated detection approach for diagnosing faults in REBs using a Customized Convolutional Neural Network (C-CNN). This work leveraged high-frequency vibration signals sampled at 12,800 Hz and 5120 Hz as input data to evaluate the impact of sampling rates on diagnostic performance. The study highlights the novel MF block design and the use of the Balanced Cross-Entropy (BCE) loss function as key advancements. The MF block enhances feature extraction by improving feature diversity and robustness, offering a more refined method for capturing fault-related patterns compared to conventional approaches. Additionally, the BCE loss function effectively addresses class imbalance, ensuring more stable and fair learning across different fault categories. While these contributions build upon existing techniques, they introduce a unique combination that enhances classification performance and improves the reliability of bearing fault diagnosis.

The investigation of the analysis showed that the C-CNN model achieved outstanding accuracy, whereas other classifiers demonstrated different levels of performance. The empirical findings clearly established the C-CNN model’s superiority in classifying bearing faults, obtaining a best accuracy of 95% on dataset 1 and 93.5% on dataset 2. More precisely, SqueezeNet, ANN, and Resnet exhibited moderate levels of accuracy, achieving 90.6%, 91.1%, and 93.4% on dataset 1 and 91.0%, 87.0%, and 93.1% on dataset 2. On dataset 1, the accuracy of LSTM, SVM, DBN, RF, CNN, and MSCNN ranged from 85.3% to 89.4%. On dataset 2, the accuracy ranged from 86.5% to 89.5%. Further evaluations assessed the robustness of the C-CNN model under different noise levels and operating conditions, demonstrating its adaptability for real-world applications. Additionally, a computational complexity analysis, including FLOPs estimation, confirmed its suitability for real-time industrial deployment. The findings indicate that the proposed C-CNN model provides a reliable and efficient result for bearing fault classification, qualifying proactive maintenance approaches. By minimizing diagnostic errors, this approach enhances the reliability of industrial machinery, reducing operational downtime and maintenance costs while ensuring continuous system monitoring and efficiency.

## Data Availability

The datasets generated and analyzed during the current study are not publicly available due to institution norms, but are available from the corresponding author on reasonable request.

## Abbreviations

1D-ViT: 1D-vision transformer
AI: Artificial intelligence
ANN: Artificial neural network
BCE: Balanced cross entropy
CBAM: Convolutional block attention module
CCE: Categorical cross entropy
CI: Confidence intervals
CNN: Convolutional neural network
CWRU: Case Western Reserve University
DL: Deep learning
DLGAN: Deep convolutional generative adversarial network
DTM: Diffusion transformation model
ECM: Efficient convolutional module
ELM: Extreme learning machine
FC: Fully connected
FFT: Fast Fourier transform
FGMKE-WOA-MSVM: Fined-grained multi-scale Kolmogorov entropy and whale optimized multi-class support vector machine
FLOP: Floating point operations
GAF: Gramian angular field
GAP: Global average pooling
JNU: Jiangnan University
kNN: K nearest neighbors
MSCNN: Multi-scale convolutional neural network
ReLU: Rectified linear unit
ResNet: Residual network
SVM: Support vector machine
ViT: Vision transformer
WOA: Whale optimization algorithm

## References

[1] Song, X., Liao, Z., Jia, B., Kong, D. & Niu, J. Rolling bearing fault diagnosis under different severity based on statistics detection index and canonical discriminant analysis. *IEEE Access* **11**, 86686–86696 (2023).

[2] Liu, X., Centeno, J., Alvarado, J. & Tan, L. One dimensional convolutional neural networks using sparse wavelet decomposition for bearing fault diagnosis. *IEEE Access* **10**, 86998–87007 (2022).

[3] Wang, Z. *et al.* Early rolling bearing fault diagnosis in induction motors based on on-rotor sensing vibrations. *Measurement (Lond)* **222** (2023).

[4] Grover, C. & Turk, N. A novel fault diagnostic system for rolling element bearings using deep transfer learning on bispectrum contour maps. *Eng. Sci. Technol. ***31** (2022).

[5] Tang, Z., Wang, M., Ouyang, T. & Che, F. A wind turbine bearing fault diagnosis method based on fused depth features in time–frequency domain. *Energy Rep.* **8**, 12727–12739 (2022).

[6] Cui, H., Guan, Y. & Chen, H. Rolling element fault diagnosis based on VMD and sensitivity MCKD. *IEEE Access* **9**, 120297–120308 (2021).

[7] Cui, B., Weng, Y. & Zhang, N. A feature extraction and machine learning framework for bearing fault diagnosis. *Renew. Energy* **191**, 987–997 (2022).

[8] Shen, Q. & Zhang, Z. Fault diagnosis method for bearing based on attention mechanism and multi-scale convolutional neural network. *IEEE Access* **12**, 12940–12952 (2024).

[9] Liao, R. et al. DTM-bearing: A novel framework for speed-invariant bearing fault diagnosis based on diffusion transformation model (DTM). *IEEE Access* **12**, 8875–8888 (2024).

[10] Wang, B., Li, H., Hu, X., Wang, C. & Sun, D. Rolling bearing fault diagnosis based on fine-grained multi-scale Kolmogorov entropy and WOA-MSVM. *Heliyon* **10** (2024). 10.1016/j.heliyon.2024.e27986 PMC10955319 38515657

[11] Gawde, S., Patil, S., Kumar, S., Kamat, P. & Kotecha, K. An explainable predictive maintenance strategy for multi-fault diagnosis of rotating machines using multi-sensor data fusion. *Decis. Anal. J.* **10** (2024).

[12] Li, Q. et al. Fault diagnosis of nuclear power plant sliding bearing-rotor systems using deep convolutional generative adversarial networks. *Nucl. Eng. Technol.*10.1016/j.net.2024.02.056 (2024).

[13] Zhu, J. & Liu, T. Bidirectional current WP and CBAR neural network model-based bearing fault diagnosis. *IEEE Access* **11**, 143635–143648 (2023).

[14] Salunkhe, V. G., Desavale, R. G., Khot, S. M. & Yelve, N. P. A novel incipient fault detection technique for roller bearing using deep independent component analysis and variational modal decomposition. *J. Tribol.* **145** (2023).

[15] Salunkhe, V. G., Khot, S. M., Jadhav, P. S., Yelve, N. P. & Kumbhar, M. B. Experimental investigation using robust deep VMD-ICA and 1D-CNN for condition monitoring of roller element bearing. *J. Comput. Inf. Sci. Eng.* **24** (2024).

[16] Salunkhe, V. G., Khot, S. M., Desavale, R. G. & Yelve, N. P. Unbalance bearing fault identification using highly accurate Hilbert–Huang transform approach. *J. Nondestruct. Eval. Diagn. Progn. Eng. Syst.* **6** (2023).

[17] Salunkhe, V. G., Khot, S. M., Desavale, R. G., Yelve, N. P. & Jadhav, P. S. An integrated dimension theory and modulation signal bispectrum technique for analyzing bearing fault in industrial fibrizer. *J. Nondestruct. Eval. Diagn. Progn. Eng. Syst.* **7** (2024).

[18] Raut, A. S., Khot, S. M. & Salunkhe, V. G. Optimization of geometrical features of spur gear pair teeth for minimization of vibration generation. *J. Vib. Eng. Technol.* **12**, 533–545 (2024).

[19] Raut, A. S., Khot, S. M. & Salunkhe, V. G. Experimental analysis of spur gear pair with geometrical and operating parameters. *J. Nondestruct. Eval. Diagn. Progn. Eng. Syst.* **7** (2024).

[20] Zhang, Y. et al. A new method for diagnosing motor bearing faults based on Gramian angular field image coding and improved CNN-ELM. *IEEE Access* **11**, 11337–11349 (2023).

[21] Xu, P. & Zhang, L. A fault diagnosis method for rolling bearing based on 1D-ViT model. *IEEE Access*10.1109/ACCESS.2023.3268534 (2023).

[22] Vashishtha, G., Chauhan, S., Singh, M. & Kumar, R. Bearing defect identification by swarm decomposition considering permutation entropy measure and opposition-based slime mould algorithm. *Measurement (Lond)* **178** (2021).

[23] Vashishtha, G. & Kumar, R. An amended grey wolf optimization with mutation strategy to diagnose bucket defects in Pelton wheel. *Measurement (Lond)* **187** (2022).

[24] Vashishtha, G. *et al.* Intelligent fault diagnosis of worm gearbox based on adaptive CNN using amended gorilla troop optimization with quantum gate mutation strategy. *Knowl. Based Syst.* **280** (2023).

[25] Kumar, R. *et al.* Fault identification of direct-shift gearbox using variational mode decomposition and convolutional neural network. *Machines* **12** (2024).

[26] Wan, G. & Yao, L. LMFRNet: A lightweight convolutional neural network model for image analysis. *Electronics (Switzerland)* **13** (2024).

[27] Ning, S. & Du, K. Research on intelligent fault diagnosis of rolling bearing based on adaptive resource allocation deep neural network. *IEEE Access* **10**, 62920–62931 (2022).

[28] Han, T., Gong, J. C., Yang, X. Q. & An, L. Z. Fault diagnosis of rolling bearings using dual-tree complex wavelet packet transform and time-shifted multiscale range entropy. *IEEE Access* **10**, 59308–59326 (2022).

[29] Xu, M. & Wang, Y. An imbalanced fault diagnosis method for rolling bearing based on semi-supervised conditional generative adversarial network with spectral normalization. *IEEE Access* **9**, 27736–27747 (2021).

[30] Hou, J., Wu, Y., Ahmad, A. S., Gong, H. & Liu, L. A novel rolling bearing fault diagnosis method based on adaptive feature selection and clustering. *IEEE Access* **9**, 99756–99767 (2021).

[31] Jadon, S. A survey of loss functions for semantic segmentation (2020). 10.1109/CIBCB48159.2020.9277638.

[32] Guruprasad. Notes on Sensitivity, Specificity, Precision, Recall and F1 score. *Analytics Vidhya* https://medium.com/analytics-vidhya/notes-on-sensitivity-specificity-precision-recall-and-f1-score-e34204d0bb9b (2019).

[33] FPR (false positive rate) vs FDR (false discovery rate). *Cross Validated* https://stats.stackexchange.com/questions/336455/fpr-false-positive-rate-vs-fdr-false-discovery-rate (2024).

[34] Calculate Sensitivity, Specificity and Predictive Values in CARET. https://www.geeksforgeeks.org/calculate-sensitivity-specificity-and-predictive-values-in-caret/ (2023).

